# Biomimetic Total Synthesis and Paired Omics Identify an Intermolecular Diels–Alder Reaction as the Key Step in Lugdunomycin Biosynthesis

**DOI:** 10.1021/jacs.5c01883

**Published:** 2025-04-11

**Authors:** Michiel T. Uiterweerd, Isabel Nuñez Santiago, Ana V. Cunha, Remco W. A. Havenith, Chao Du, Le Zhang, Helga U. van der Heul, Somayah S. Elsayed, Adriaan J. Minnaard, Gilles P. van Wezel

**Affiliations:** † Stratingh Institute for Chemistry, University of Groningen, Nijenborgh 7, Groningen 9747 AG, The Netherlands; ‡ Institute of Biology, Leiden University, Sylviusweg 72, Leiden 2333 BE, The Netherlands; § Faculty of Engineering, University of Antwerp, IPRACS, Groenenborgerlaan 171, Antwerpen 2020, Belgium; ∥ Zernike Institute for Advanced Materials, University of Groningen, Nijenborgh 4, Groningen 9747 AG, The Netherlands; ⊥ Department of Chemistry, University of Ghent, Krijgslaan 281, S3, Gent 9000, Belgium; # NIOO-KNAW, Netherlands Institute of Ecology, Droevendaalsesteeg 10, Wageningen 6708 PB, The Netherlands

## Abstract

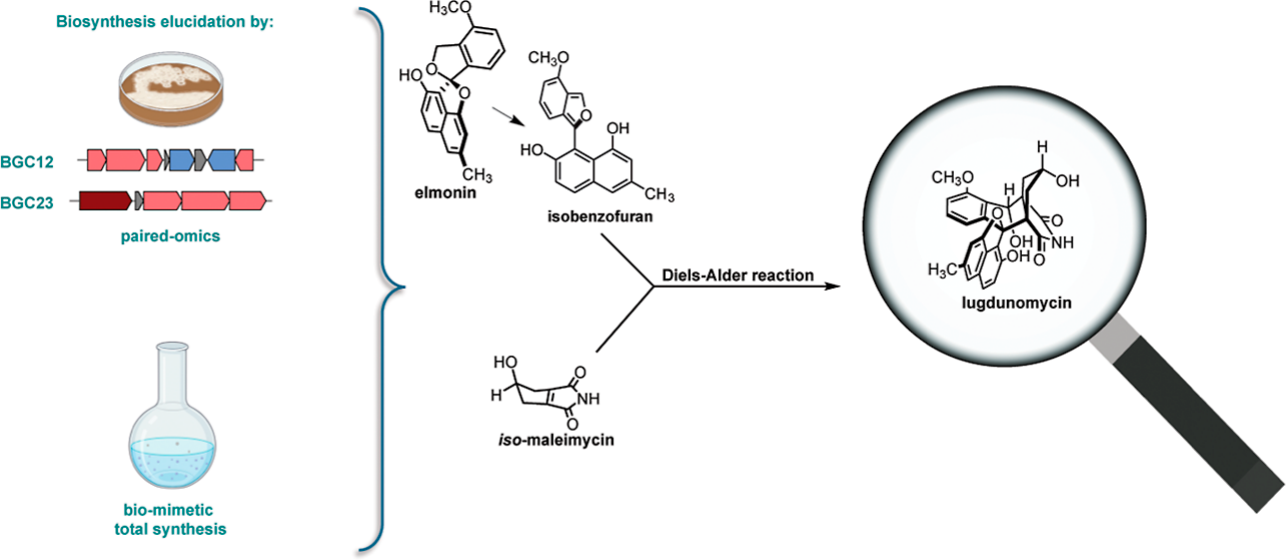

Microbial natural products are the basis of the majority of clinical drugs, where the discovery of truly novel structural scaffolds to fill the discovery pipelines is a prerequisite. Lugdunomycin is a highly rearranged angucycline polyketide produced by *Streptomyces* sp. QL37, with an enigmatic biosynthetic pathway. Here we show that lugdunomycin is formed by a rare intermolecular Diels–Alder reaction, with elmonin as a masked diene and *iso*-maleimycin as a dienophile. Genomics, mutational analysis, and heterologous expression revealed that the biosynthesis of the substrates is encoded by distinct biosynthetic gene clusters (BGCs), whereby elmonin is specified by an angucycline BGC, while the biosynthesis of *iso*-maleimycin is encoded by a BGC for a β-lactone-like compound. Biomimetic total synthesis of lugdunomycin showed that the Diels–Alder reaction leads to the production of a diastereomer of lugdunomycin as the main product in vitro. The diastereomeric ratio of the in vitro Diels–Alder reaction shifted toward lugdunomycin in the presence of proteinaceous material, suggesting that the in vivo Diels–Alder reaction is templated. Alphafold modeling and experimental data suggest that GarL could potentially function as a Diels–Alder template in lugdunomycin biosynthesis. The requirement of distinct biosynthetic pathways and complex chemical reactions indicates the challenges we face in discovering new chemical space.

## Introduction

Natural products from microorganisms, particularly Actinobacteria, have been the traditional source of our medicines including the majority of the clinical antibiotics,^1^ but we have likely explored only a fraction of their chemical diversity. Compared to the vast number of known molecules in NPAtlas,^2^ an estimated 3% of biosynthetic diversity has been accessed.^3^ Thus, there is huge potential to discover new natural product scaffolds that may form the basis for our future medicines.^4^

Lugdunomycin (**1**) is a highly rearranged angucycline derivative produced by the soil-derived *Streptomyces* sp. QL37.^5^ Angucyclines comprise a very large family of glycosylated natural products.^6^ Their aglycones, referred to as angucyclinones, originate from a benz[*a*]anthracene framework synthesized through the polyketide pathway.^6a,7^ They collectively constitute the most extensive category of natural products derived from a type II polyketide synthase (PKS), boasting over 400 identified members, approximately 45% of which exhibit biological activity.^8^ Many of the compounds have antimicrobial and/or anticancer activity; however, many other bioactivities have been described, such as vasodilator, glutamate receptor agonist, platelet aggregation inhibitor, or antidiabetic.^6a^ A major reason for the strong interest in the angucyclines is the occurrence of highly diverse chemical scaffolds, whereby over a hundred different compounds may be produced by the same *Streptomyces* strain.^5,9^ This still offers great opportunities to discover new synthetic and biosynthetic routes and, thus, enrich the chemical space for drug discovery.

Lugdunomycin is very different from typical angucyclinones, featuring a rare benzaza[4,3,3]propellane-6-spiro-2′-2H-naphtho[1,8-bc]furan core structure.^5^ A putative biosynthetic pathway was proposed for the biosynthesis of lugdunomycin. While the biosynthesis of the core angucycline backbone of lugdunomycin is encoded by a type II PKS biosynthetic gene cluster (BGC), it is unclear how the final product is synthesized. Indeed, the lugdunomycin BGC is highly similar to other angucycline BGCs, while these strains are not able to produce lugdunomycin. We previously hypothesized that the final steps in lugdunomycin biosynthesis may require the combination of an angucyclinone and maleimycin^7^ or its constitutional isomer *iso*-maleimycin (**2**). Both *iso*-maleimycin **2** and maleimycin were produced by chemical synthesis, and comparative metabolomics analysis demonstrated that *iso*-maleimycin, and not maleimycin, is produced by *Streptomyces* sp. QL37.^10^ Understanding the biosynthesis of lugdunomycin is of major importance for better insights into how angucyclines and angucyclinones are synthesized. Its highly unusual structure likely requires a complex biosynthetic pathway.

In this paper, we resolved the final steps in lugdunomycin biosynthesis via a combination of biomimetic total synthesis, paired omics, and computational simulation. We harnessed the power of biomimetic total synthesis to synthesize the highly complex lugdunomycin in vitro and, in doing so, provide evidence that lugdunomycin is produced from *iso*-maleimycin and elmonin in an intermolecular Diels–Alder reaction. This type of reaction is rare in natural product biosynthesis. Our work also shows that *iso*-maleimycin and elmonin are produced from different biosynthetic gene clusters, which explains why lugdunomycin is rarely found in nature, despite the fact that many streptomycetes produce angucyclines.

## Results and Discussion

### Bioinformatics Combined with Quantitative Proteomics Points at Multiple Gene Clusters That Encode Lugdunomycin Biosynthesis

We previously showed that lugdunomycin **1** is detected exclusively in solid-grown cultures of *Streptomyces* sp. QL37, and at very low levels; 7.5 L of minimal medium (MM) agar afforded some 0.6 mg of the compound, which was nevertheless sufficient to elucidate its structure.^5^ Still, the expression of metabolites in liquid-grown cultures is the preferred method to enable elicitation approaches and paired omics. These approaches facilitate correlating gene expression profiles to metabolite production so as to allow the identification of the biosynthetic genes for the compound(s) of interest.^11^ In an attempt to improve lugdunomycin biosynthesis in liquid-grown cultures, *Streptomyces* sp. QL37 was grown in liquid MM with glucose, xylose, rhamnose, fructose, arabinose, or galactose as the sole carbon source, and extracts were prepared from culture supernatants. Analysis via liquid chromatography combined with mass spectrometry (LC–MS/MS) identified classical angucyclinones in all samples, as well as rearranged-angucyclinones such as elmonin^8^ (Figure S1a). Interestingly, lugdunomycin was detected only in supernatants of galactose-grown cultures (Figure S1b).

To establish which genes/proteins relate to lugdunomycin biosynthesis, we applied a proteomining approach,^11a^ which is based on the fact that expression levels of biosynthetic proteins encoded by BGCs correlate very well with the levels of the metabolites they produce. The strong correlation between the two allows connection of biosynthetic proteins to their cognate metabolite(s).^11a^ For this, quantitative proteomics was performed to determine the most differentially expressed proteins in mycelia grown in MM with galactose (inducing conditions) and MM with glucose (noninducing). After 3 days of growth, biomass was harvested and snap-frozen in liquid nitrogen. Subsequent quantitative proteomics analysis was performed on four replicate samples per growth condition, yielding 2444 quantifiable proteins, of which 42 were differentially expressed with a log^2^ fold change higher than 2, between galactose- and glucose-grown cultures (Table S1). Expectedly, most proteins that exhibited enhanced differential expression in galactose-grown cultures were primarily associated with galactose metabolism. However, a subset of genes stood out that belonged to a BGC that was annotated as BGC23 in the *Streptomyces* sp. QL37 genome.

**Figure 1:**
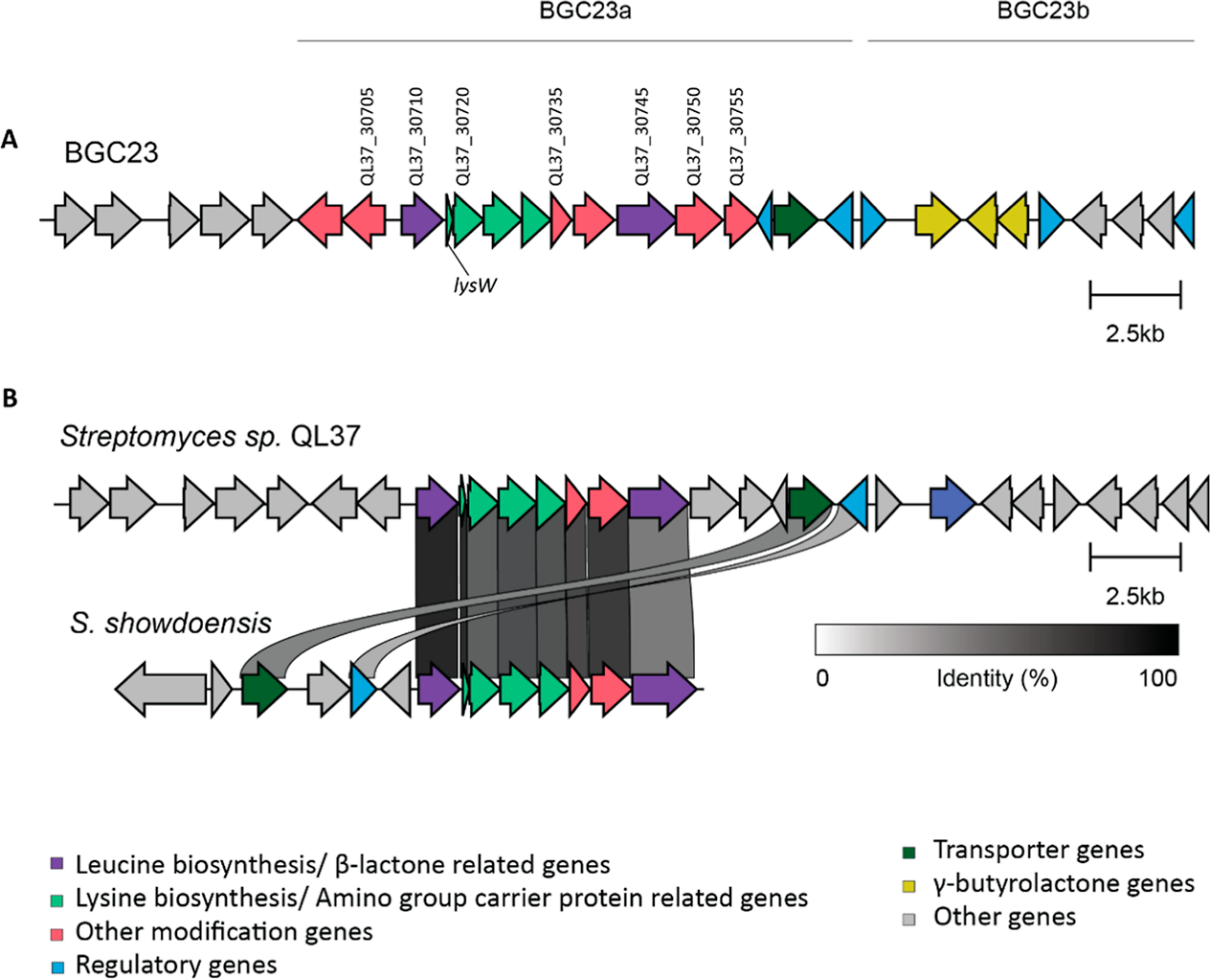
Genomic structure of BGC23 in *Streptomyces* sp. QL37. (a) Prediction of the BGC by antiSMASH.^16^ The BGC consists of two subclusters, BGC23a and BGC23b. Colors indicate the predicted gene function as indicated. Genes that were identified as upregulated in galactose-grown cultures via proteomics are indicated with the gene name. (b) Comparison of BGC23a of *Streptomyces* sp. QL37 with the putative maleimycin BGC of *S. showdoensis* ATCC 15227. Genes sharing a minimum of 40% nucleotide sequence identity are shown by connections between the BGCs. Note the high homology in the gene organization and sequence.

BGC23 (Figure 1a) was annotated by antiSMASH^12^ as consisting of two subclusters, namely, BGC23a (for a β-lactone-like compound) and BGC23b (for a γ-butyrolactone). Subcluster 23a includes a gene encoding a 2-isopropylmalate synthase (QL37_30710) and a gene encoding an AMP ligase (QL37_30720), similar to those seen in β-lactone BGCs responsible for belactosin and cystargolide production.^13^ Furthermore, BGC23a also includes a gene for an orthologue of LysW (QL37_30715), a carrier protein involved in lysine synthesis.^14^ Recent studies have demonstrated that these proteins play a key role in the biosynthesis of nonproteogenic building blocks in natural products, such as vazabitide A.^15^ BGC23a shows significant homology in terms of sequence identity and gene arrangement with the putative maleimycin BGC of *Streptomyces showdoensis* ATCC 15227 (Figure 1b).

### BGC23a Is Required for the Biosynthesis of *iso*-Maleimycin

To gain further insights into the precise compound(s) produced by BGC23a and the possible role in lugdunomycin biosynthesis, a knockout mutant of *Streptomyces* sp. QL37 was created, in which BGC23 was inactivated. To achieve this, genes *lysW* (QL30715) and *rimK* (QL30720), which were predicted to encode an amino carrier protein and its cofactor, respectively,^15^ were selected for gene deletion. The two genes were together replaced by the apramycin resistance cassette (*aacC4*) using homologous recombination. For this, a knockout strategy was applied that is based on the unstable multicopy plasmid pWHM3.^17^ The correct mutant was verified by PCR.

**Figure 2:**
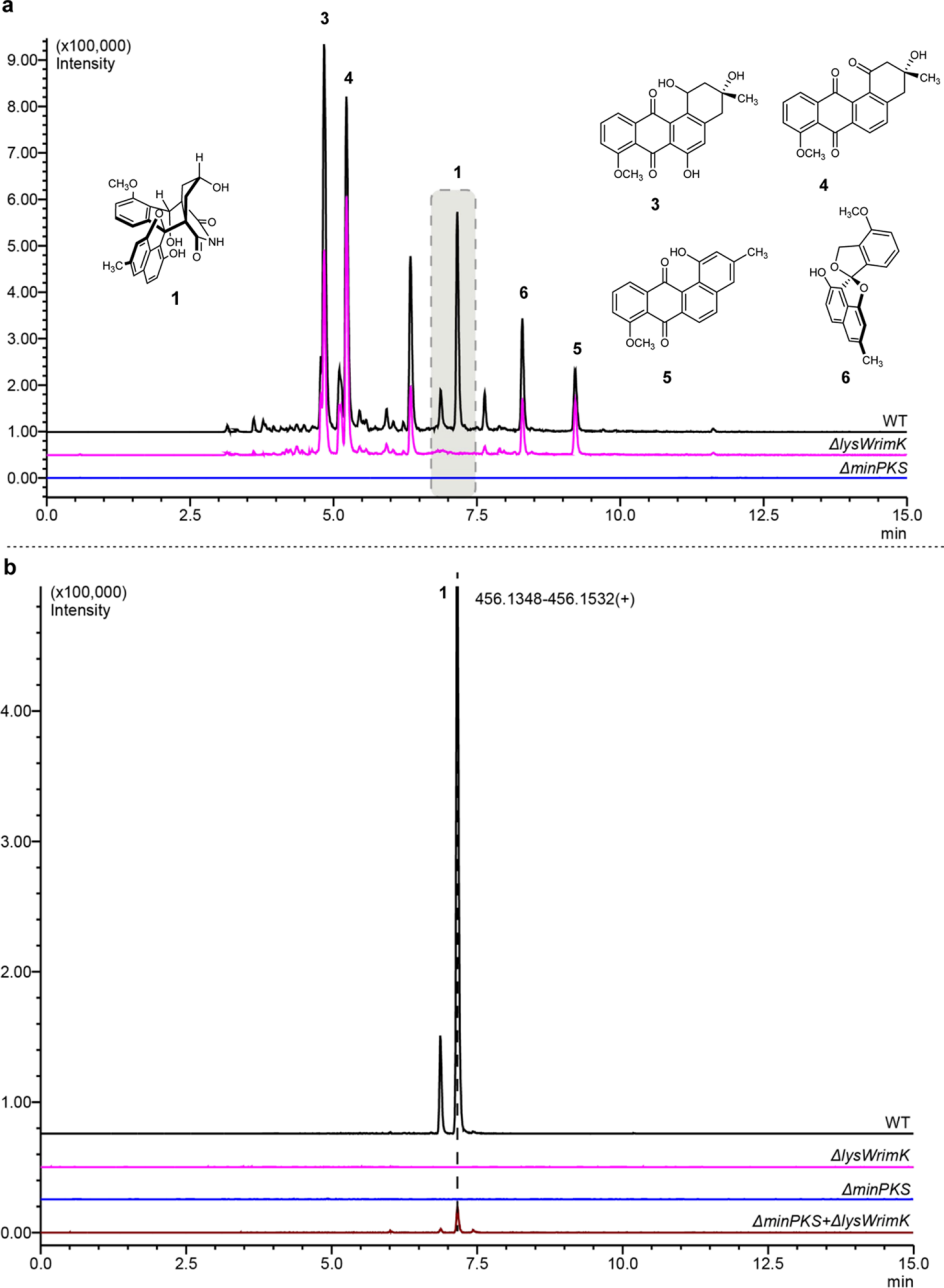
Comparative LC–MS chromatograms of wild-type *Streptomyces* sp. QL37 and mutant strains. (a) Comparison of the *lysWrimK* mutant (pink) to the *minPKS* mutant (blue) and the parental strain *Streptomyces* sp. QL37 (black). The chromatograms represent the extracted ion chromatogram (XIC) of some typical angucyclinones (compound **3:** 355.1144–355.1216; **4:** 337.1036–337.1104; and **5:** 319.0928–319.0992), elmonin (**6:** 321.1035–321.1165), and lugdunomycin (**1**) produced by *Streptomyces* sp. QL37. (b) Extracted ion chromatogram (XIC, selected [M + H–H_2_O]^+^) for lugdunomycin in extracts obtained from solid fermentation of *Streptomyces* sp. QL37 wild-type (WT, black), BGC23a mutant (Δ*lysWrimK*, pink), BGC12 mutant (Δ*minPKS,* blue), and combination of BGC12 and BGC23a spores (Δ*minPKS*, Δ*lysWrimK*, brown).

To establish if the knockout strain was still capable of producing *iso*-maleimycin, we compared the metabolomics profiles with chemically synthesized *iso*-maleimycin,^10^ dissolved in methanol, which serves as a standard due to the challenges in detecting *iso*-maleimycin directly using LC–MS. In this way, *iso*-maleimycin was readily detected as its methanol adduct [M + CH_3_OH + H]^+^ (Figure S2). Subsequently, extracts from both parental strains of *Streptomyces* sp. QL37 and its Δ*lysWrimK* mutant were analyzed for the ability to produce *iso*-maleimycin. The strains were cultivated for 7 days on MM agar supplemented with 0.5% mannitol and 1% glycerol, and extracts were prepared from the culture supernatant. Although *iso*-maleimycin was readily detected in extracts from the wild-type strain, its BGC23a knockout strain failed to produce *iso*-maleimycin (Figure S3), thus implying its involvement in *iso*-maleimycin biosynthesis. Next, the effect of the inactivation of BGC23a on the biosynthesis of angucyclines and lugdunomycin was studied. For this purpose, mutant Δ*lysWrimK* and its parent *Streptomyces* sp. QL37, along with the minimal PKS mutant of BGC12, which fails to produce lugdunomycin or angucyclinones,^5^ were cultivated in the same way as mentioned above, and extracts were prepared. LC–MS analysis (Figure 2a) revealed the presence of canonical angucyclines, elmonin, and lugdunomycin in extracts from the parental strain. Expectedly, no angucyclines or elmonin were detected in the minimal PKS mutant. Importantly, although the Δ*lysWrimK* mutant did produce angucyclines, lugdunomycin was not detected (Figure 2a). Thus, the proteomics, metabolomics, and gene deletion experiments collectively suggested a pivotal role for BGC23a in the later stage of lugdunomycin biosynthesis. Further analysis of the metabolome of the PKS null mutant revealed the biosynthesis of *iso*-maleimycin (Figure S3), while the compound was not produced in the Δ*lysWrimK* mutant. This strongly suggests that the biosynthesis of *iso*-maleimycin depends on BGC23a, while it is independent of the angucycline biosynthetic pathway. From here onward, BGC23a is referred to as the *iso*-maleimycin BGC. The involvement of a reaction partner derived from another BGC in lugdunomycin production is further supported by two lines of evidence. First, heterologous expression of BGC12 in *Streptomyces coelicolor* M1152 resulted in the biosynthesis of angucyclines, while lugdunomycin itself was never detected.^18^ Second, overexpression of the angucycline gene cluster (BGC12) resulted in strong overproduction of angucyclinones but did not improve lugdunomycin production.^18^

### Chemical Complementation and Heterologous Expression Support the Requirement of Two BGCs for Lugdunomycin Biosynthesis

Our data show that inactivation of either BGC12 (for the biosynthesis of angucyclinones) or BGC23a (for the biosynthesis of *iso*-maleimycin) prevents lugdunomycin production. This suggests that the biosynthesis of lugdunomycin requires reaction partners from both BGCs. If indeed the two BGCs independently produce the substrates for the final step in lugdunomycin biosynthesis, the individual mutants lacking either BGC12 or BGC23a should be able to complement each other chemically and thus produce lugdunomycin. To test this hypothesis, spores of the two strains were mixed in a 1:1 ratio and cultured for 7 days on MM agar supplemented with 0.5% mannitol and 1% glycerol and subsequently extracted with ethyl acetate for analysis by LC–MS (Figure 2b). As controls, BGC12 null mutant Δ*minPKS*, BGC23a null mutant Δ*lysWrimK*, and their parent were grown separately and treated the same way. Importantly, while no lugdunomycin was produced by either of the mutants grown alone, cocultivation of the mutants restored lugdunomycin production (Figure 2b). This further supports the notion that both BGCs are necessary for the biosynthesis of lugdunomycin.

To test if the two BGCs indeed suffice for lugdunomycin biosynthesis, we expressed both BGCs in *S. coelicolor* M1152.^19^ For this, we used *S. coelicolor* M1152 which already contains a copy of the *lug* gene cluster.^18^ This strain was shown to produce angucyclines but failed to produce lugdunomycin. Importantly, additional introduction of BGC23 into *S. coelicolor* M1152+*lug* indeed afforded production of lugdunomycin **1** (Figure S4a) as well as *iso*-maleimycin **2** (Figure S4b). Taken together, all of these results demonstrate that both BGC12 and BGC23 are required for the production of lugdunomycin.

### Phylogenomic Analysis Identifies Other Streptomycetes as Potential Lugdunomycin Producers

The requirement of two separate BGCs for lugdunomycin biosynthesis serves as a beacon to search for other strains that produce lugdunomycin and/or related compounds. For this, we queried all available *Streptomyces* and *Kitasatospora* genomes to explore the co-occurrence within a single strain of a BGC homologous to the *iso*-maleimycin BGC together with an angucycline BGC. The genomes were downloaded from RefSeq, and the work was performed using the ALICE compute resources provided by Leiden University. The quality of genomes with more than 400 contigs was considered too low and therefore filtered out. In addition to the genomes obtained from the public databases, 101 genomes from our in-house MBT collection (Microbial BioTechnology Department of Leiden University) were then added to the collection. A phylogenetic tree was made for this collection of genomes using PhyloPhlAn (version 3.0.60),^20^ based on the protein sequence of 370 core genes (Figure S5). To predict the BGCs for natural products in these genomes, they were analyzed using antiSMASH (version 6.1.1).^16^ This predicted the presence of in total of 71,624 potential BGCs. All of these BGCs were dereplicated using a modified BiG-MAP script 10.5281/zenodo.10978017. 34582 BGCs showed <40% overall similarity in the translated amino acid sequences, and these BGCs were therefore treated as different clusters. These clusters were submitted to BiG-SCAPE (version 1.1.4)^21^ to generate sequence similarity networks. BiG-SCAPE analyzed the protein motifs of the core as well as the modifying enzymes of each cluster to compare the BGCs. With a cutoff of 0.2 in distance, clusters of similar BGCs for both BGC12 and BGC23a were identified. Adding the similar BGCs that were filtered out by the BiG-MAP algorithm, 201 strains likely contained an angucycline BGC with significant similarity to BGC12, while 27 contained a BGC similar to BGC23a. Importantly, of these, five strains contained gene clusters with high similarity to both BGCs (Figure S5). These are *Streptomyces* sp. QL37 itself and in addition *Streptomyces* sp. MBT70 (from our in-house collection), *Streptomyces* sp. SM11, *Streptomyces filamentosus*, and *Streptomyces shenzhenensis*. This suggests that the biosynthesis of lugdunomycin-like molecules is not unique to *Streptomyces* sp. QL37.

### From In Vivo to In Vitro: Biomimetic Synthesis of Lugdunomycin from Its Predicted Substrates

**Figure 3:**
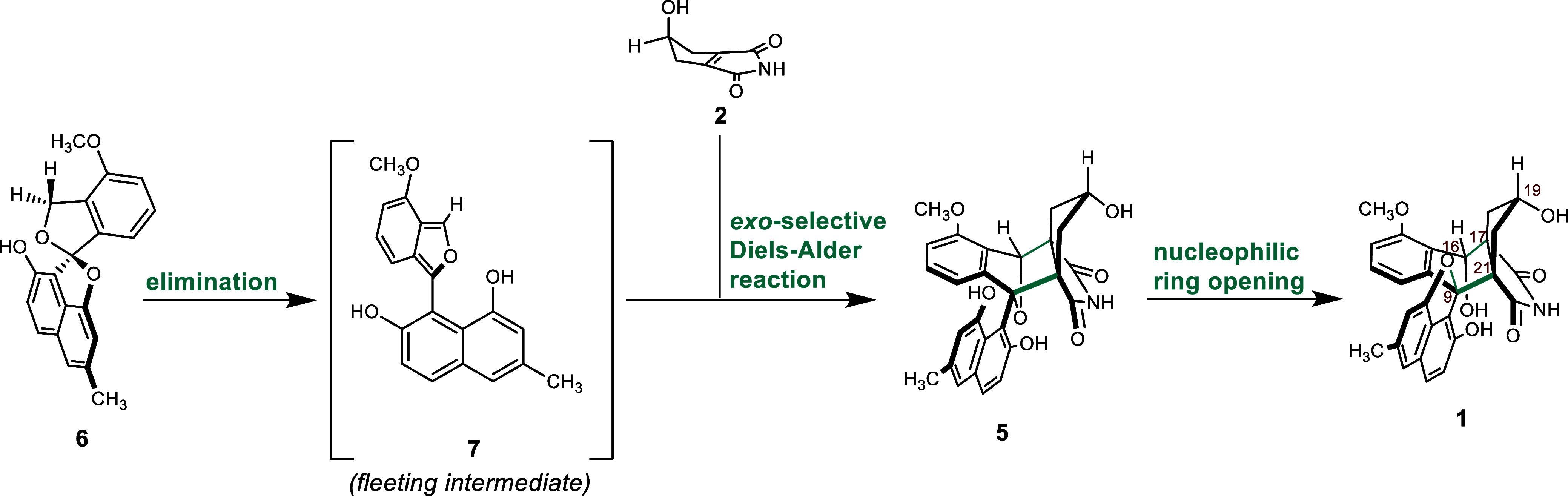
Lugdunomycin and its proposed bio(mimetic)synthesis from elmonin and *iso*-maleimycin.

Taken together, all of our in vivo genetic and metabolomics data, as well as the bioinformatics analyses, provided evidence that the biosynthesis of lugdunomycin requires two BGCs. We thereby anticipated that BGC12 ensures the biosynthesis of an angucycline-derived diene, while BGC23a ensures the biosynthesis of a dienophile, most likely *iso*-maleimycin. To investigate this further, we embarked on a biomimetic synthesis route to see if lugdunomycin could be produced in vitro. We previously synthesized *iso*-maleimycin.^10^ Searching for a diene for the Diels–Alder reaction, we realized that such a diene could originate from elmonin **6** by an intramolecular elimination reaction. Elmonin is a *C*-ring-cleaved, rearranged angucyclinone polyketide that had been independently isolated by two groups from *Streptomyces* spp,^22^ though not from *Streptomyces* sp. QL37. We achieved the chemical synthesis of elmonin in a 13-step synthesis, as described elsewhere.^23^ Importantly, LC–MS showed that elmonin is produced by *Streptomyces* sp. QL37 (Figure S8), and thus, the putative dienophile (*iso*-maleimycin) and the precursor of the putative masked diene (elmonin) for the proposed intermolecular Diels–Alder reaction are de facto present in *Streptomyces* sp. QL37. The hypothesized pathway is presented in Figure 3.

### Elmonin Is a Masked Diene

**Figure 4:**
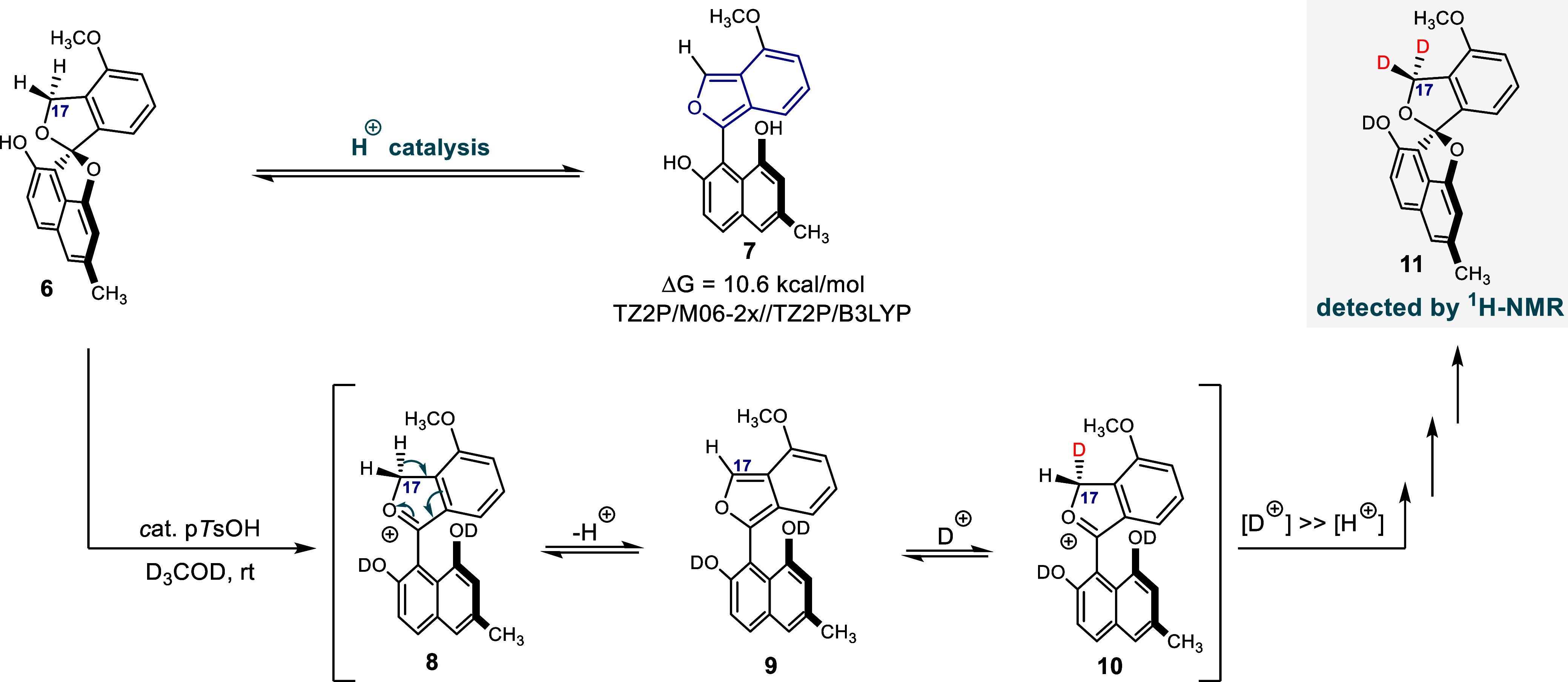
Acid treatment of elmonin leads to deuteration of C17, a result of reversible isobenzofuran formation.

Elmonin **6** can be viewed as a 1-alkoxy 1,3-dihydroisobenzofuran derivative. These compounds are precursors of isobenzofuran derivatives, generated upon base or Brønsted acid-promoted 1,4-elimination.^24^ Isobenzofurans, in turn, are highly reactive dienes in Diels–alder reactions.^25^ Next to studies on the reactivity of isobenzofurans in situ*,* isobenzofuran formation has been applied in the chemical synthesis of galtamycinone^26^ and in the synthesis of epithuriferic acid methyl ester.^27^ Considering the structure of elmonin, we anticipated it could act as a masked diene, and we would be able to generate the corresponding isobenzofuran by treatment with acid. In an exploratory experiment, a solution of **6** in D_3_COD was treated with a small amount of *p*-TsOH (Figure 4).

After 24 h, a significant decrease in the intensity of the signal at 5.30 ppm (corresponding to H17) was observed in ^1^H NMR (Figure S9). This is the result of reversible deprotonation at C17 and supports the hypothesis that **6** is a masked isobenzofuran. Deuteration of the spiro-ketal leads to ring opening and elimination to give isobenzofuran **9**. As this process is reversible, the net result is deuteration at C17. A mechanistically similar formation of a quinodimethane under the same conditions was initially considered, but density functional theory (DFT) calculations showed that isobenzofuran **7** is more than 15 kcal/mol lower in energy than the corresponding quinodimethane (QDM, in Figure S21). This can be underpinned with qualitative chemical arguments; in **7**, aromaticity is partly retained, whereas in a quinodimethane, this is not the case.

### Diels–Alder Reaction of *iso*-Maleimycin and Elmonin Yields Lugdunomycin and Its Diastereomers

**Figure 5:**
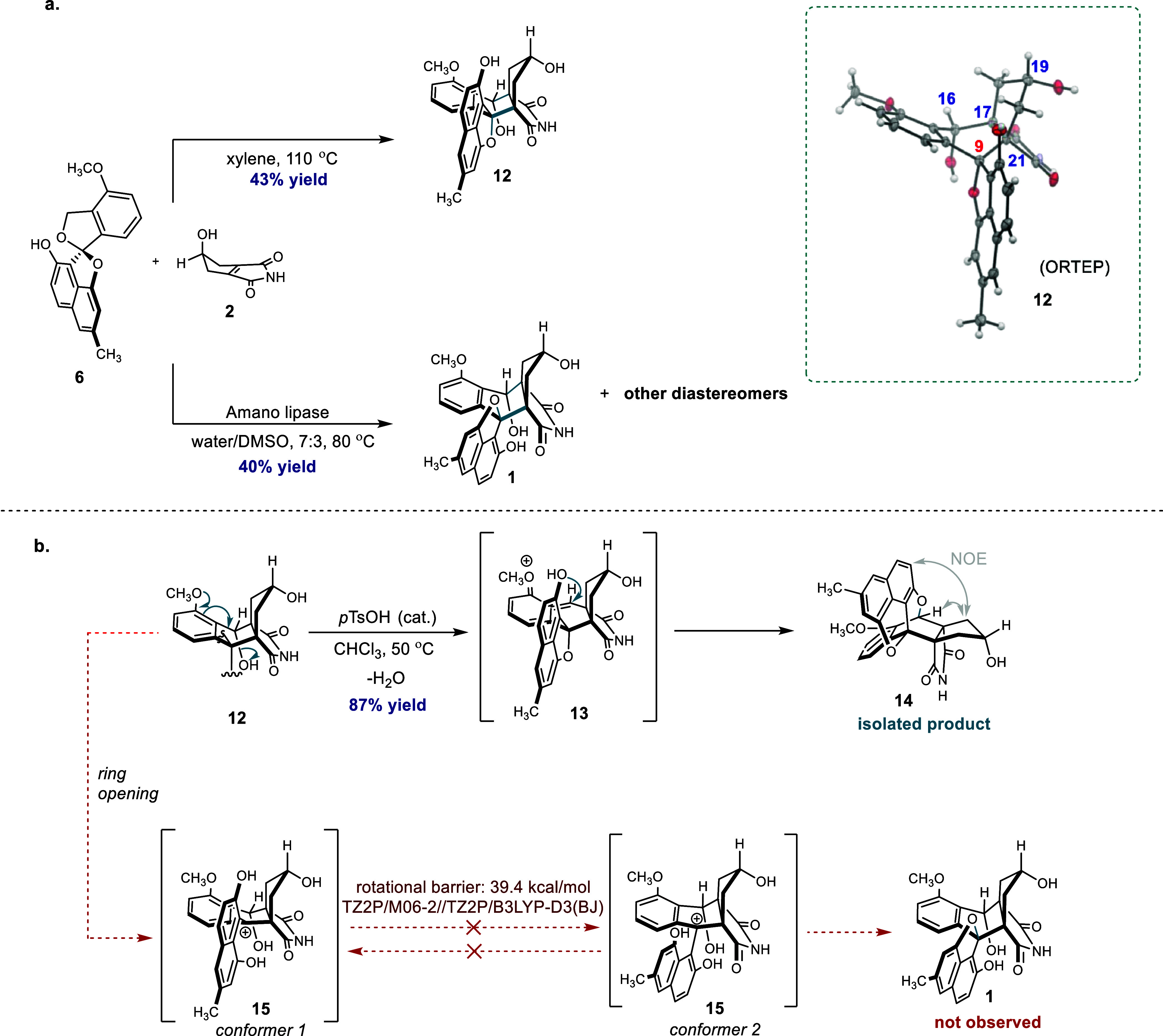
Synthesis experiments. (a) Synthesis of *epi*-lugdunomycin and lugdunomycin via the Diels–Alder reaction of elmonin and *iso*-maleimycin. (b) Attempted isomerization of *epi*-lugdunomycin **12** leads to dehydration instead of lugdunomycin formation.

Upon heating a mixture of elmonin **6** and *iso*-maleimycin **2** in *m*-xylene in a sealed tube at 110 °C, one main Diels–Alder product was formed. However, close inspection of the ^1^H- and ^13^C NMR spectra revealed that the product obtained was not lugdunomycin but instead an isomer (**12**; Figures 5a and S12–S14).

With the help of X-ray crystallography, we established that four out of five stereocenters (16S*, 17R*, 19S*, 21S*) had identical relative configurations as compared to lugdunomycin (Figures 5 and S7). However, the quaternary, fifth stereocenter at C9, being R* in lugdunomycin,^6^ was S* in the synthetic material. This makes compound **12** the C9-epimer of lugdunomycin.

Attempts to convert **12** by isomerization into the desired **1** were unsuccessful (Figure 5b). We anticipated that protonation of the ether oxygen at C9 would lead to ring opening, providing relatively stable carbenium ion **15**. Bond rotation would then provide the other conformer of **15**, which can subsequently undergo ring closure, leading to **1**. However, when **12** was treated with *p*TsOH, no epimerization product was observed. Instead, it afforded product **14** in high yield, apparently formed due to electronically and entropically favored dehydration. In addition, DFT calculations showed that the required bond rotation is not feasible because of hindered rotation (Figure S23). Although the Diels–Alder reaction mainly afforded the production of **12**, analysis of the reaction mixture by LC–MS also revealed small quantities of lugdunomycin, accompanied by up to six other products with identical mass and similar retention times, which probably represent diastereomers.

We were puzzled by the fact that the Diels–Alder reaction in the bacterial host consistently led to lugdunomycin **1**, while during the in vitro reaction, 9-*epi*-lugdunomycin **12** was obtained as the main product. This conflicts with the idea that lugdunomycin is biosynthesized in vivo in an undirected manner, i.e., without a steering factor. Involvement of an enzyme, a Diels–Alderase, was considered less likely at this stage, also because lugdunomycin crystallized from the isolate as the racemate. However, all attempts to mimic this reaction chemically, including a study of the effects of solvent, temperature, pH, and the addition of surfactants or salts, led to just minor changes in the ratio of the diastereomeric products (Table S4).

In order to elucidate the cause of the discrepancy between the in vivo and in vitro synthesis of lugdunomycin and its diastereomers, all eight possible reaction pathways were calculated for the Diels–Alder reaction between *iso*-maleimycin and the isobenzofuran derived from elmonin (Tables 1 and S6). From a mechanistic point of view, the dienophile can approach the isobenzofuran from two different faces, arbitrarily called the “front face” and “back face”. Four diastereomers can be formed when the dienophile approaches from the “front face” and another four when the dienophile approaches from the “back-face” (Table 1). Front face path 1 leads to lugdunomycin **P1** (**1**), and “back face” path 5 leads to 9-*epi*-lugdunomycin **P5** (**12**). The other paths lead to the other diastereomers. At the Density Functional Theory (DFT) level, the relative Gibbs energy (Δ*G*) of the stationary points in paths 1–8 was calculated using the Amsterdam Modeling Suite (AMS) software package.^28^ Thermochemical properties were calculated at the TZ2P/M06-2x//TZ2P/B3LYP-D3(BJ) level of theory, using a similar “hybrid” approach as described by Tang et al. (Supporting Information Section 7).^29^

**Table 1:**
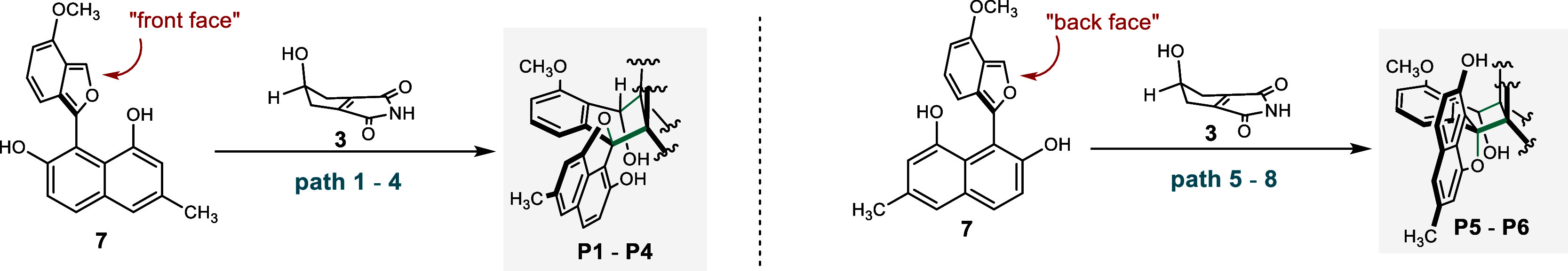

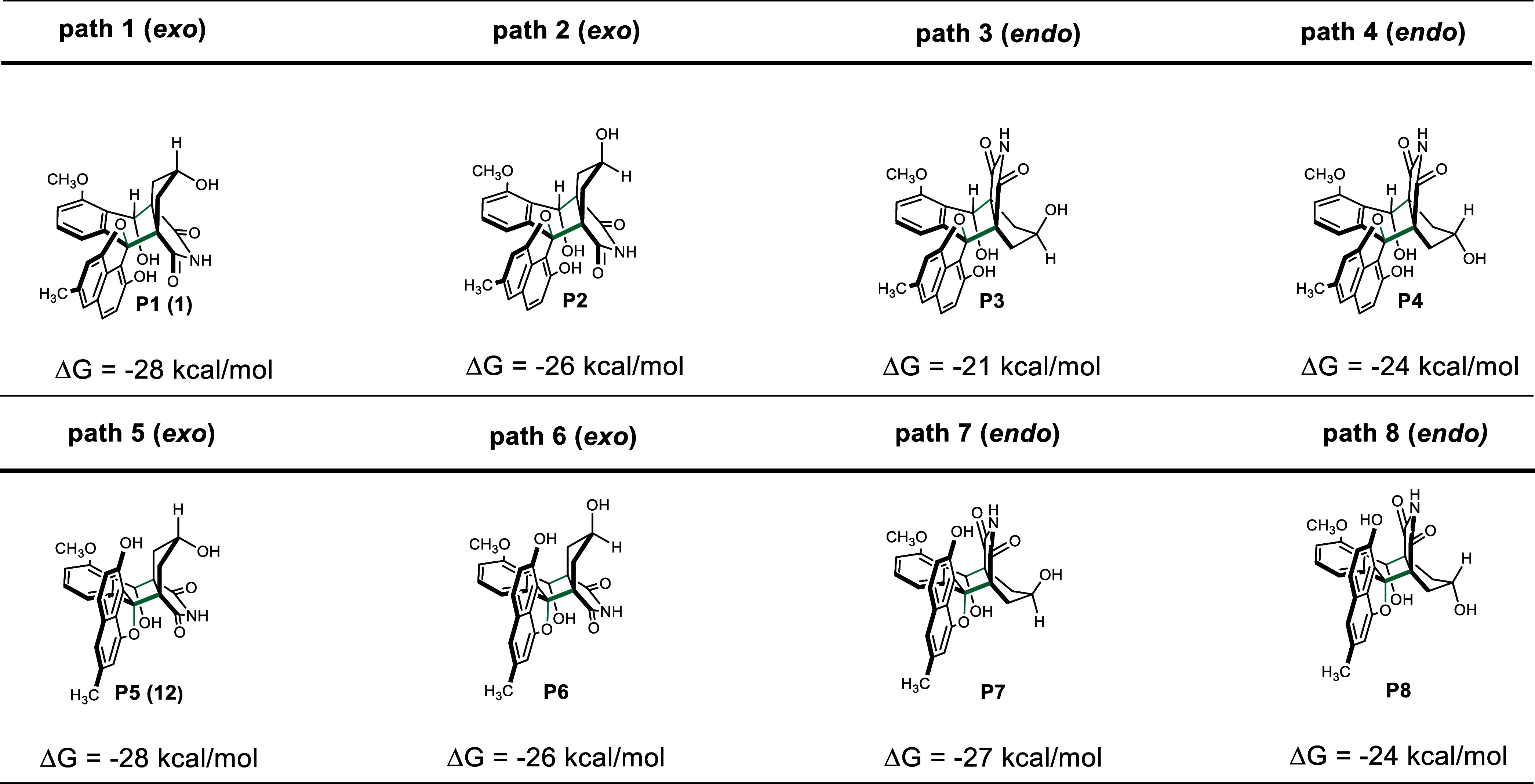
Calculated Δ*G* Values for the Formation of Lugdunomycin and Its Diastereomers

Evaluation of path 1 revealed that the Diels–Alder reaction likely is a true concerted [4 + 2] cycloaddition, having a moderate asynchronous character. Computation of the charge density and placing an electrostatic potential show that the charges are approximately equally distributed over the maleimide portion of the transition state. An alternative ionic two-step mechanism is therefore unlikely (Figure S22). With regard to the thermochemical properties, all of the paths seem feasible at ambient temperatures as the Δ*G*^‡^ values for the reactions range from 21 to 26 kcal/mol. Furthermore, the formation of all diastereomeric intermediates is thermodynamically favored with Δ*G* values ranging from −0.3 to −14 kcal/mol (not shown, Table S6). Also, the formation of all final products, after the ring closure, is highly favored with Δ*G* values ranging from −21 for **P3** to −28 kcal/mol for **P1** and **P5**. Molecular modeling suggested that the large energy gap between the intermediates and the products is due to strain relief upon ring opening of the bridgehead ether moiety. These computations are in full agreement with the experimental observations; e.g., up to eight different isomeric products are observed by HPLC-MS in the in vitro Diels–Alder reaction (Table S4). The small energy differences between the transition states further underline the agreement between the theoretical modeling and the experimental data. This explains the observation that changes in solvent and temperature slightly changed the diastereoselectivity of the reaction (Tables S4 and S6). It also imposed the conclusion that there is an additional “steering factor” leading to lugdunomycin production with high selectivity in *Streptomyces* sp. QL37.

### Protein-Based Templating Promotes Lugdunomycin Synthesis In Vitro

An important property of a Diels–Alderase would consist of a templating effect by increasing the effective molarity of both *iso*-maleimycin and elmonin, which is needed considering the low concentrations of the reactants in the bacterium. Furthermore, suitable positioning of both reactants would explain the stereoselectivity of the reaction, not necessarily disputing the observation that lugdunomycin is formed with low or small enantioselectivity, considering its isolation as racemic crystals. In order to mimic this templating effect, we added proteins with a hydrophobic pocket or cleft to the in vitro Diels–Alder reaction. *iso*-Maleimycin and, in particular, elmonin have a limited solubility in water, so offering a hydrophobic “shelter” would possibly bring both compounds together. Upon adding proteins like BSA to the reaction, we immediately saw a significant and positive effect; the diastereomeric ratio shifted in favor of lugdunomycin, although not to such an extent that it became the main product. After extensive screening and optimization, it turned out that the use of Amano Lipase from *Pseudomonas fluorescens* in phosphate-buffered saline and 30% DMSO at 80 °C shifted the ratio of **1**/**12**/other from 2:6:1 to 2:2:1 (Table S4). This is a dramatic shift in favor of lugdunomycin and offers support for the need for a template and for the possible presence of a Diels–Alderase in the producer strain *Streptomyces* QL37. Scale-up of this protein-assisted reaction at 0.4 mmol provided 74 mg of lugdunomycin-diastereomers after column chromatography. Preparative HPLC subsequently provided 8.4 mg of pure lugdunomycin **1** which in all aspects was identical to the lugdunomycin isolated from cultures of *Streptomyces* sp. QL37 (Supporting Information Sections 3 and 5).

Although intermolecular Diels–Alderases are rare in nature, the rapid advancement of omics technologies and bioinformatics has led to the identification of an increasing number of Diels–Alderases, particularly those that catalyze intermolecular Diels–Alder reactions.^30^ However, to the best of our knowledge, there has not been a report of an intermolecular Diels–Alderase in bacteria. To see if we could identify a candidate Diels–Alderase in *Streptomyces* sp. QL37, we searched for enzymes with homology to annotated Diels–Alderases. This revealed GarL, which is an orthologue of the fungal enzyme macrophomate synthase (MPS). MPS was initially reported as a putative Diels–Alderase^31^ but was shown to act as an efficient aldolase.^32^ Initial experiments showed that deletion of *garL* led to reduced biosynthesis of lugdunomycin in vivo, while genetic complementation of the mutant with a wild-type copy of *garL* restored lugdunomycin production to wild-type levels (see Section 2 of the Supporting Information, Figure S6E). A combination of Alphafold modeling and MD simulations showed that both *iso*-maleimycin and the isobenzofuran formed from elmonin can be stabilized in the active site of GarL, thereby facilitating the Diels–Alder reaction (Figure S6A–D). Given its primary role as a 5-keto-4-deoxy-d-glucarate aldolase, similar to MPS, GarL candidates as an “opportunistic Diels–Alderase”.

## Conclusions

In this work, we show that the angucyclinone elmonin (**6**),^8^ biosynthesized from BGC12, and *iso*-maleimycin (**2**) which is independently biosynthesized from BGC23a, are the substrates required for biosynthesis of the highly rearranged angucyclinone lugdunomycin (**1**). These two biosynthetic intermediates provide the diene and the dienophile of an intermolecular Diels–Alder reaction that leads, after subsequent ring closure, to **1**. The observation that the two substrates originate from two different BGCs that cooperate to generate lugdunomycin presents an important concept. The canonical view involves a single gene cluster that is responsible for the biosynthesis of a molecule or a family of molecules. The requirement of multiple BGCs is seen more often in fungi,^33^ such as for the biosynthesis of the meroterpenoids austinol and dehydroaustinol in *Aspergillus nidulans*.^34^ Multi-BGC biosynthetic pathways have also been observed in Actinobacteria,^35^ and this concept requires more attention. For one, heterologous expression is an important tool for the analysis of BGCs and their cognate natural products. However, expressing BGC12 would result in production of angucyclines, but lugdunomycin would have remained undiscovered as its biosynthesis also requires *iso*-maleimycin derived from BGC23a.

Intermolecular Diels–Alder reactions are rare in biosynthesis. Among the very few well-documented cases are the biosynthesis of paracaseolide A^36^ and the flavonoid chalcomoracin,^37^ with the latter catalyzed by a bona fide Diels–Alderase. Elmonin (**6**), an 1-alkoxy 1,3-dihydroisobenzofuran, acts as a masked isobenzofuran that is formed upon an acid-catalyzed intramolecular elimination reaction and undergoes a Diels–Alder reaction with the dienophile *iso*-maleimycin. This Diels–Alder reaction is impressive from a reactivity point of view. It involves a tetrasubstituted alkene which is a poor dienophile because of its steric encumbrance. Isobenzofuran is a reactive diene but is present only as a fleeting intermediate, in particular in a cellular context. Starting from two achiral substrates, and after subsequent ring-opening-ring closure, the Diels–Alder reaction produces lugdunomycin, which contains five stereocenters and is formed with high diastereoselectivity. Biosynthetic intermolecular Diels–Alder reactions can be spontaneous, such as reported for the Diels–Alder reaction in the biosynthesis of paracaseolide A,^36^ or catalyzed by an enzyme, such as in the biosynthesis of chalcomoracin.^38^ We showed that the Diels–Alder reaction that forms the final step in lugdunomycin biosynthesis is templated. This follows from the high activation energy and the low concentration of both reactants, exacerbated by the thermodynamically uphill formation of isobenzofuran. The main product of the nontemplated reaction (which occurs in vitro at higher temperatures and at much higher concentrations) is not lugdunomycin but a diastereomer. Upon the addition of proteins with a hydrophobic pocket or cleft, the reaction can be steered to produce lugdunomycin. The addition of micelle-forming surfactants did not induce this effect, and taken together, this provides support for the possible involvement of an enzyme. Alphafold modeling, MD simulations, and genetic experiments suggested GarL as a possible candidate for the Diels–Alder reaction. More detailed experiments, including enzyme assays, are required to ascertain this.

The role of cross-talk and regulatory mechanisms in the biosynthesis of lugdunomycin should be further investigated. To the best of our knowledge, this is the first case whereby biomimetic synthesis, computational chemistry, and genomics have been combined, allowing us to shed important new light on the biosynthesis of a molecule with a highly complex chemical scaffold. This emphasizes the need to consider unconventional biosynthetic relationships and molecular interactions that may drive the synthesis of complex secondary metabolites. In addition, our work shows that unraveling the genetic elements and regulatory mechanisms governing the cooperation between BGCs, combined with chemical mechanistic insights, can help provide novel insights into the biosynthetic machinery responsible for the biosynthesis of natural products of interest. Such novel biosynthetic insights are also key to pathway engineering and synthetic biology approaches, enabling the production of (families of) compounds at higher yields or the generation of novel analogues with enhanced properties. These approaches should help scientists further explore and uncover the complexity of microbial secondary metabolism.

## Supporting Information Available

Genetics and paired-omics experiments on *Streptomyces* sp. QL37; computational experiments of GarL as a possible Diels–Alderase in Streptomyces sp. QL37; synthetic experimental procedures; experiments with elmonin; NMR spectra of synthetic compounds 1, 12, and 14; HRMS spectra of synthetic compounds 1, 12, and 14; and Density Functional Theory (DFT) calculations (PDF)
