# Supplementary material for: Biomimetic Total Synthesis and Paired Omics Identify an Intermolecular Diels–Alder Reaction as the Key Step in Lugdunomycin Biosynthesis
Source: J Am Chem Soc. 2025 Apr 11;147(16):13764–74. doi: 10.1021/jacs.5c01883 (PMC12022981; doi:10.1021/jacs.5c01883)
Supplement: Supplementary file 1 — ja5c01883_si_001.pdf [file ja5c01883_si_001.pdf]

SUPPORTING INFORMATION

belonging to the manuscript

Biomimetic total synthesis and paired omics identify an intermolecular Diels-Alder reaction as the key step in lugdunomycin biosynthesis

Michiel T. Uiterweerd[a,#], Isabel Nuñez Santiago[b,#], Ana V. Cunha[c], Remco W. A. Havenith[a,d,e], Chao Du[b], Le Zhang[b], Helga U. van der Heul[b], Somayah Elsayed[b], Adriaan J. Minnaard[a,‡] and Gilles P. van Wezel[b,f,†]

[a]University of Groningen, Stratingh Institute for Chemistry, Nijenborgh 7, 9747 AG, Groningen, The Netherlands.

[b]Leiden University, Institute of Biology, Sylviusweg 72, 2333 BE, Leiden, The Netherlands.

[c]University of Antwerp, Faculty of Engineering, IPRACS, Groenenborgerlaan 171, 2020 Antwerpen, Belgium.

[d]University of Groningen, Zernike Institute for Advanced Materials, Nijenborgh 4, 9747 AG, Groningen, The Netherlands.

[e]University of Ghent, Department of Chemistry, Krijgslaan 281, S3, 9000 Gent, Belgium.

[f]Netherlands Institute of Ecology, NIOO-KNAW, Droevendaalsesteeg 10, 6708 PB, Wageningen, The Netherlands.

# These authors contributed equally to the work.

[‡]Corresponding authors: A.J.Minnaard@rug.nl, +31 50 36 34258; g.wezel@biology.leidenuniv.nl, +31 71 527 4310

Contents:

Section 1: Genetics and paired-omics experiments on *Streptomyces* sp. QL37 ... 3
Microorganisms and culturing conditions ... 3
Preparation of crude extracts and metabolite profiling ... 3
Metabolomics Methods ... 3
Statistical Analysis of LC-MS data ... 4
Proteomics Methods ... 5
Heterologous expression of BGC23 in *Streptomyces coelicolor* M1152+*lug* ... 6
Creation of mutants of *Streptomyces* sp. QL37 ... 6
Section 2: Computational experiments of GarL as a possible Diels-Alderase in *Streptomyces* sp. QL37 . 16
Molecular Dynamics Simulations. ... 19
Analysis. ... 19
Section 3: Synthetic experimental procedures ... 20
Comparative NMR table of synthetic lugdunomycin 1 ... 23
Analytical scale optimisation of lugdunomycin synthesis ... 24
Crystal data of compound 12 ... 26
Section 4: Experiments with elmonin ... 28
Section 5: NMR spectra of synthetic compounds 1, 12 and 14 ... 30
Section 6: HRMS spectra of synthetic compounds 1, 12, 14 ... 39
Section 7: Density Functional Theory (DFT) calculations ... 41
Computational method ... 44
Optimized geometries ... 44
References ... 91

Section 1: Genetics and paired-omics experiments on *Streptomyces* sp. QL37

Microorganisms and culturing conditions

All media and routine *Streptomyces* techniques were done following routine protocols[1]. *Streptomyces* sp. QL37 was obtained from soil collected in the Qinling mountains of the People's Republic of China, as previously described[2, 3]. The strain was deposited in the Centraal Bureau voor Schimmelcultures (CBS) in Utrecht, The Netherlands, with the accession number 138593. For solid cultivation, *Streptomyces* sp. QL37 was grown confluently on minimal media (MM) agar plates containing 1% glycerol and 0.5 % mannitol (w/v) as the carbon sources. Liquid fermentation was performed in 100 mL Erlenmeyer flasks (Fisher Scientific) were 20 mL of liquid minimal medium (MM without agar) supplemented with R5 trace elements solution[1] (4mL/L), glycerol (1% w/v) and/or mannitol (0.5% w/v), after autoclaving. Other carbon sources were added at 1% (w/v) prior inoculation.

Preparation of crude extracts and metabolite profiling

In the case of solid fermentations, after seven days of growth at 30 °C the agar plates were cut into small pieces and soaked in 25 ml of ethyl acetate for 12 h[4]. After evaporation extracts were dissolved in methanol (MeOH) to a final concentration of 1 mg/mL. Spent media of liquid-grown cultures were extracted after five days of incubation using HP-20 beads (Supelco) previously soaked in MeOH overnight. Approximately 1 g of HP-20 beads was added to each flasks and incubated O/N while shaking. Extracts were prepared as described[5] and dissolved in methanol (MeOH) to a final concentration of 1 mg/mL.

For metabolite profiling of the crude extracts, liquid chromatography-tandem mass spectrometry (LC-MS/MS) analysis was performed using a Shimadzu Nexera X2 ultra high-performance liquid chromatography (UPLC) system, equipped with a photodiode array detector (PDA), coupled to a Shimadzu 9030 QTOF mass spectrometer, equipped with an electrospray ionization (ESI) source unit, which included a calibrant delivery system (CDS). For details see Supplementary Section 2.

To allow the detection of *iso*-maleimycin using LC-MS in the complex bacterial extract, we developed the following method. As standard we used 0.01 mg/mL of synthesized *iso*-maleimycin[6] dissolved in methanol. Then, a multiple reaction monitoring (MRM) method was developed targeting the $[M+CH_3OH+H]^+$ ion of *iso*-maleimycin (186.0759) and the characteristic MS/MS fragment of 109.0283.

Metabolomics Methods

Liquid chromatography-tandem mass spectrometry (LC-MS/MS) was performed using a Shimadzu Nexera X2 ultra high-performance liquid chromatography (UPLC) system, equipped with a photodiode array detector (PDA), coupled to a Shimadzu 9030 QTOF mass spectrometer, equipped with an electrospray ionization (ESI) source unit, which included a calibrant delivery system (CDS). For the LC separation, 2 µL of the sample was injected into a Waters Acquity HSS C18 column (1.8 µm, 100 Å, 2.1 × 100 mm). The column temperature was maintained at 30 °C, and the separation was carried out at a flow rate of 0.5 mL/min. Solvent A consisted of 0.1% formic acid in H_2O, while solvent B comprised 0.1% formic acid in acetonitrile (ACN). The gradient elution profile started with 5% B for 1 min, followed by a linear increase from 5% to 85% B over 9 min, then a steep increase to 100% B over 1 min, and finally, an isocratic hold at 100% B for 4 min. To re-equilibrate the column, it was set to 5% B for 3 min before the subsequent run[7,8].

The PDA acquisition was conducted within the wavelength range of 200–600 nm, with a scan rate of 4.2 Hz and a slit width of 1.2 nm. The temperature of the flow cell was maintained at 40 °C throughout the analysis. All samples were analyzed in positive polarity mode using a data-dependent acquisition method. This involved acquiring full scan MS spectra within the m/z range of 100–1700 at a scan rate of 10 Hz, with intelligent data (ID) enabled. Subsequently, two data-dependent MS/MS spectra within the same m/z range and scan rate were obtained for the two most intense ions detected in each scan. Collision-induced dissociation (CID) was employed for ion fragmentation with a fixed collision energy (CE) of 20 eV. To avoid re-analysis of previously fragmented ions, they were excluded from selection for 1 s before being eligible for fragmentation again. The parameters used for the ESI source were as follows: interface voltage of 4 kV, interface temperature of 300 °C, nebulizing gas flow rate of 3 L/min, and drying gas flow rate of 10 L/min[8].

Statistical Analysis of LC-MS data

Prior to conducting statistical analysis, mzXML files—converted via Shimadzu LabSolutions Postrun Analysis—were imported into MZmine 2.31[9] and processed following previously described methods. The aligned peak list was then exported as a CSV file for statistical analysis. MetaboAnalyst[10] was utilized for statistical analysis, where log transformation and Pareto scaling were applied to the data. Model quality was assessed based on *R2* and *Q2* values. To compare the intensity of individual mass features across multiple growth conditions, one-way ANOVA was performed, followed by post hoc analysis using Tukey's honest significant difference (HSD) test[11].

Proteomics Methods

Proteomics samples were prepared as previously described [12, 13]. To obtain samples for proteomics analysis, bacterial cultures were pelleted and lysed in 100 µL lysis buffer (4% SDS, 100 mM Tris-HCl pH 7.6, 50 mM EDTA) and disrupted by sonication. Total protein was precipitated using the chloroform-methanol method[14], and the proteins dissolved in 0.1% RapiGest SF surfactant (Waters) at 95°C. The protein concentration was measured at this step using the BCA method. Protein samples were then reduced by adding 5 mM DTT and incubate at 60°C for 30 min, followed by thiol group protection with 21.6 mM iodoacetamide incubation at room temperature in dark for 30 min. Then 0.1 µg trypsin (recombinant, proteomics grade, Roche) per 10 µg protein was added, and samples were digested at 37°C overnight. After digestion, trifluoroacetic acid was added to 0.5% and samples were incubated at 37°C for 30 min followed by centrifugation to degrade and remove RapiGest SF. Peptide solution containing 6 µg peptide was then cleaned and desalted using STAGE-Tips[15]. Briefly, 6 µg of peptide was loaded on a conditioned StageTip with 2 pieces of 1 mm diameter C18 disk (Empore, product number 2215), washed twice with 0.5% formic acid solution, and eluted with elution solution (80% acetonitrile, 0.5% formic acid). Acetonitrile was then evaporated in a SpeedVac. Final peptide concentration was adjusted to 40 ng·µL-1 using sample solution (3% acetonitrile, 0.5% formic acid) for analysis.

The desalted peptides solution weres separated on an UltiMate 3000 RSLCnano system set in a trap-elute configuration with a nanoEase M/Z Symmetry C18 100 Å, 5 µm, 180 µm × 20 mm (Waters) trap column for peptide loading/retention and nanoEase M/Z HSS C18 T3 100 Å, 1.8 µm, 75 µm × 250 mm (Waters) analytical column for peptide separation. Mobile phase A composed of 0.1% formic acid (FA) in ULC-MS grade H2O (Biosolve), while mobile phase B composed of 0.1% FA, 10% H2O in ULC-MS grade acetonitrile (ACN, Biosolve). The flow gradients used for analysis was a shallow 113 min gradient of mobile phase A and B controlled by a flow sensor at 0.3 µL·min-1. The gradient was programmed with linear increment from 1% to 5% B from 0 to 2 min, 5% to 13% B from 2 to 63 min, 13% to 22% B from 63 to 85 min, 22% to 40% B from 85 to 104 min, 90% at 105 min and kept at 90% to 113 min.

The eluent was introduced by electro-spray ionisation (ESI) via the nanoESI source (Thermo) to QExactive HF (Thermo Scientific). The QExactive HF was operated in positive mode with data dependent acquisition. The MS survey scan was set with mass range 350-1400 m·z-1 at 120,000 resolution. For individual peaks, the data dependent intensity threshold of 2.0 × 104 was applied for triggering an MS/MS event, isotope exclusion on and dynamic exclusion was 20 s. Unassigned, +1 and charges >+8 were excluded with peptide match mode preferred. For MS/MS events, the loop count was set to 10, isolation window at 1.6 m·z-1, resolution at 15,000, fixed first mass of 120 m·z-1, and normalised collision energy (NCE) at 28 eV. The

obtained raw data was processed using MaxQuant version 2.1.0.0b[16] with default label free quantification settings.

Heterologous expression of BGC23 in *Streptomyces coelicolor* M1152+*lug*

For the heterologous expression of BGC23 into optimized host *S. coelicolor* M1152 or its derivative containing the *lug* cluster (BGC12)[17], the BGC was divided into three fragments, each amplified by PCR using the chromosome of *Streptomyces* sp. QL37 as the template. Fragment 1 was amplified with primers BGC23_P1 and BGC23_P2, fragment 2 with primers BGC23_P3 and BGC23_P4, and fragment 3 with primers BGC23_P5 and BGC23_P6 (Table S2). The final construct was assembled by Gibson assembly to combine fragments 1, 2, and 3 with the vector pSET152, which integrates at the øC31 attachment site on the *S. coelicolor* chromosome[12] to create pSET152-BGC23. To generate pMS82-BGC23, the insert was obtained as an HpaI-EcoRV fragment from pSET152-BGC23 and cloned into EcoRV-digested pMS82[18]. The integrity of the constructs was verified using the restriction enzyme combinations KpnI, KpnI and SpeI, ClaI and EcoRV. Non-methylated pMS82-BGC23 was isolated from *E. coli* strain ET12567 and introduced into *S. coelicolor* M1152+*lug*[17] via protoplast transformation. Correct transformants were checked via PCR using oligonucleotides LysWRimK_check_R2 and WK_check_F primers (Table S2).

Creation of mutants of *Streptomyces* sp. QL37

1. *Creation of the lysWrimK null mutant*

The deletion of the *lysWrimK* genes was done based on a previously reported method[19]. Briefly, the construct for gene disruption was obtained by amplification of ~1.5 kb regions up- and downstream of the chromosome of *Streptomyces* sp. QL37 using primer pairs 1+2 and 3+4 (Table S2) and cloned as EcoRI-XbaI and XbaI-BamHI fragments into a derivative of the unstable multi-copy plasmid pWHM3[20] that harbours oriT to allow transference by conjugation. The apramycin resistance cassette aac(3)IV flanked by *loxP* sites was then inserted in-between using an engineered XbaI site. The correct knock-out construct was transformed to the methylase-deficient strain *E. coli* ET12567/pUZ8002[21], and subsequently introduced into *Streptomyces* sp. QL37, following the protocol as described[1]. The correct mutant was selected by resistance to apramycin (50 µg/mL) and sensitivity to thiostrepton (10 µg/mL). Polymerase chain reactions (PCR) were performed on a T100 Thermal Cycler (Bio-Rad, Hercules, CA, USA). The ZR Plasmid Miniprep-Classic kit (Zymo Research, Irvine, CA, USA) was used for plasmid extraction.

2. *Creation of a garL null mutant and genetic complementation*

For deletion of *garL*, the -1102/+39 and +765/+1801 regions relative to the translational start site of *garL* were amplified by PCR from the *Streptomyces* sp. QL37 genome using primer pairs P1_FR1_garL and P2_FR1_garL; P3_FR2_garL and P4_FR2_garL, respectively (Table 2). From this point onwards we followed exactly the same procedure as for the *lysWrimK* mutant above, generating a *garL*mutant. The mutant was verified by PCR using oligonucleotides seq_F_permE::GarL and seq_R_permE::GarL as described. To achieve constitutive expression of the *garL* gene, and genetically complement the *garL* mutant, the gene was amplified from the genome using the primers garL-T0_REV and garL_FOR listed in Table 2 and cloned downstream of the *ermE* promoter into pSET152, which had been digested with NdeI and XbaI. The integrity of the resulting constructs was confirmed through Sanger sequencing using the primers seq_F_permE::GarL and seq_R_permE::GarL. The constructs were isolated from *E. coli* JM109 and subsequently transformed into the methylase-deficient strain *E. coli* ET12567/pUZ8002 to facilitate conjugation with *Streptomyces* sp. QL37.

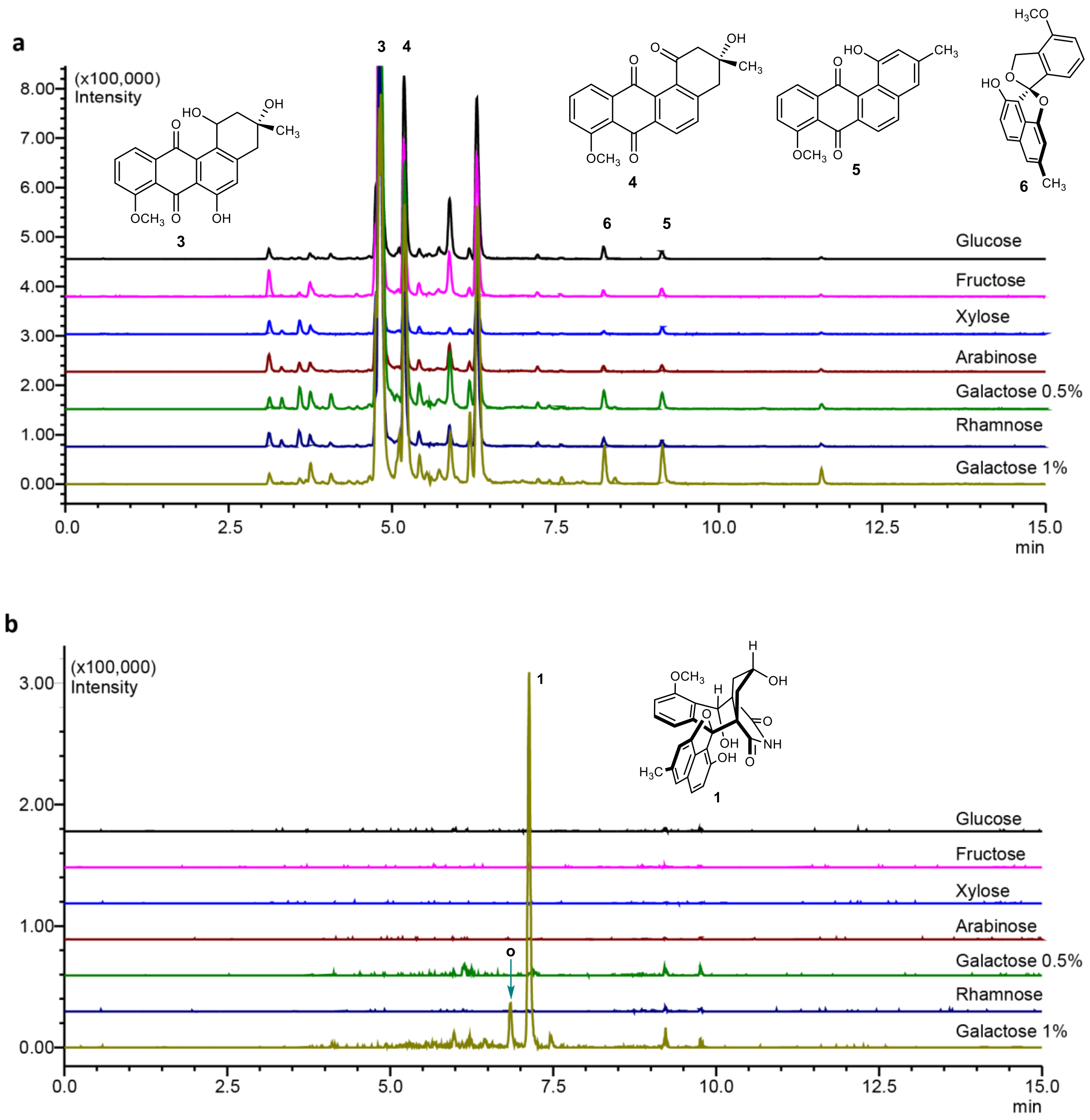

Figure S1. Carbon-source dependent production of angucyclines and lugdunomycin. a. Extracted ion chromatogram (XIC) for some previously identified angucyclinones produced by *Streptomyces* sp. QL37. The XIC correspond to the following m/z: 355.117 (**3**), 339.123 (**4**), 319.096 (**5**), 321.111 (**6**) with a tolerance of 20 ppm. **b**. XIC for 456.144, the m/z of **1** $\{M+H-H_2O\}^+$ identified in *Streptomyces* sp. QL37 extracts. *Streptomyces* cultures were grown for 7 days in liquid MM supplemented with carbon sources as indicated. Note that lugdunomycin was only observed when cultures contained larger amounts of galactose. It is expected that the small peak in the 1% galactose chromatogram, indicated with the letter **o**, is a diastereomer of **1**.

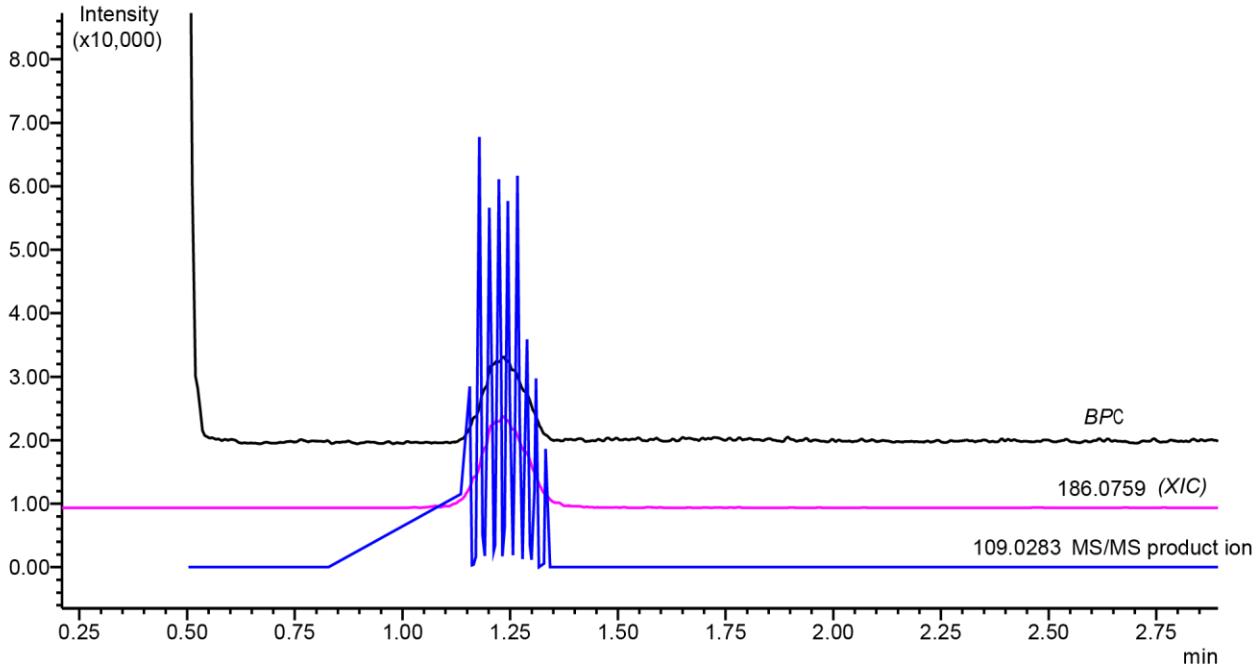

Figure S2. LC-MS chromatogram of chemically synthesized *iso*-maleimycin. LC-MS chromatogram depicting the analysis of a standard solution of chemically synthesized *iso*-maleimycin **2** (0.01 mg/mL, black) dissolved in MeOH. We noted that *iso*-maleimycin reacts with MeOH and the molecular ion $[M+CH_3OH+H]^+$ was established at m/z 186.0759 (pink), and a unique product ion at m/z 109.0283 was selected for detection (blue). The retention time (RT) for *iso*-maleimycin was determined to be 1.25 min.

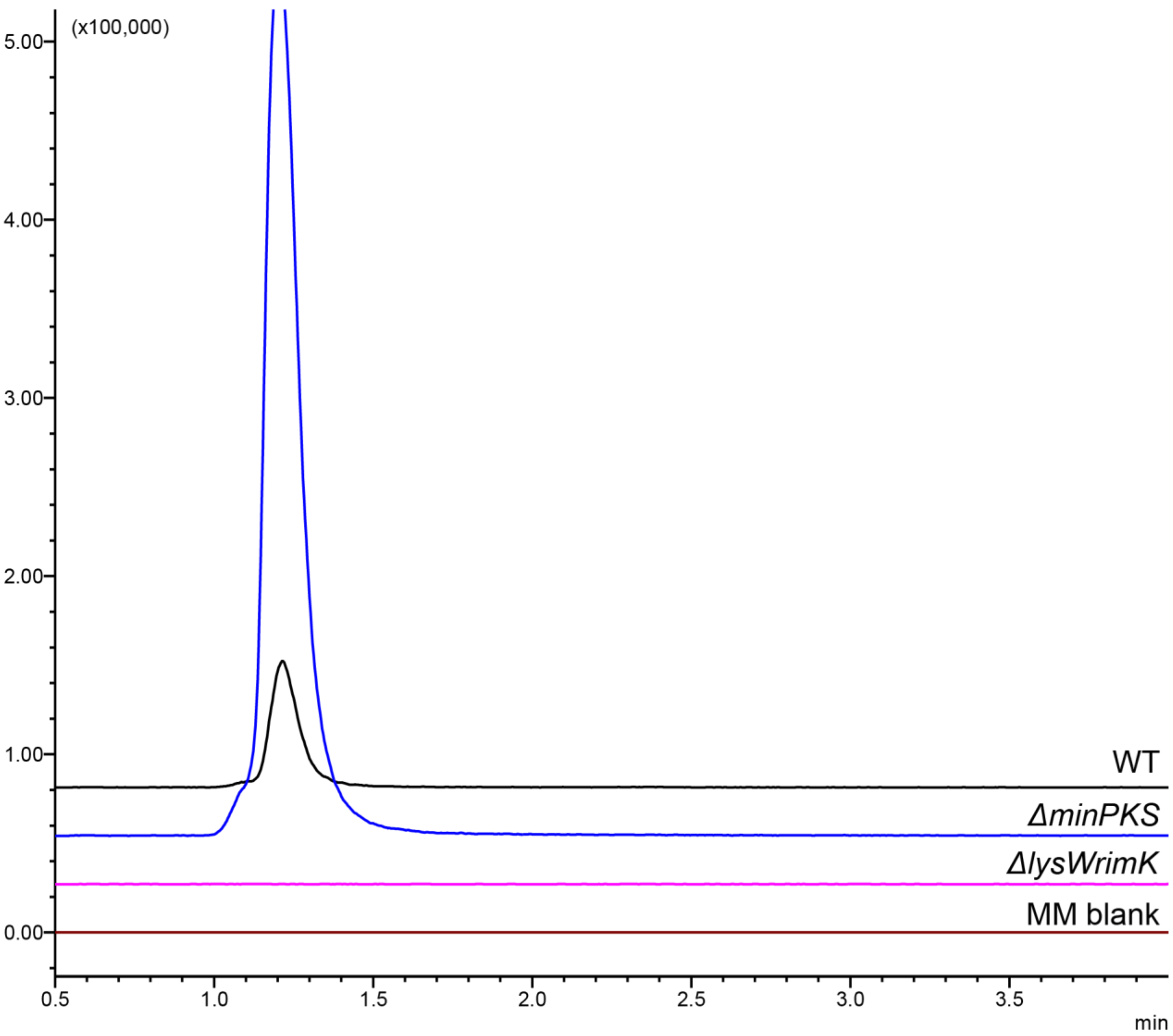

Figure S3. LC-MS/MS chromatograms of *iso*-maleimycin in *Streptomyces* sp. QL37 wild-type, Δ*minPKS* and Δ*lysWrimK* Mutant Strains.

LC-MS/MS chromatograms utilizing multiple reaction monitoring (MRM) searching for the presence of *iso*-maleimycin **2** in various samples: *Streptomyces* sp. QL37 wild-type (WT), Δ*minPKS* and Δ*lysWrimK* mutant. Notably, the *iso*-maleimycin MeOH adduct peak, detected at a retention time of 1.25 min, is observed in the wild-type strain and *ΔminPKS* but is absent in the Δ*lysWrimK* strain. A sample of extract from not inoculated minimal medium was included as a control (MM blank).

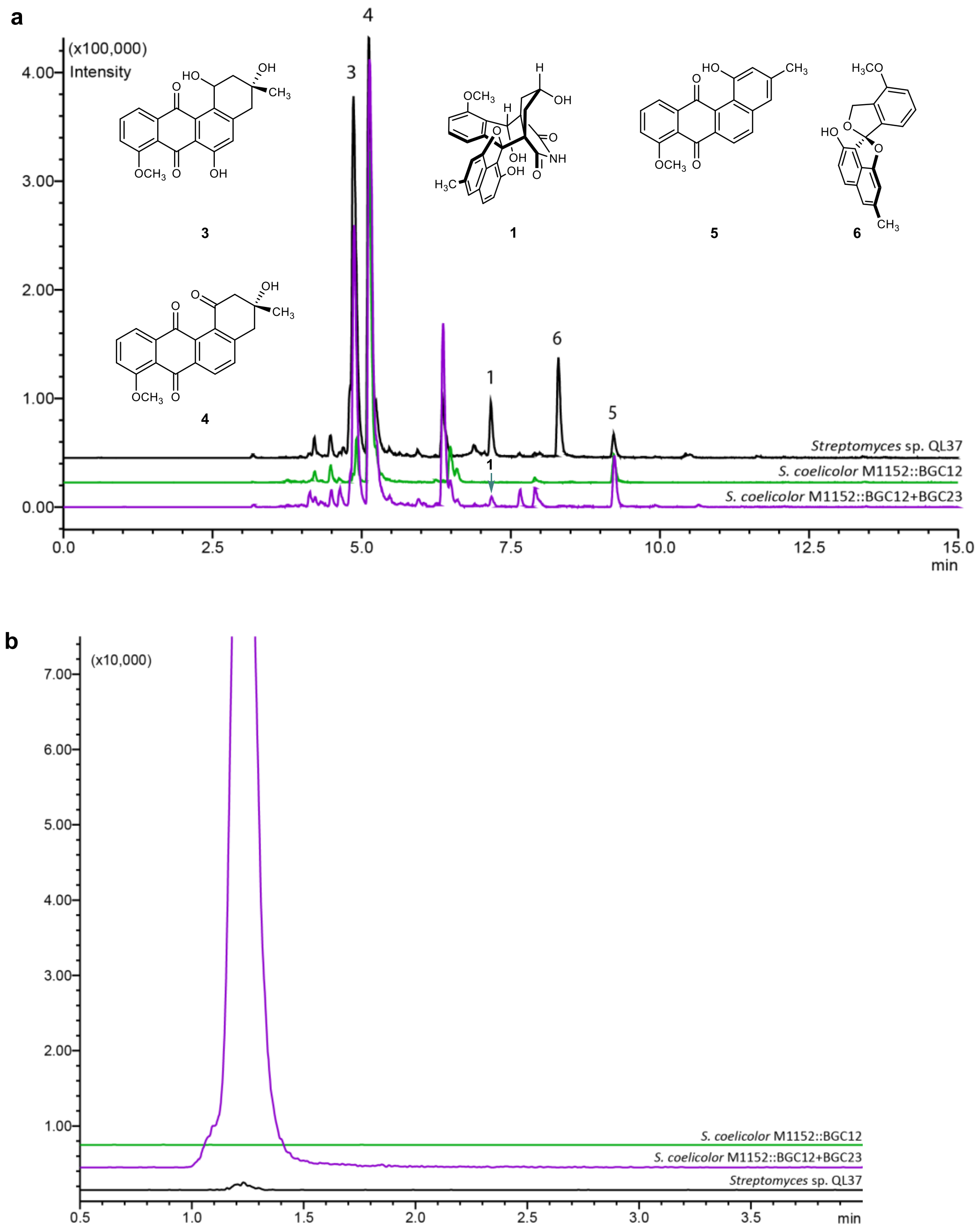

Figure S4. Comparative LC-MS chromatograms of *Streptomyces* sp. QL37 and of *S.coelicolor* M1152 with either BGC12 or BGC12+BGC23

(a) Extracted ion chromatograms (XIC) for angucyclinones and lugdunomycin produced by control strain *Streptomyces* sp. QL37, corresponding to the following m/z values: 456.144 (**1**), 355.117 (**3**), 339.123 (**4**), 319.096 (**5**), and 321.111 (**6**), with a tolerance of 20 ppm. *S. coelicolor* M1152 containing BGC12 (M1152::BGC12) produces angucyclines but not lugdunomycin **1**. Production of **1** (see arrow) was seen when both BGCs were expressed heterologously in *S. coelicolor* M1152 (*S. coelicolor* M1152::BGC12+BGC23). Note that **6** is not detected in *S. coelicolor* strains, possibly due to toxicity to the heterologous host. **(b)** LC-MS/MS chromatograms using multiple reaction monitoring (MRM) to detect iso-maleimycin. Heterologous strains containing BGC23 show a significant peak of iso-maleimycin.

a

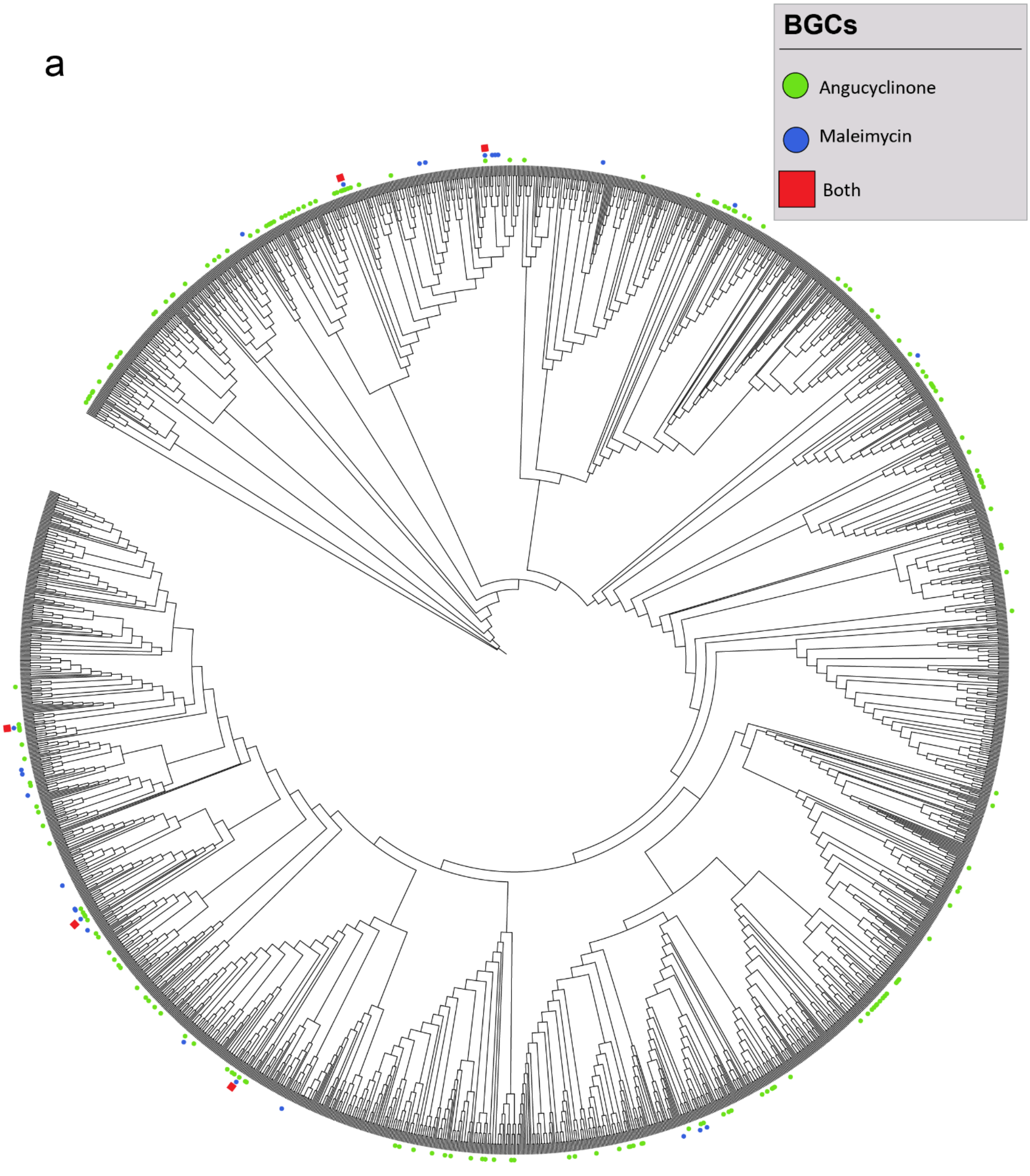

b

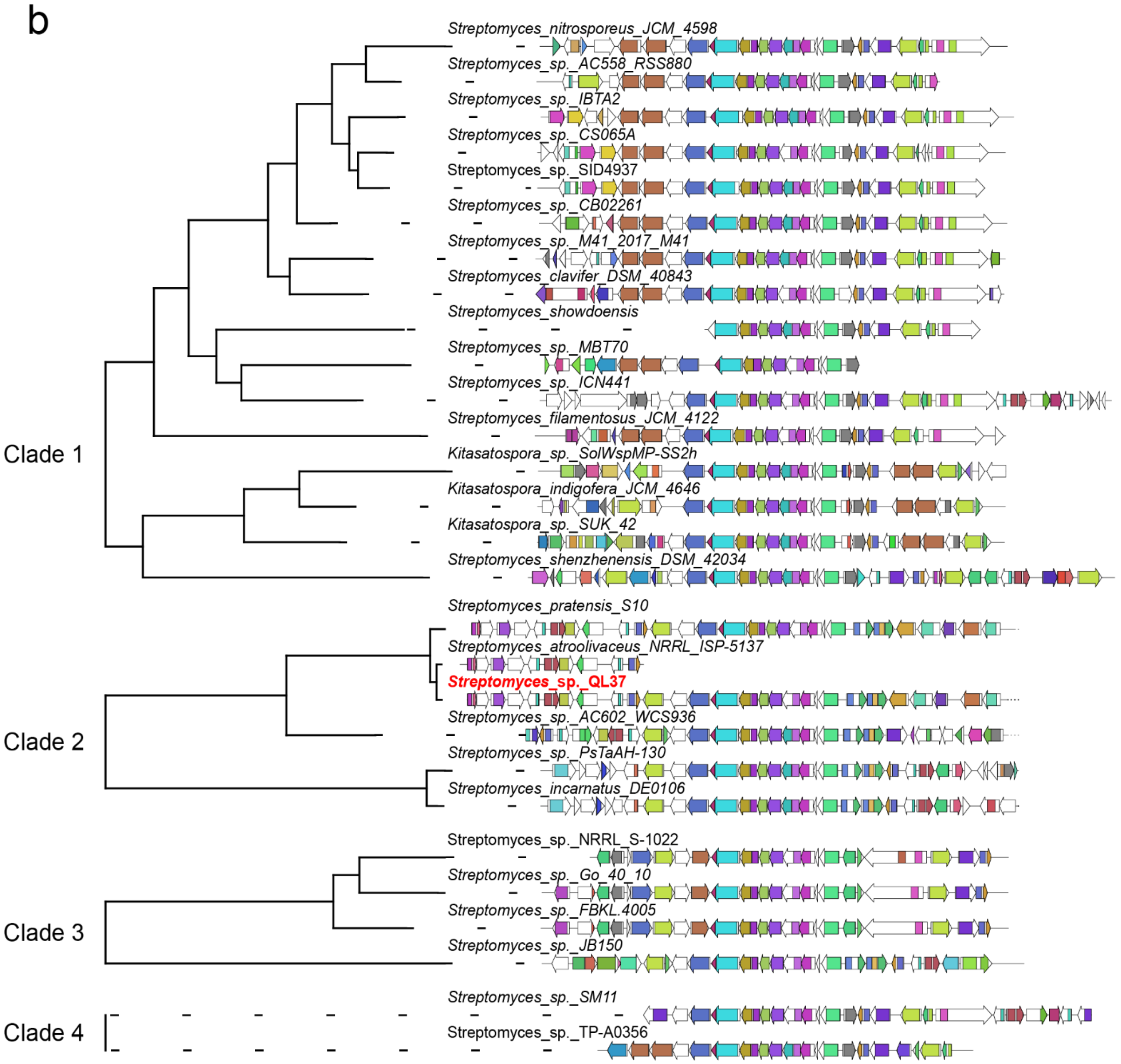

Figure S5. Bioinformatic analysis of *Streptomyces* and *Kitasatospora* genomes identifying BGCs for angucyclines and for *iso*-maleimycin-like molecules**. a**. Phylogenetic tree of strains predicted to contain angucycline BGCs (similar to BGC12) and/or BGCs similar to the BGC for maleimycin-like molecules (BGC23a). The phylogenetic tree depicts the relationship among *Streptomyces* and *Kitasatospora* strains analyzed using PhyloPhlAn (version 3.0.60)[22]. Strains containing likely angucycline BGCs are highlighted in green, while those likely to contain BGCs similar to the BGC for maleimycin-like molecules are highlighted in blue. Strains harbouring both types of BGCs are denoted in red. Note that co-occurrence of the two different BGCs is infrequent, with five co-occurrences found in our analysis. This co-occurrence is not related to phylogeny. **b**. Alignment of BGCs similar to the BGC (based on BiG-SCAPE) for maleimycin-like molecules in the analyzed genomes. Four distinct types of BGCs similar to the *iso*-maleimycin BGC23a were grouped by BiG-SCAPE, characterized by the conservation of core genes and their arrangement within the clusters.

Table S1: Differential protein expression between galactose- and glucose-grown MM cultures of *Streptomyces* sp. QL37.

**Proteins annotation #**	**log2FC ^**
**QL37_30735 GMP synthase [glutamine-hydrolyzing]**	**6.0218079**
QL37_36965 ABC transporter periplasmic-binding protein YtfQ	5.2994111
QL37_08055 hypothetical protein	4.6876532
QL37_15965 Galactokinase	4.4700264
QL37_11300 Multiple sugar-binding periplasmic protein SbpA	4.4042577
QL37_11810 Pyruvate, phosphate dikinase	4.3336984
**QL37_30710 2-isopropylmalate synthase**	**4.2639892**
QL37_07245 hypothetical protein	4.1272887
QL37_15975 Galactose-1-phosphate uridylyltransferase	4.0765178
**QL37_30755 hypothetical protein**	**3.9478366**
**QL37_30705 Acyl-CoA dehydrogenase**	**3.6859316**
QL37_07695 hypothetical protein	3.4194662
QL37_09770 Transcriptional regulator SlyA	3.1588078
**QL37_30745 Short-chain-fatty-acid--CoA ligase**	**2.6588961**
QL37_08755 Putative prophage major tail sheath protein	2.5847662
QL37_07175 Benzaldehyde dehydrogenase [NAD(+)]	2.5527653
**QL37_30750 Aspartate aminotransferase**	**2.5430627**
QL37_09775 hypothetical protein	2.5100967
QL37_19275 Acireductone dioxygenase	2.4473463
**QL37_30720 Alpha-aminoadipate--LysW ligase LysX**	**2.2967689**
QL37_03115 hypothetical protein	2.2783263
QL37_32225 Glycerol kinase	2.2180443
QL37_20690 Daunorubicin/doxorubicin resistance ATP-binding protein DrrA	2.1672179
QL37_36990 Aldose 1-epimerase	2.1152377
QL37_25910 ATP synthase subunit a	2.0875887
QL37_25595 hypothetical protein	2.044777
QL37_25085 Succinyl-diaminopimelate desuccinylase	-2.90E-05
QL37_17640 hypothetical protein	-2.067939
QL37_20340 Putative oxidoreductase YdbC	-2.087165
QL37_29760 hypothetical protein	-2.18598
QL37_24510 hypothetical protein	-2.231756
QL37_33135 putative peptidyl-prolyl cis-trans isomerase B	-2.285745
QL37_26270 hypothetical protein	-2.553681
QL37_12020 30S ribosomal protein S20	-2.566372
QL37_10455 Sugar phosphatase YfbT	-2.666196
QL37_22665 30S ribosomal protein S12	-2.728723
QL37_19985 hypothetical protein	-2.79044
QL37_27255 D-xylose-proton symporter	-3.395676

In bold, the proteins belonging to the predicted BGC23 by antiSMASH.

^ Positive fold change indicate upregulation in galactose and negative fold change indicates upregulation in glucose (n=4).

Table S2: Primer Sequences for knock-out constructs and verifications of mutants ("check") in BGC23 of *Streptomyces* sp. QL37.

**Primer name**	**Sequence**
LysWRimK_check_R2	CCGACGATTCCTGCTCGGATC
LysWRimK_LF_FW2	CTAGGAATTCGCCGATTTATCGGACGAATG
LysWRimK_LF_RV2	CTAGTCTAGACGGCTCAGACGTTCTGGAAG
LysWRimK_RF_FW	CTAGTCTAGATCCACGACCGACACAGACATC
LysWRimK_RF_RV	CTAGAAGCTTCGACGAGCTGCTTGTCCTTC
WK_check_F	CGAACTCGTCGGCAACACACG
BGC23_P1	CAGGAAACAGCTATGACATGATTACGAATTCGATATCGTTCGGGCTGTGCGAGCACCA
BGC23_P2	CAGGTTCGCGTGATGGCGGTTCC
BGC23_P3	GGAACCGCCATCACGCGAACCTG
BGC23_P4	GTGCTACGGGCGGTTTCGGAGG
BGC23_P5	CCTCCGAAACCGCCCGTAGCAC
BGC23_P6	GCTTGGGCTGCAGGTCGACTCTAGAGCTGGGACGGAGGCATGGAGAC
garL-T0_REV	TTGGGCTGCAGGTCGACTCTAGATGGACTCACAAAGAAAAAACGCCCGGTGTG CAAGACCGAGCGTTCTGAACAATCAGACGGGGGCGTCGGCCGC
garL_FOR	ACTCCACAGGAGGACCCATGTGACCGCGCACCGCACACC
seq_F_permE::GarL	GGGCTGCAGGTCGACTCT
seq_R_permE::GarL	ACTCATTAGGCACCCCAGGC
P1_FR1_*garL*	AGCTGAATTCGCGCTCTTCCTCGGCGAG
P2_FR1_*garL*	GAAGTTATCCATCACCTCTAGA CTGCTTGAGGCCGCGGCG
P3_FR2_*garL*	GAAGTTATCGCGCATCTCTAGA CCGGGTCCGGGACGGCCG
P4_FR2_*garL*	GCCAAGCTTCGAGAGGATCGCGAGCATG

Section 2: Computational experiments of GarL as a possible Diels-Alderase in *Streptomyces* sp. QL37

We set out to see if we could identify a possible gene for a Diels-Alderase in *Streptomyces* sp. QL37. Bioinformatics analysis of the genes in BGC12 and BGC23a failed to identify a candidate gene for a Diels-Alderase. We then used macrophomate synthase (MPS)[23, 24] as input in a search of the predicted gene products of *Streptomyces* QL37. MPS is found in *Macrophoma commelinae*, a fungus isolated from spots on the leaves of *Commelina communis* and although identified as a Diels-Alderase [23], was later shown to catalyse a stepwise Michael–aldol reaction [24, 25]. Interestingly, this retrieved a single hit with significant homology to MPS (25% amino acid identity and 41% similarity), namely an enzyme annotated as 5-keto-4-deoxy-D-glucarate aldolase (GarL). GarL is involved in carbohydrate metabolism, and specifically in galactarate degradation [26]. The fact that both MPS and GarL are aldolases provided additional support for the notion that GarL candidated as a template for the Diels-Alder reaction.

To assess whether GarL could indeed facilitate the reaction between isobenzofuran and *iso*-maleimycin, AlphaFold v3.0 [27] was used to generate an initial conformation of the GarL and MPS proteins and their active sites (Figure S6A). The stability of the secondary structure of GarL was evaluated using the root-mean-square fluctuation (RMSF), which was converted into a B-factor as an order parameter. The results were compared to those obtained with the active site of MPS (PDB 1ICZ)[23]. This revealed that the predicted active sites of both proteins are very similar.

Molecular Dynamics (MD) simulations using GROMACS were then conducted to evaluate the stability of *iso*-maleimycin and the isobenzofuran formed from elmonin, within the active site of GarL. To further assess the behavior and stability of this docked complex, MD simulations were performed over a 50 ns timescale. These simulations revealed that the complex remained stable within the cavity throughout the entire simulation period, with no significant deviations in the protein-ligand interactions or structural integrity of the binding site. This suggest that the active site is functional, like in MPS. These observations led us to conclude that GarL has an active site very similar to MPS, and that this active site accommodates both the diene and the dienophile for the production of lugdunomycin.

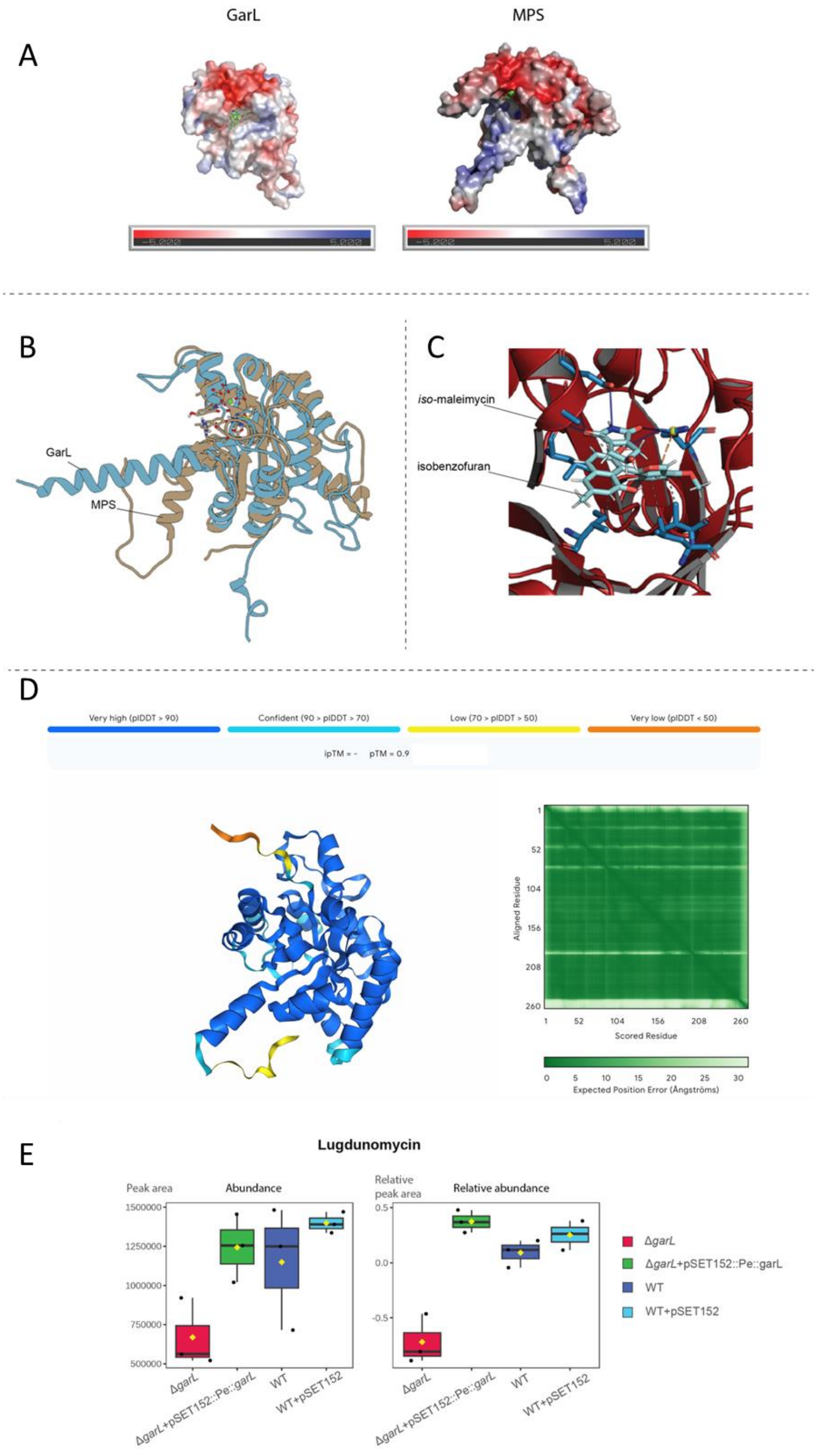
A
GarL
MPS
-5.000
5.000
B
GarL
MPS
C
iso-maleimycin
isobenzofuran
D
Very high (pIDDT > 90)
Confident (90 > pIDDT > 70)
Low (70 > pIDDT > 50)
Very low (pIDDT < 50)
ipTM = - pTM = 0.9
Aligned Residue
Scored Residue
1
52
104
156
208
260
0 5 10 15 20 25 30
Expected Position Error (Ångströms)
E
Lugdunomycin
Peak area
Abundance
1500000
1250000
1000000
750000
500000
Relative peak area
Relative abundance
0.5
0.0
-0.5
ΔgarL
ΔgarL+pSET152::Pe::garL
WT
WT+pSET152

Figure S6. **Experimental and modelling results about the possible role of GarL in lugdunomycin biosynthesis in *Streptomyces* sp. QL37. A)** Electrostatic potential of GarL and MPS (PDBid 1IZC). **B)** Superposition of the GarL structure from the MD simulation in blue, and the structure from 1IZC in beige. The residues from the active site are represented in licorice representation. **C)** *Iso*-maleimycin (the dienophile in the Diels-Alder reaction) and the isobenzofuran formed from elmonin (the diene) docked in the active site of GarL. 3D representation of the active site of GarL protein with the different interacting residues. The hydrophophic interactions are represented in solid blue line, the hydrophobic interactions in a dash gray line, and the Pi stacking interactions in an orange dash line. **D)** Confidence score of the structure predicted by AlphaFold. **E)** ANOVA statistical analysis of the peak area values integrated from LC-MS measurements following TIC sum normalization, log transformation and Pareto scaling. The differential production of lugdunomycin in *Streptomyces* sp. QL37 (WT), its *garL* knock-out mutant (Δ*garL*), mutant Δ*garL* complemented by expression of *garL* (Δ*garL*+pSET152::Pe::*garL*) and the parental strain QL37 containing the empty vector (WT::pSET152) is illustrated.

A *garL* null mutant of *Streptomyces* sp. QL37 shows reduced lugdunomycin production

To further investigate the role of *garL* in lugdunomycin biosynthesis, a gene deletion mutant was generated and the effect on lugdunomycin production analysed using metabolomics. For this, the *garL* gene was replaced by the apramycin resistance cassette (*aacC4*) through homologous recombination, generating *Streptomyces* sp. QL37 Δ*garL*. To ensure that indeed the phenotypes were due to the deletion of *garL*, the mutant was genetically complemented by introducing a construct wherein *garL* is expressed from the consitutive *ermE* promoter (Δ*garL*+pSET152::Pe::*garL*), whereby a strain containing only the empty vector (Δ*garL*+pSET152) was used as a control. The strains were cultivated for 7 days on MM agar supplemented with 0.5% mannitol and 1% glycerol. Ethyl acetate extracts were prepared, and LC-MS analysis was performed to measure the metabolite profiles of the extracts. Chromatographic peak areas were integrated from the LC-MS data and used for quantification. Following data acquisition, the peak area values were processed using normalization by sum, followed by log-transformation and Pareto scaling to facilitate multivariate analysis and comparison of relative metabolite. Data processing was performed using MetaboAnalyst. Comparative metabolomics analysis of the strains using LC-MS revealed that the extracts from the wild-type and the complemented strain contained significantly higher concentrations of lugdunomycin compared to the *garL* knock-out mutant (ANOVA, $p < 0.05$) (Figure S6E). The relative titer of lugdunomycin in the extracts after the normalization by sum of the values of *ΔgarL*, the complemented strain and the WT complemented with the empty vector pSET152 were calculated for

each strain, with values reported relative to the WT strain. The relative titer values were as follows: Δ*garL*: 0.43, complemented strain: 1.34, and WT complemented with the empty vector pSET152 1.19. This indicates that deletion of *garL* leads to a significant reduction in lugdunomycin biosynthesis as compared to the WT strain.

Molecular Dynamics Simulations.

AlphaFold v3.0[27] was used to obtain a starting configuration of the GarL sequence that was expressed experimentally. The active site was determined by using the activesite prediction server, which predicted 32 possible cavities, which were compared with a similar cavity of a Diels Alderase[23] (pdb: 1ICZ) reported by Guimaraes et al.[24] .The reactants (isobenzofuran and iso-maleimycin) were docked into the active site of the lowest energy conformation of GARL, for which AutoDockVina[28] was used.

Molecular dynamics simulations were performed on the protein ligand complex to assess the stability of the reactants in the active site of GarL.

The molecular dynamics simulations were done using GROMACS[29] 2024. The Amber19SB[30] was used in all simulations. The protein systems were solvated with the OPC water model[31] , and sufficient sodium/chlorine counterions were added for a neutral simulation box.

After a first energy minimization, two 100 ps equilibrations were performed: an NVT equilibration at a temperature of 300.15 K[32] followed by an NPT equilibration at a pressure of 1 bar using a Parrinello−Rahman barostat[33]. During the equilibration runs, all bonds were constrained using the LINCS algorithm. A 2 fs time step was used for all MD runs. For both Lennard-Jones and Coulomb interactions[34] a 1.0 nm cutoff was used. The long-range Coulomb interactions were accounted for using the particle mesh Ewald (PME) method[35] a grid step of 0.16 nm and a convergence of 10^{-5} were chosen. Analytic corrections to the pressure and potential energy[34] were introduced to compensate for the truncation of the Lennard-Jones interactions. The temperature was kept constant by using a V-rescale thermostat[32] with an inverse time constant of τ^{-1} = 0.2 ps^{-1}. The position restraints were set to 0 for the protein-ligand complex during the 50 ns production run.

Analysis.

The analysis of the protein-ligand complex was performed using the LigPlot+ software suite[36]. The secondary structure stability was analyzed using the dssp tool[37] available in GROMACS, and the free energy surface was analyzed using the radii of gyration (Rg) and the root mean square deviation (RMSD)

as order parameters. The fluctuation of the secondary structure was evaluated with the root mean square fluctuation (RMSF) metrics, using the rmsf tool available in GROMACS.

Section 3: Synthetic experimental procedures

General

The precursors for the synthesis of lugdunomycin, compounds **2** and **6** were obtained by chemical synthesis as previously reported in the literature[7, 8]. Reactions above rt were heated using an IKA stir plate provided with an Asynt drysyn aluminium heating block. All reaction solvents were purchased from commercial vendors and used without further purification unless specified otherwise. Reagents were purchased from chemical vendors and used without further treatment or purification, unless stated otherwise. NMR spectra were recorded on an Agilent 400 NMR spectrometer, or on a Bruker 600 MHz NMR spectrometer in the solvent as described specifically for each compound. Detected ^{1}H-nuclei at 400 MHz or 600 MHz, ^{13}C-nuclei at 101 MHz or 151 MHz. Reported chemical shifts are given in ppm, relative to the residual solvent signal. Analytical TLC plates (60/Kieselguhr F254, 0.25 mm), provided with a fluorescent marker, were obtained from Merck Chemicals. Spots were visualised by means of a UV lamp or appropriate standard staining solutions; KMnO4, Anisaldehyde, Seebach's stain or phosphomolybdic acid. HRMS was executed on a Thermo-Fisher Orbitrap Electron Spray Ionization (ESI) mass spectrometer at positive ionization mode, unless specified differently.

Preparation of lugdunomycin derivatives

9-*epi*-lugdunomycin 12

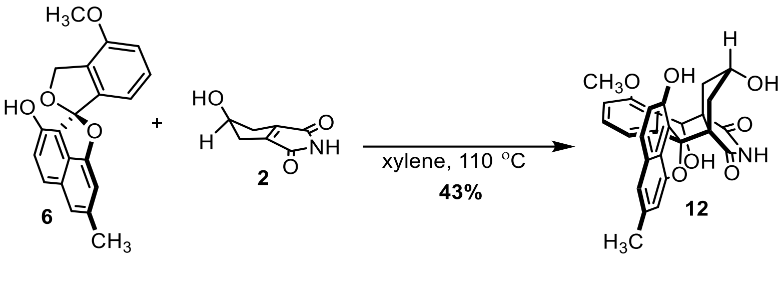

In a 2 mL vial, a stir bar, elmonin **6** (15.8 mg, 49.3 µmol) and *iso*-maleimycin **2** (15.8 mg, 103 µmol) were placed, and suspended in 100 µL *m*-xylene. The mixture was heated to 110 °C for 20 h, after which a white precipitate was observed and full conversion was confirmed by means of TLC.* The mixture was allowed to cool to rt, the xylene was carefully removed by rotary evaporation and the mixture was re-dissolved in

MeOH and loaded onto celite to be chromatographed on silica (gradient, MeOH/DCM, 7.5:92.5 and 8:92), providing 9-*epi*-lugdunomycin **12** (10.1 mg, 21.3 µmol, 43%) as a white solid. Single crystals for X-ray diffraction analysis were obtained using vapour diffusion crystallisation, by slowly diffusing toluene into a solution of the compound in MeOH.

^{1}H-NMR (600 MHz, D_3COD) δ 7.62 (d, *J* = 8.6 Hz, 1H), 7.27 (t, *J* = 8.1 Hz, 1H), 7.21 (d, *J* = 8.6 Hz, 1H), 7.13 (dd, *J* = 8.4, 1.0 Hz, 1H), 7.04 (s, 1H), 6.75 (dd, *J* = 8.0, 1.0 Hz, 1H), 6.38 (d, *J* = 0.9 Hz, 1H), 5.55 (s, 1H), 4.07 (dd, *J* = 3.5, 1.8 Hz, 1H), 3.93 (s, 3H), 2.48 (dd, *J* = 14.4, 12.5 Hz, 1H), 2.43 – 2.35 (m, 2H), 2.37 (s, 3H), 1.39 (dd, J = 13.7, 3.6 Hz, 1H).

13C-NMR (151 MHz, D3COD) δ 181.8, 180.0, 158.1, 156.2, 148.8, 135.6, 134.0, 129.6, 129.4, 126.4, 126.1, 124.9, 120.9, 120.8, 115.3, 112.3, 102.3, 98.0, 70.1, 63.5, 62.9, 60.5, 55.2, 45.0, 43.9, 21.1.

HRMS calcd for $C_{27}H_{23}NO_7$ $\{M+Na\}^+$: 496.1367 found: 496.1342.

Note:

[*] 9-*epi*-lugdunomycin **12** can be easily recognised by TLC analysis as it gives an intense blue spot after staining with anisaldehyde solution[14].

Lugdunomycin 1

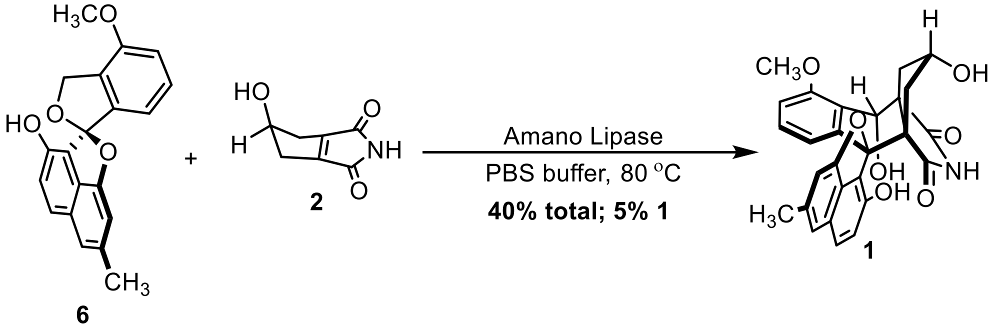

A 20 mL screwcap vial was loaded with a stir bar, elmonin **6** (125 mg, 390 µmol) and *iso*-maleimycin **2** (220 mg, 1.43 mmol). The solids were carefully dissolved in DMSO (3.8 mL), to which a solution of amano lipase (1.27 g) in standard PBS[a] (8.9 mL) was added. The solution was stirred at 80 °C for 24 h, at which point TLC indicated full consumption of 6.[b] The mixture was extracted with EtOAc (4 × 35 mL) and the combined organic layers were washed with brine (2 × 30 mL) to remove any DMSO, then dried over $MgSO_4$, and

concentrated by means of rotary evaporation. The crude mixture (164 mg) was loaded onto silica and purified by column chromatography (MeOH/DCM, gradient 4:96 and 5:95) to give an inseparable diastereomeric product mixture[c] (73.8 mg, 156 µmol, 40%) as a colourless glassy solid. The mixture was re-dissolved in MeOH/MeCN (1:1, 18 mL), pressed through a MN Chromafil Xtra R45/13 0.45 µm filter to remove any solid particles and separation of the diastereomers was performed in multiple portions on a Shimadzu LC-20AP preparative HPLC system, provided with an Axia Kinetex© 5 µm C18 100 Å (250 mm L × 30 mm Ø) LC column coupled to a Shimadzu Prominence UV detector, followed by a FRC-10A automatic fraction collector. Pump flow 42.53 mL/min, solvent A; H_2O, solvent B; MeCN, gradient 5% B to 71.5% B in 14 minutes, then 5% B hold for 6 minutes, injection volume 1000 µL, UV detection at λ 254 and 210 nm. Combining of the collected fractions afforded lugdunomycin **1** (8.4 mg, 18 µmol, 5%) as a colourless solid.

^{1}H-NMR (600 MHz, D_3COD) δ 7.42 (d, *J* = 8.5 Hz, 1H), 7.24 (t, *J* = 8.2 Hz, 1H), 6.98 (d, *J* = 7.5 Hz, 1H), 6.98 (s, 1H), 6.93 (dd, *J* = 7.9, 0.9 Hz, 1H), 6.80 (d, *J* = 8.5 Hz, 1H), 6.58 (d, *J* = 1.0 Hz, 1H), 5.65 (s, 1H), 4.10 (t, *J* = 3.6 Hz, 1H), 3.91 (s, 3H), 2.69 – 2.59 (m, 2H), 2.47 (s, 3H), 2.41 (dd, *J* = 14.5, 3.7 Hz, 1H), 1.63 (dd, *J* = 13.6, 3.7 Hz, 1H).

^{13}C-NMR (151 MHz, D_3COD) δ 182.8, 182.7, 159.27, 158.8, 148.4, 139.6, 136.8, 131.3, 129.5, 127.9, 127.5, 124.1, 123.5, 122.7, 120.1, 116.1, 111.5, 102.8, 95.2, 70.8, 62.9, 62.7, 60.1, 56.5, 48.3, 47.8, 22.5.

HRMS calcd for $C_{27}H_{23}O_7$ $\{M+H\}^+$: 474.1544 found: 474.1531.
Spectral data matched with those obtained from the natural material[14].

Notes:

[a]Phosphate Buffer Saline, standard phosphate buffer, without the addition of other salts, pH 7.

[b]Lugdunomycin and its isomers can be easily recognised by TLC analysis as they give intense blue spots after staining with anisaldehyde solution[14].

[c]Based on inspection by ^{1}H-NMR, the number of signals corresponding to H17 are indicative for the number of diastereomers.

Comparative NMR table of synthetic lugdunomycin 1

Table S3: Comparative NMR table of synthetic and natural lugdunomycin displaying ^{1}H and ^{13}C chemical shifts of natural lugdunomycin and synthetically prepared lugdunomycin.

	Natural[a]		Synthetic[a]	
Position	δ_H (mult.)	δ_C	δ_H (mult.)	δ_C ($\Delta\delta$)
1	-	159.2	-	159.3 (+0.1)
2	6.96 (s)[b]	116.1	6.98 (s)[b]	116.1 (±0.0)
3	-	136.6	-	136.8 (+0.2)
4	6.57 (s)	102.8	6.58 (s)	102.8 (±0.0)
4a	-	127.4	-	127.5 (+0.1)
5	6.79 (d)	127.8	6.80 (d)	127.9 (+0.1)
6	7.40 (s)	124.3	7.42 (s)	124.1 (-0.2)
7	-	148.6	-	148.4 (-0.2)
8	-	122.7	-	122.7 (±0.0)
8a	-	129.5	-	129.5 (±0.0)
9	-	95.2	-	95.2 (±0.0)
10	-	139.6	-	139.6 (±0.0)
11	6.96 (d)[b]	111.5	6.98 (d)[b]	111.5 (±0.0)
12	7.23 (t)	131.3	7.24 (t)	131.3 (±0.0)
13	6.93 (d)	120.1	6.93 (dd)	120.1 (±0.0)
14	-	158.8	-	158.8 (±0.0)
15	-	123.5	-	123.5 (±0.0)
16	5.63 (s)	62.9	5.65 (s)	62.9 (±0.0)
17	-	62.7	-	62.7 (±0.0)
18	2.41 (dd), 2.65 (m)	47.7	2.41 (dd), 2.64 (m)	47.7 (±0.0)
19	4.10 (t)	70.8	4.10 (t)	70.8 (±0.0)
20	1.63 (dd), 2.65 (m)	48.3	1.63 (dd), 2.64 (m)	48.3 (±0.0)
21	-	60.1	-	60.1 (±0.0)
22	-	182.8	-	182.7 (-0.1)
24	-	182.9	-	182.8 (-0.1)
25	2.47 (s)	22.5	2.47 (s)	22.5 (±0.0)
26	3.91 (s)	56.5	3.91 (s)	56.5 (±0.0)

[a]Chemical shifts in ppm, relative to the residual solvent signal. Spectrum recorded in D_3COD. ^{1}H resonances recorded at 600 MHz, ^{13}C at 151 MHz. [b]Signals overlap but are clearly distinguishable.

Lugdunomycin derivative 14

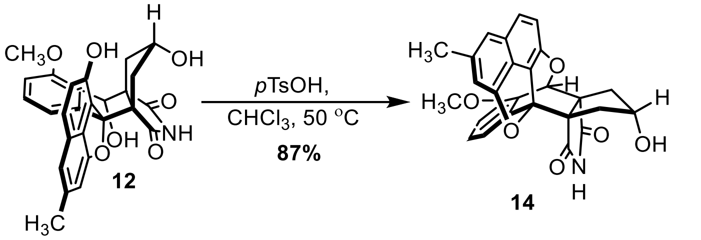

To a 4 mL vial provided with a stir bar and 9-*epi*-lugdunomycin **12** (2.1 mg, 4.4 µmol), chloroform was added, followed by one crystal of *p*TsOH. The vial was heated to 50 °C for 48 h, the solvent was evaporated by means of rotary evaporation and the material was loaded onto celite to be chromatographed on silica (DCM/MeOH, 95:5), affording the cyclised *bis*-ether **14** (1.7 mg, 3.9 µmol, 87%) as a colourless solid.

^{1}H-NMR (600 MHz, D_3COD) δ 7.48 (d, *J* = 8.7 Hz, 1H), 7.34 (t, *J* = 8.0 Hz, 1H), 7.04 (d, *J* = 7.7 Hz, 2H), 7.02 (s, 1H), 7.00 (d, *J* = 8.3 Hz, 1H), 6.92 (d, *J* = 8.7 Hz, 1H), 6.73 (s, 1H), 5.95 (s, 1H), 4.27 (t, *J* = 3.6 Hz, 1H), 3.87 (s, 3H), 2.46 (s, 3H), 2.38 (dd, *J* = 13.9, 2.1 Hz, 1H), 2.32 (dd, *J* = 14.1, 3.6 Hz, 1H), 2.27 (dd, *J* = 14.2, 2.4 Hz, 1H), 1.74 (dd, *J* = 14.2, 3.8 Hz, 1H).

^{13}C-NMR (151 MHz, D_3COD) δ 182.4, 181.6, 161.2, 158.7, 145.4, 145.3, 139.5, 132.8, 127.7, 127.6, 126.6, 124.0, 120.7, 118.8, 116.8, 113.6, 112.1, 105.5, 94.3, 74.9, 73.5, 67.5, 65.9, 56.6, 49.6, 41.0, 39.9, 22.9.

HRMS calcd for $C_{27}H_{21}NO_6$ $\{M+H\}^+$: 456.1442 found: 456.1436

Analytical scale optimisation of lugdunomycin synthesis

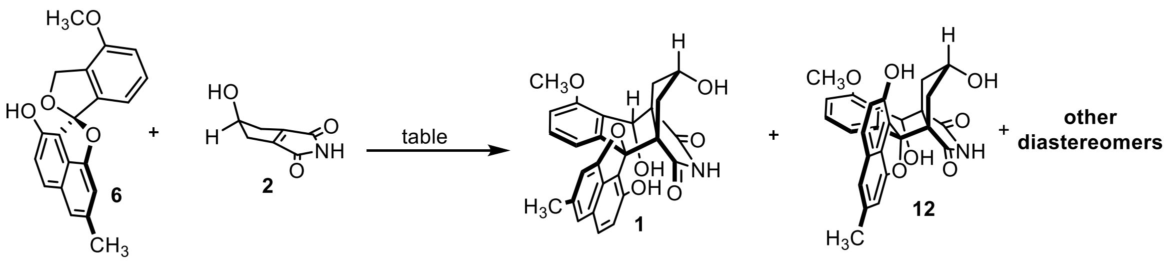

A 2 mL vial was provided with a stir bar, elmonin **6** (1 mg, 3 µmol) and *iso*-maleimycin **2** (2 mg, 13 µmol). The contents were dissolved in the appropriate co-solvent (5 – 35 v/v %, see table) and then water or PBS was added to a final reaction volume of 200 µL. The vial was closed with a screwcap with rubber insert and the mixture was placed in a heating block to be heated at the desired temperature for 24 h – 4 d (see table). The mixture was allowed to cool to rt, EtOAc (1.5 mL) was added and the mixture was shaken for 10 sec, the organic layer was separated with a Pasteur pipette and the procedure was repeated (2 × 1.5 mL). The combined organic layers were washed by mixing with brine (10 mL) in a 20 mL screwcap vial and shaking for 10 sec before removing the aqueous layer with a Pasteur pipette. The organic layer was dried by filtration over a $MgSO_4$ plug, the solvent was evaporated by means of rotary evaporation. The residue was redissolved in MeCN (2.00 mL) and then pressed through a MN Chromafil Xtra R45/13 0.45 µm filter and analysed on a Thermo Scientific Vanquish UHPLC system provided with a UV/VIS detector, coupled to a Thermo Scientific LCQ Fleet ESI Mass spectrometer. Separation was carried out on a HSS T3 column (150 mm L × 2.1 mm Ø, 1.8 µm). Column temperature 30 °C, flow rate 0.3 mL/min, solvent A; 0.1 v/v % HCO_2H in H_2O, solvent B; 0.1 v/v % HCO_2H in MeCN, gradient 5% B hold for 1 min, then 5% – 85% B in 9

min, then 85% – 100% B for 1 min and finally hold 100% B for 3 min, the column was equilibrated at 5% B for 5 min before the next run. Ionisation mode positive ESI, scanned mass range m/z 50 – 700. Integration of the peaks corresponding to either **1**, **12** or the other diastereomers from the extracted ion chromatogram with mass ranges m/z 496-497, 474-475, 456-457 was done to determine the d.r.

Table S4: Optimization of lugdunomycin formation, diastereoselectivity.

Entry	Solvent	T [°C]	Additive	d.r. 1/12/other (isolated yield)
1[a]	water	45	-	0:2:1
2[a]	water	90	-	1:8:2
3[a]	water	45	SDS, 0.015 M	1:9:3
4[a]	water	45	SDS, 0.1 M	1:14:1
5[a]	water/DMSO 5%	37	PBS, pH 7	1:8:4
6[a]	water/DMSO 5%	37	PBS, pH 7, BSA	1:5:2
7[a]	water/DMSO 30%	37	PBS, pH 7, BSA	1:3:2
8[a,b]	water/glycerol 35%	37	PBS, pH 7, BSA	n.d.
9[a]	water/EtOH 35%	37	PBS, pH 7, BSA	1:3:1
10[a]	water/MeCN 35%	37	PBS, pH 7, BSA	1:2:1
11[a]	water/DMSO 30%	80	PBS, pH 7	2:6:1
12[a,c,d]	water/DMSO 30%	80	PBS, pH 7, Amano lipase	2:2:1 (40% total, 5% **1**)

[a]Analytical scale reactions performed using 13 µmol (4 equiv) **2** and 3 µmol **6** at 15.6 mM and stirring for four days, remaining starting material **6** was always observed. Products were identified using LC-MS, integration of the peaks corresponding to either **1**, **12** or the other diastereomers from the extracted ion chromatogram with mass range m/z 496-497, 474-475, 456-457 was done to determine the d.r. It is assumed that each diastereomer has roughly the same ionisation efficiency and therefore the relative peak areas are assumed to be approximately proportional to the relative concentration. PBS = Phosphate Buffered Saline. [b]Concentration of products was too low to draw conclusions. [c]Amano lipase from *Pseudomonas fluorescens*. Reaction completed within 24 h. [d]Reaction repeated as a preparative scale experiment using 1.43 mmol (3.6 equiv) **3** and 390 µmol **6**, stirring for 24 h, erosion of d.r. observed during scale-up. Total isolated yield of lugdunomycin diastereomers 40%, compound **1** was separated from the diastereomeric mixture using preparative HPLC.

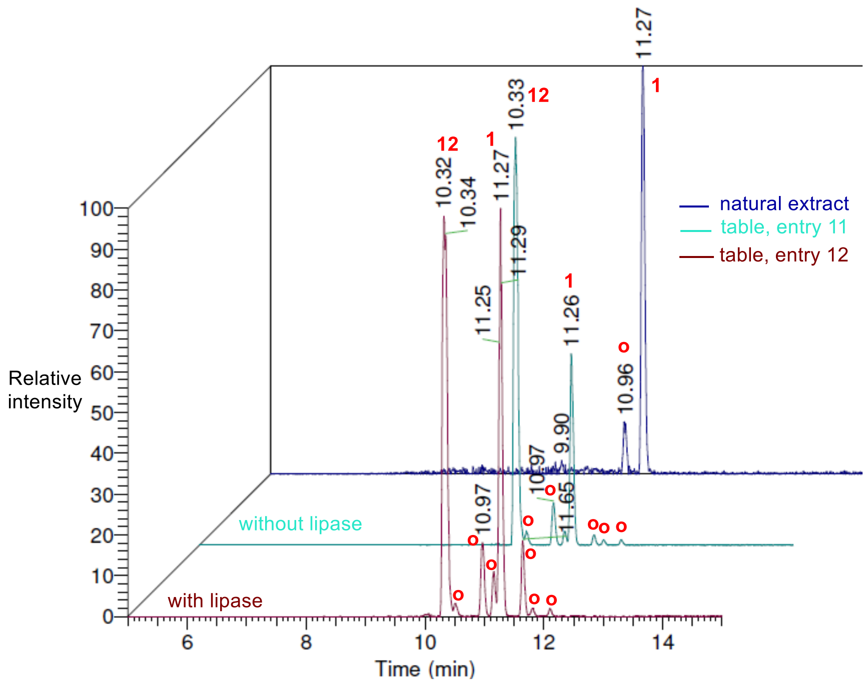

Example of extracted ion chromatograms (mass ranges m/z 496-497, 474-475, 456-457) of analytical scale reactions between **6** and **2**, with (Table S4 entry 12) and without the addition of Amano lipase (Table S4, entry 11), and of a

natural extract of *Streptomyces* sp. QL37 for comparison. It is assumed that each diastereomer has approximately the same ionisation constant and therefore the relative peak ratios are assumed to be directly proportional to the relative concentrations of each diastereomer. The descriptor "**o**" above the remaining peaks refers to other presumed diastereomers with unknown structure.

Crystal data of compound 12

Crystal data have been submitted to the Cambridge Crystallographic Data Center (CCDC) under deposition number 2215151.

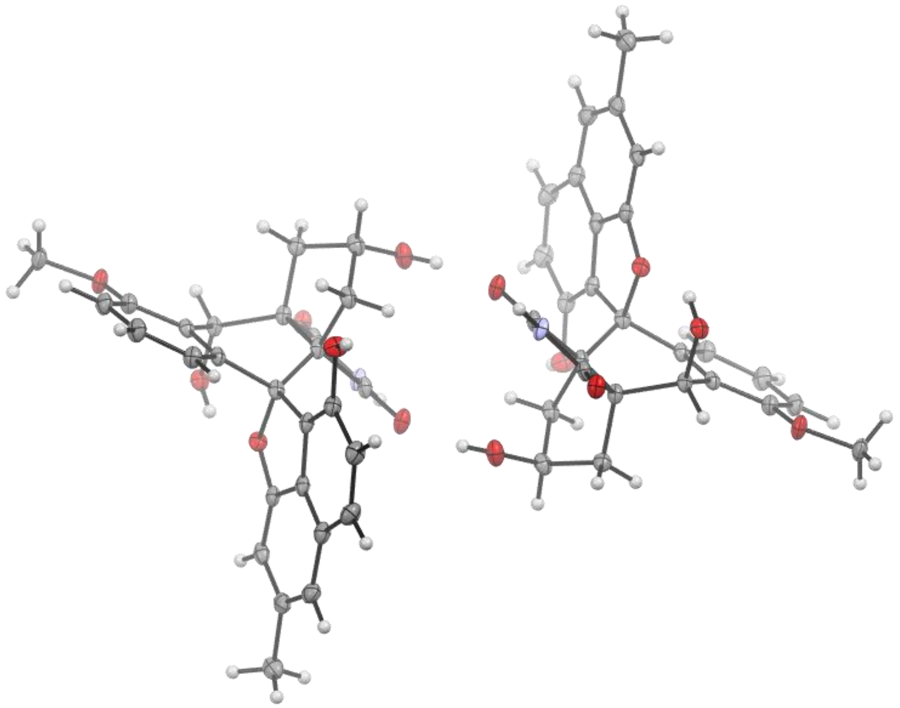

Figure S7: ORTEP 50% probability representation showing both enantiomers.

A single crystal of compound **12** was mounted on top of a cryoloop and transferred into the cold nitrogen stream (100 K) of a Bruker-AXS D8 Venture diffractometer. Data collection and reduction was done using the Bruker software suite APEX3.[i] The final unit cell was obtained from the xyz centroids of 5614 reflections after integration. A multiscan absorption correction was applied, based on the intensities of symmetry-related reflections measured at different angular settings (*SADABS*). The structures were solved by direct methods using *SHELXT*[ii] and refinement of the structure was performed using *SHLELXL*.[iii] From the solution it was clear that a disordered solvent molecule (toluene) was present, which could not be satisfactorily modeled during refinement. Its contribution was removed using the PLATON/SQUEEZE routine.[iv] The hydrogen atoms were generated by geometrical considerations, constrained to idealized geometries and allowed to ride on their carrier atoms with an isotropic displacement parameter related to the equivalent displacement parameter of their carrier atoms.

[I] Bruker, (2016). *APEX3* (v2016.1-0), *SAINT* (Version 8.18C) and *SADABS* (Version 2012/1). Bruker AXS Inc., Madison, Wisconsin, USA.
[ii] Sheldrick, G. M. (2015) *Acta Cryst.* **A71**, 3-8
[iii] Sheldrick, G. M. (2008). *Acta Cryst.* A**64**, 112-122
[iv] Spek, A. L. (2015). *Acta Cryst.* **C71**, 9-18

Table S5: Crystal data of compound **12**.

chem formula	C23 H27 N O7
Mr	429.45
cryst syst	triclinic
color, habit	colourless, block
size (mm)	0.251 x 0.056 x 0.026
space group	P -1
a (Å)	9.0194(2)
b (Å)	9.5059(2)
c (Å)	15.0707(3)
α, deg	97.4480(10)
β, deg	104.7470(10)
γ, deg	91.5730(10)
V (Å3)	1236.55(5)
Z	2
ρ_{calc}, $g.cm^{-3}$	1.153
μ(Cu K^{α}), cm^{-1}	0.709
F(000)	456
temp (K)	100(2)
θ range (deg)	3.063 - 72.189
data collected (h,k,l)	-10:11, -11:11, -18:18
no. of rflns collected	13929
no. of indpndt reflns	4793
observed reflns	3936 (Fo ≥ 2 σ(Fo))
R(F) (%)	4
wR(F2) (%)	10.5
GooF	1.027
Weighting a,b	0.0454, 0.5541
params refined	321
restraints	0
min, max resid dens	-0.230, 0.527

Section 4: Experiments with elmonin

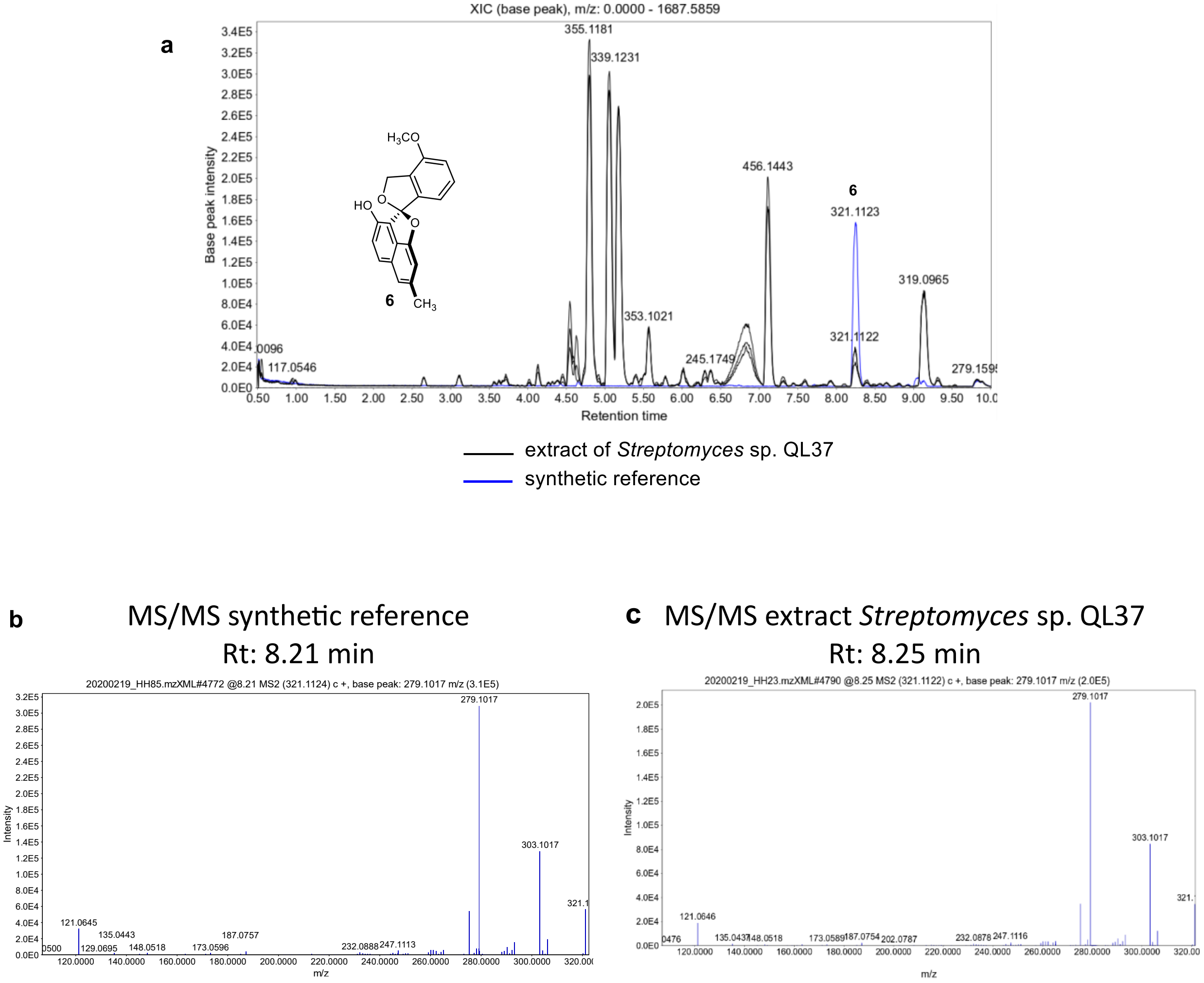

Figure S8: Analytical detection of elmonin in *Streptomyces* sp. QL37. **a.** A reference sample of synthetic elmonin was injected on LC-MS and also an extract of *Streptomyces* sp. QL37. The chromatogram of the extract shows a match with the synthetic reference. **b**. MS/MS spectrum corresponding to the peak at 8.21 min in the chromatogram of the extract. **c**. MS/MS spectrum of the chromatogram of the synthetic sample of elmonin **6**. The bacterial extract and synthetic reference were prepared as previously reported[7, 14]. LC-MS acquisition was performed using Shimadzu Nexera X2 UHPLC system, with attached PDA, coupled to a Shimadzu 9030 QTOF mass spectrometer, equipped with a standard ESI source unit, in which a calibrant delivery system (CDS) is installed. The dry extract and synthetic compound were dissolved in MeOH to a final concentration of 1 mg/mL, and 2 µL were injected into a Waters Acquity HSS C 18 column (1.8 µm, 100 Å, 2.1 × 100 mm). The column was maintained at 30 °C, and run at a flow rate of 0.5 mL/min, using 0.1% formic acid in H_2O as solvent A, and 0.1% formic acid in acetonitrile as solvent B. A gradient was employed for chromatographic separation starting at 5% B for 1 min, then 5 – 85% B for 9 min, 85 – 100% B for 1 min, and finally held at 100% B for 4 min. The column was re-equilibrated to 5% B for 3 min before the next run was started. The LC flow was switched to the waste the first 0.5 min, then to the MS for 14.5 min, then back to the waste to the end of the run. The PDA acquisition was performed in the range 200 – 600 nm, at 4.2 Hz, with 1.2 nm slit width. The flow cell was maintained at 40 °C.

A

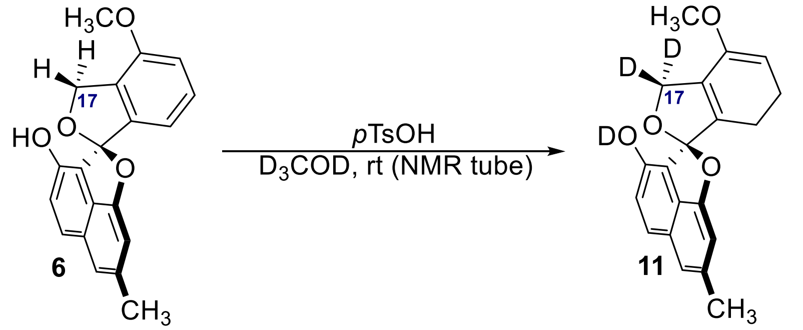

B

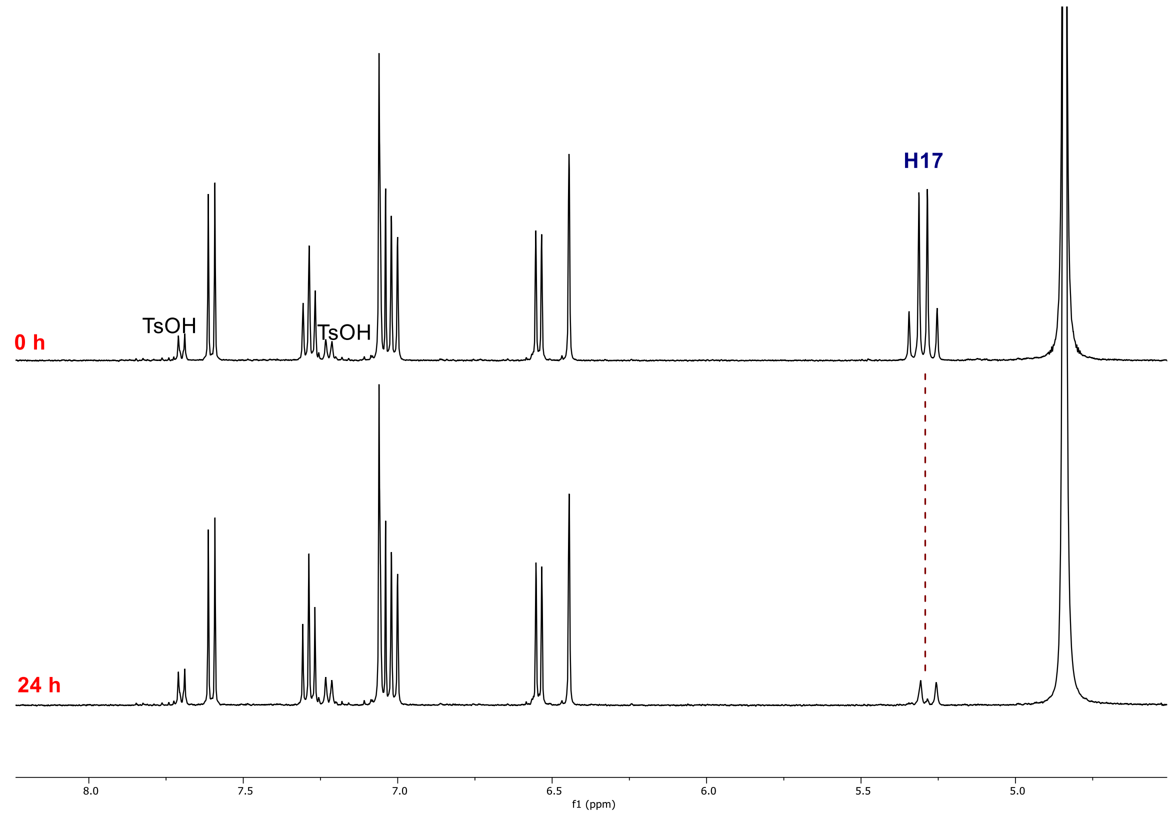

Figure S9: A. Reaction of elmonin in D$_3$COD, a crystal of catalytic *p*TsOH was added and the mixture was transferred to an NMR tube, deuterium exchange with H17 was evident after 24 h of standing at rt. **B.** Stacked spectra showing clear disappearance of the signal corresponding to H17. Spectra were recorded on an Agilent 400 NMR spectrometer at 400 MHz.

Section 5: NMR spectra of synthetic compounds 1, 12 and 14

Figure S10: ^{1}H-NMR (600 MHz, D_3COD) synthetic lugdunomycin **1**, after preparatory HPLC.

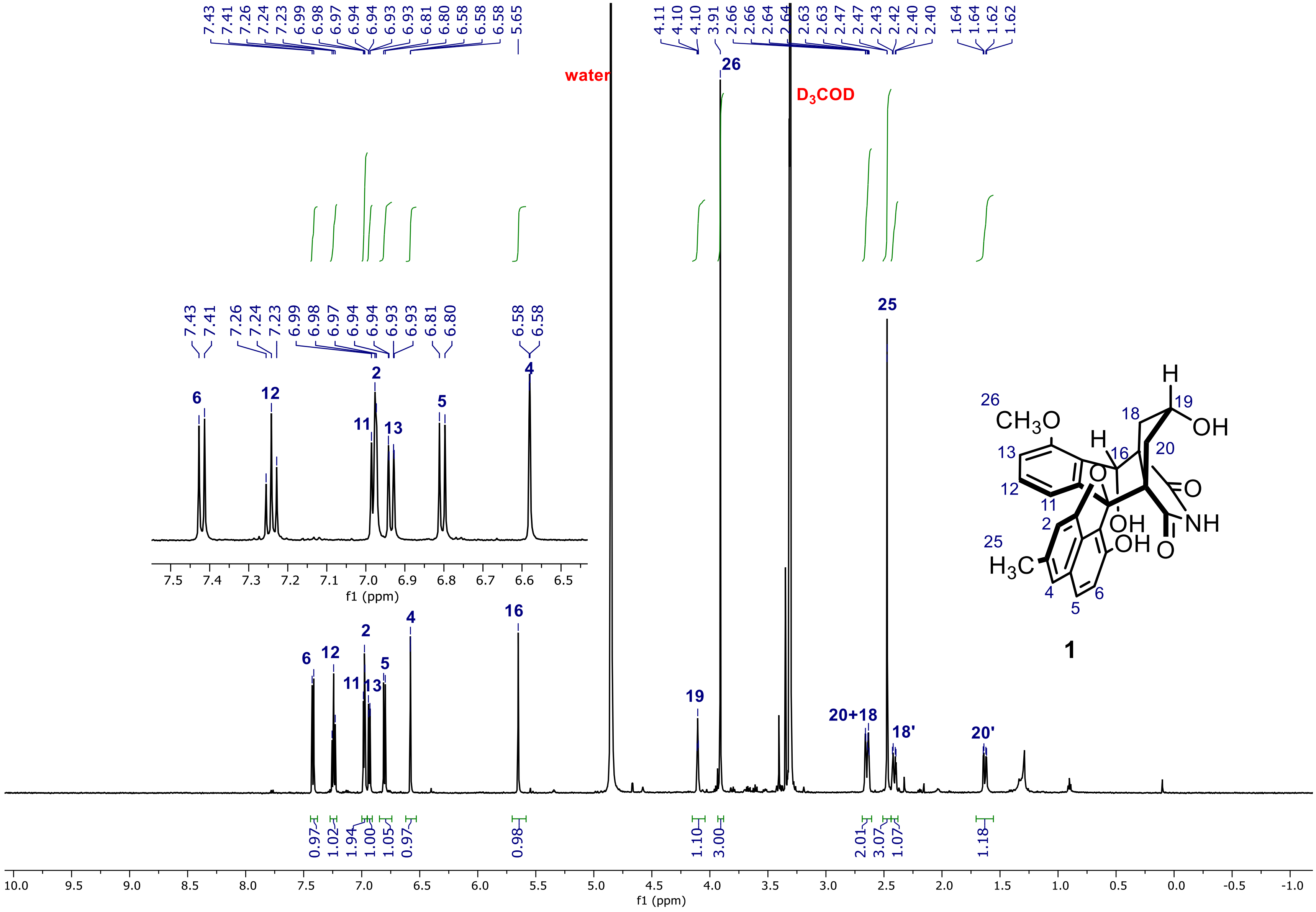

Figure S11: {^{1}H}^{13}C APT (151 MHz, D_3COD) synthetic lugdunomycin **1**, after preparatory HPLC.

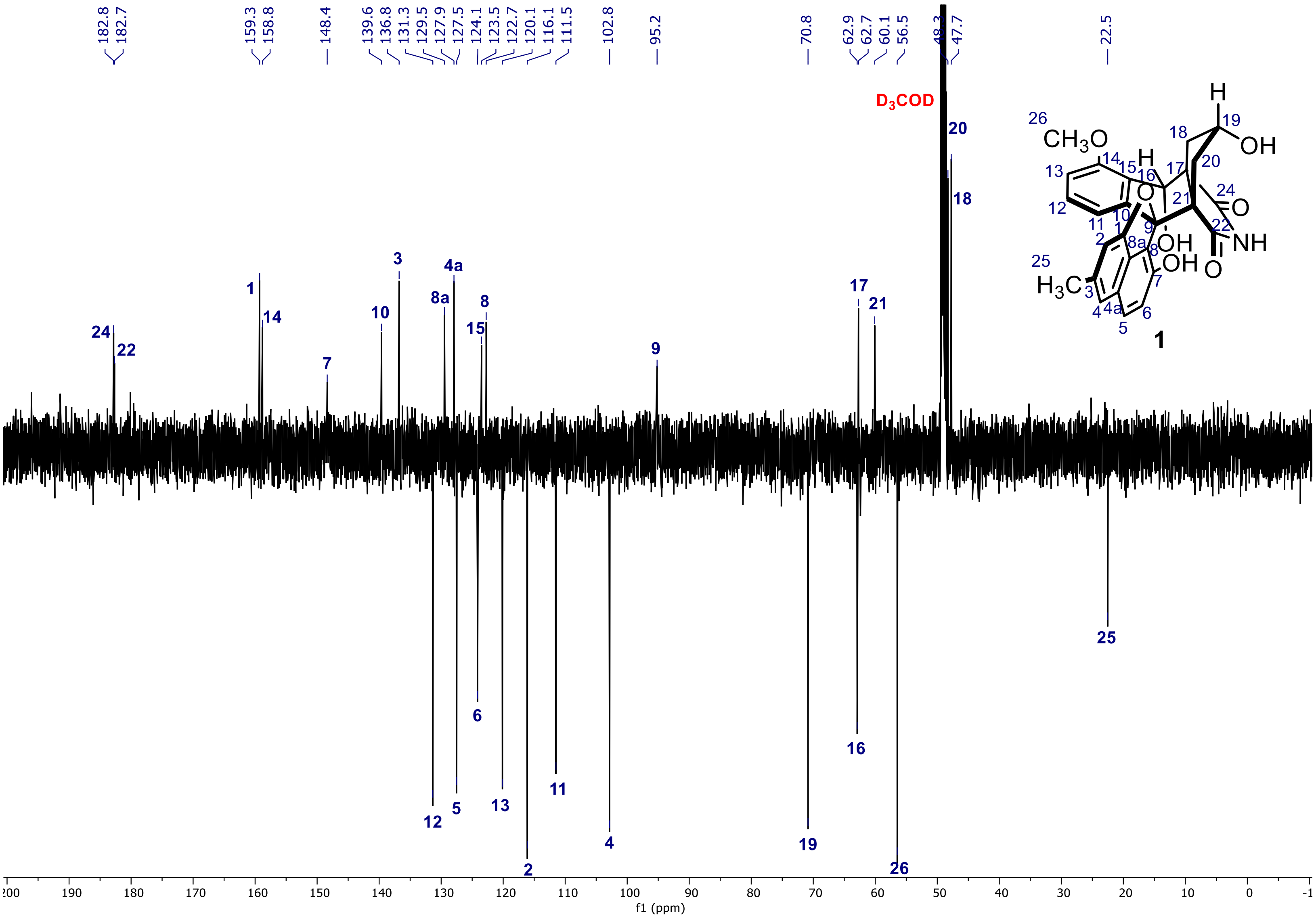

Figure S12: ^{1}H-NMR (600 MHz, D_3COD) *epi*-lugdunomycin **12**.

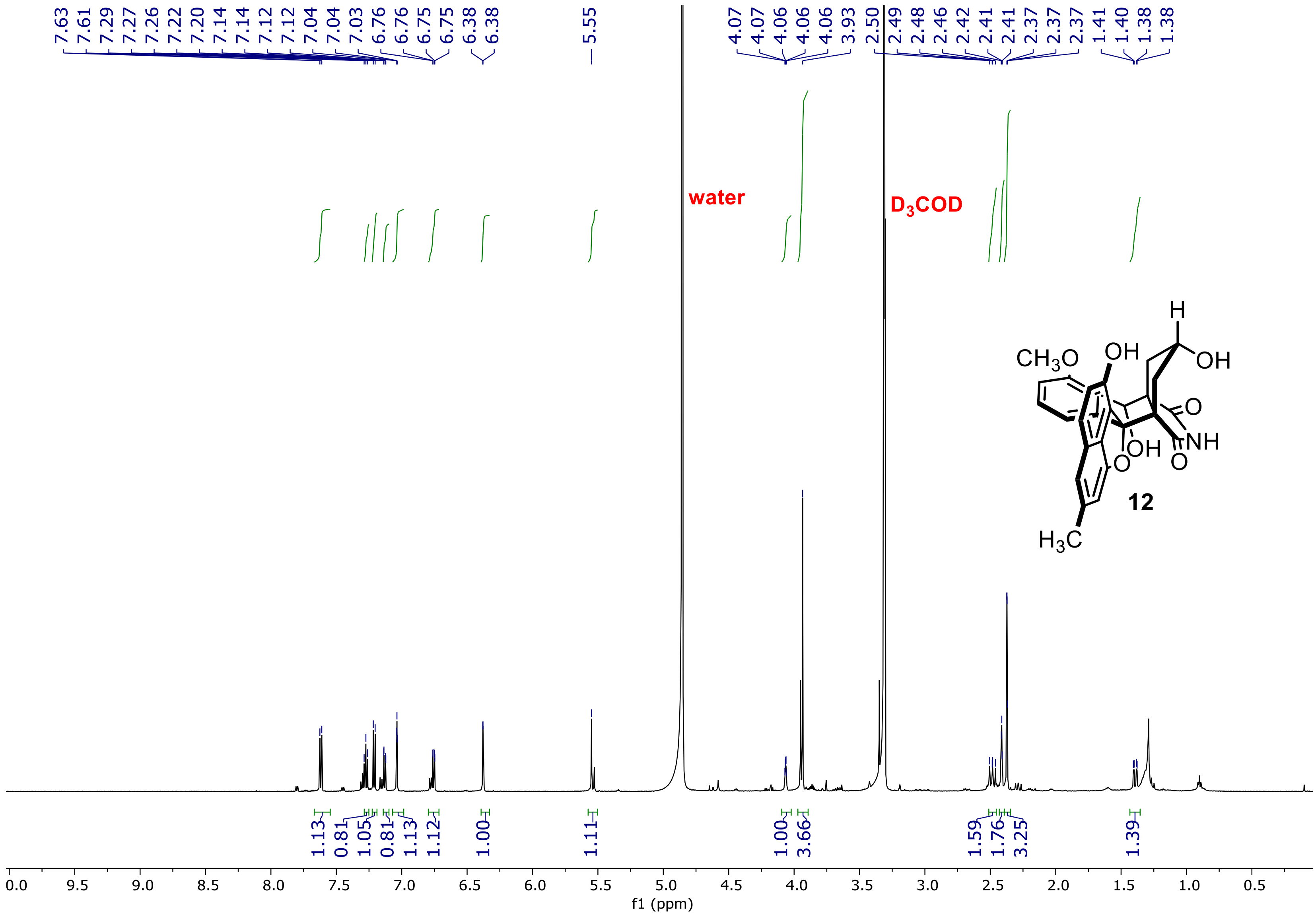

12

Figure S13: {^{1}H}^{13}C APT (151 MHz, D3COD) *epi*-lugdunomycin **12**.

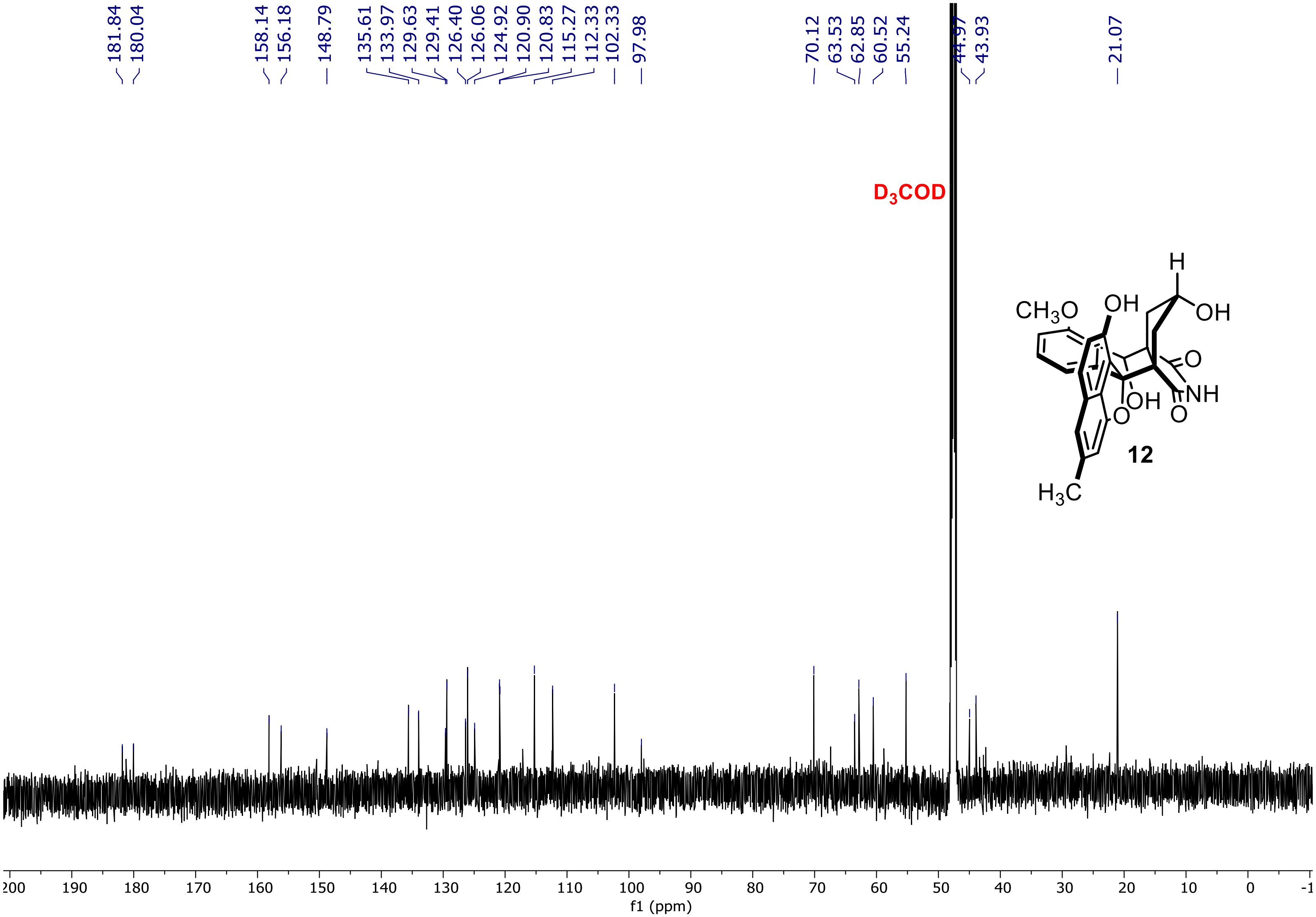

Figure S14: $^{1}H/^{1}H$-NOESY (600 MHz, D_3COD) *epi*-lugdunomycin **12**.

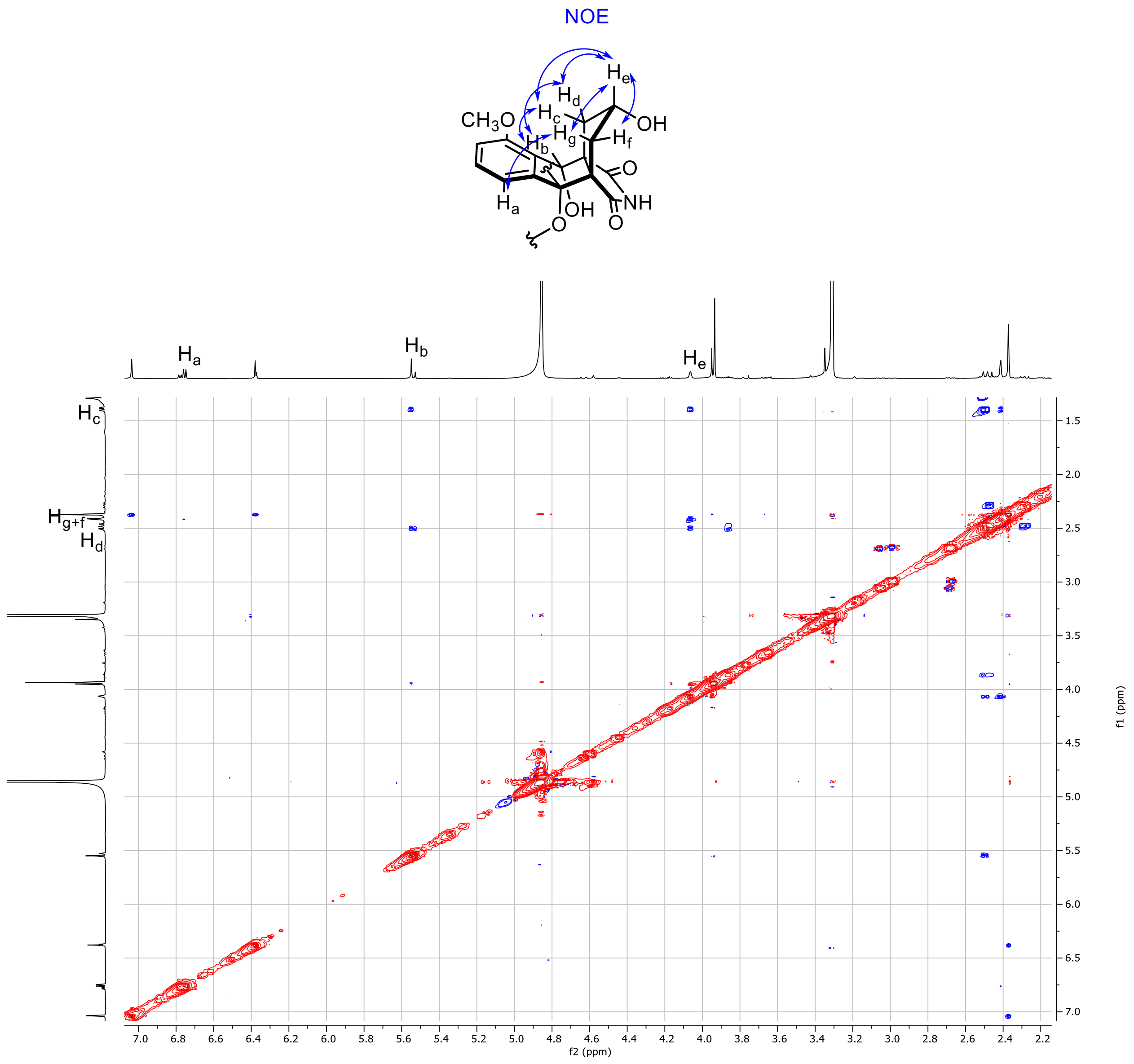

Figure S15: ^{1}H-NMR (600 MHz, D_3COD) cyclisation product **14**.

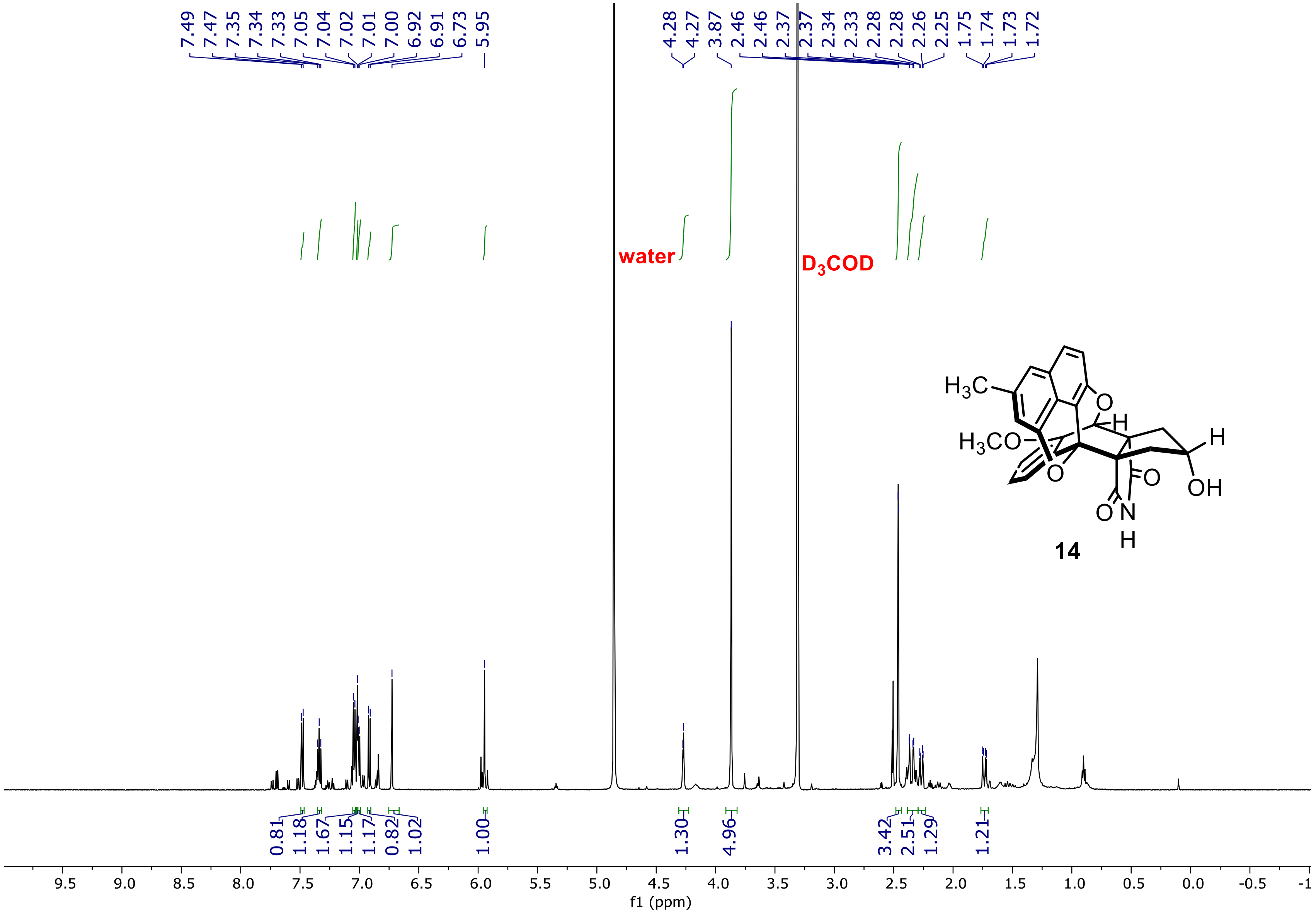

Figure S16: {^{1}H}^{13}C-NMR (151 MHz, D_3COD) cyclisation product **14**.

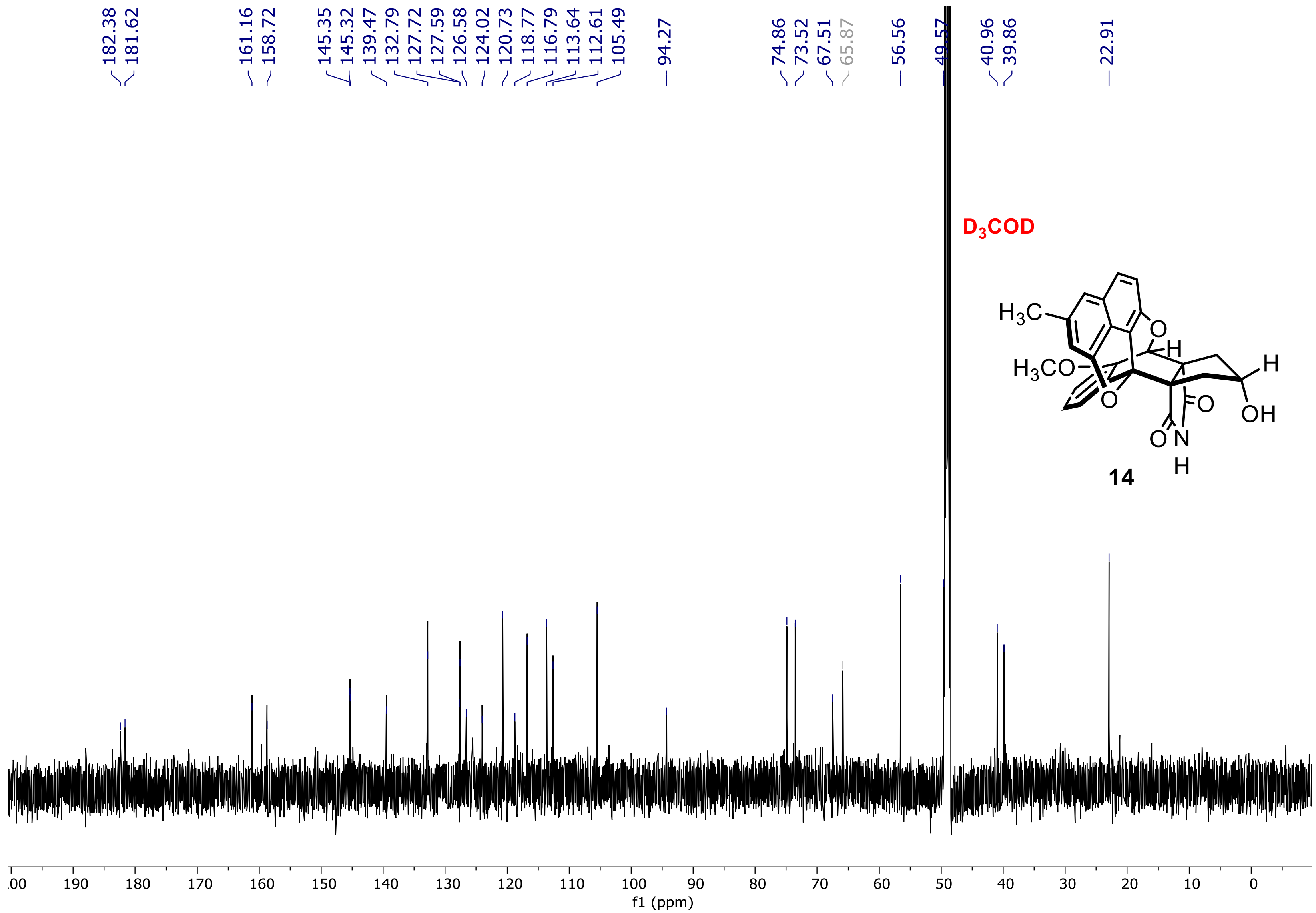

Figure S17: $^1H/^1H$-NOESY (500 MHz, D_3COD) spectrum of cyclisation product **14**.

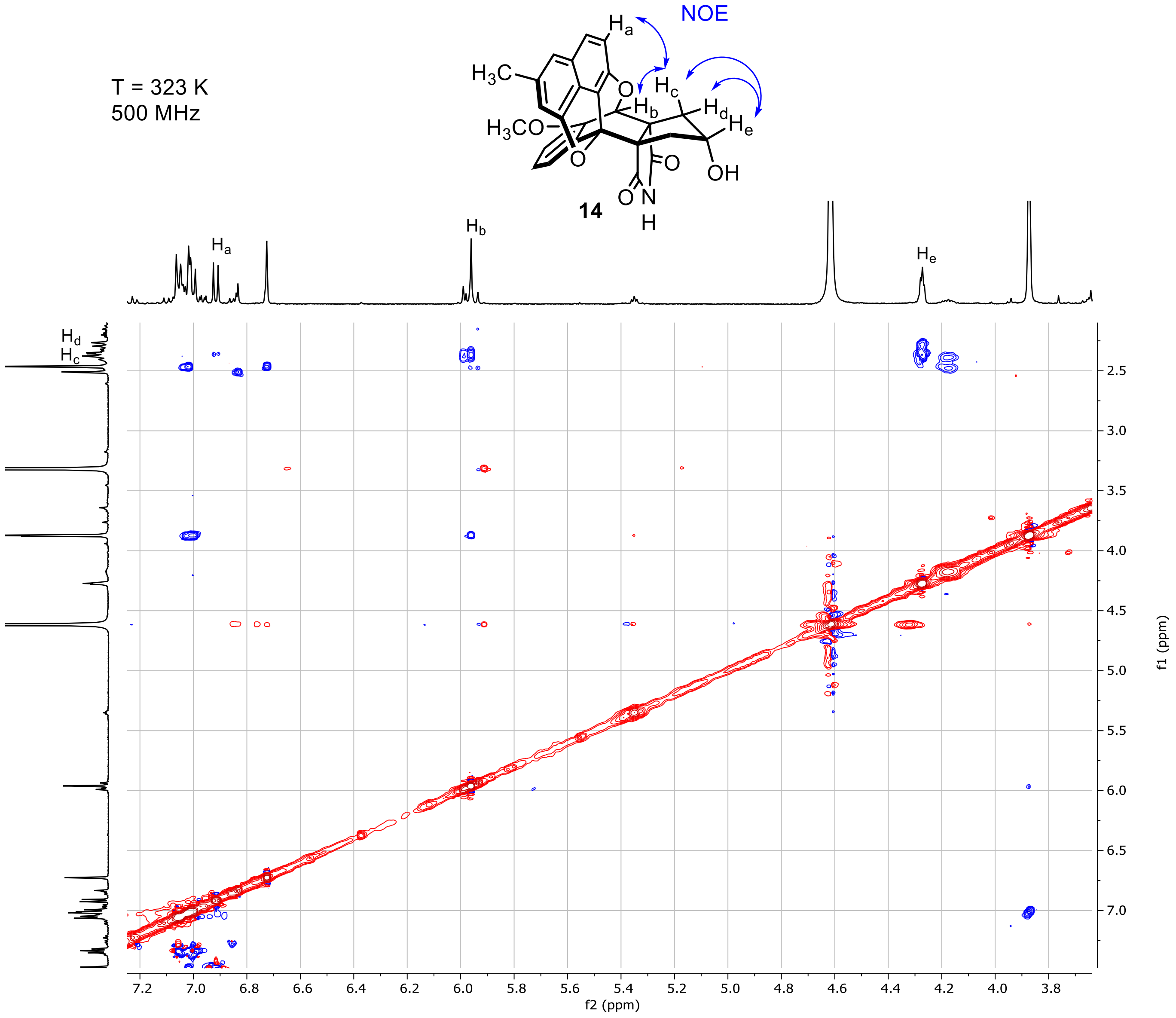

Section 6: HRMS spectra of synthetic compounds 1, 12, 14

ESI (+) mode, unless mentioned otherwise.

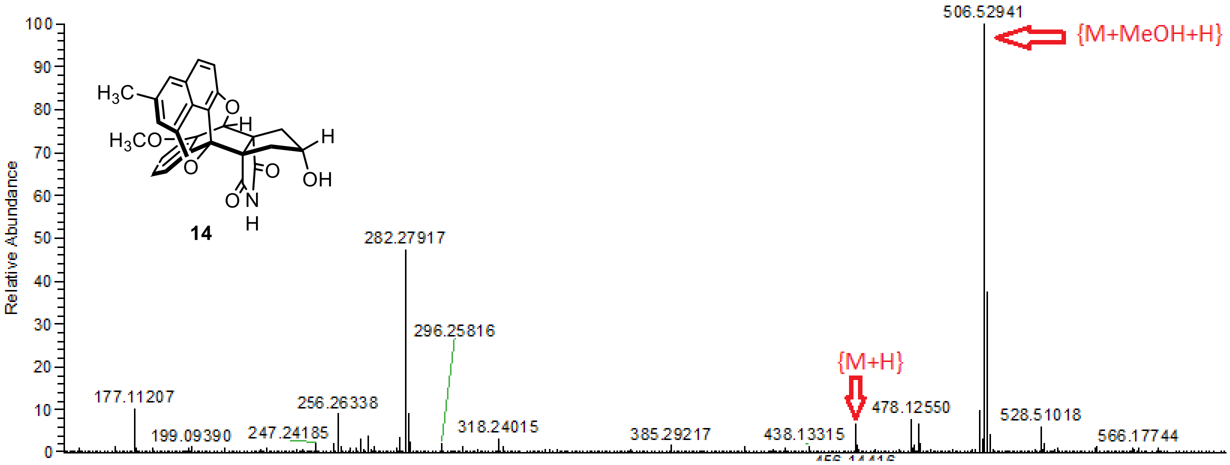

Figure S18: ESI HRMS spectrum of compound **14**, (+)-mode.

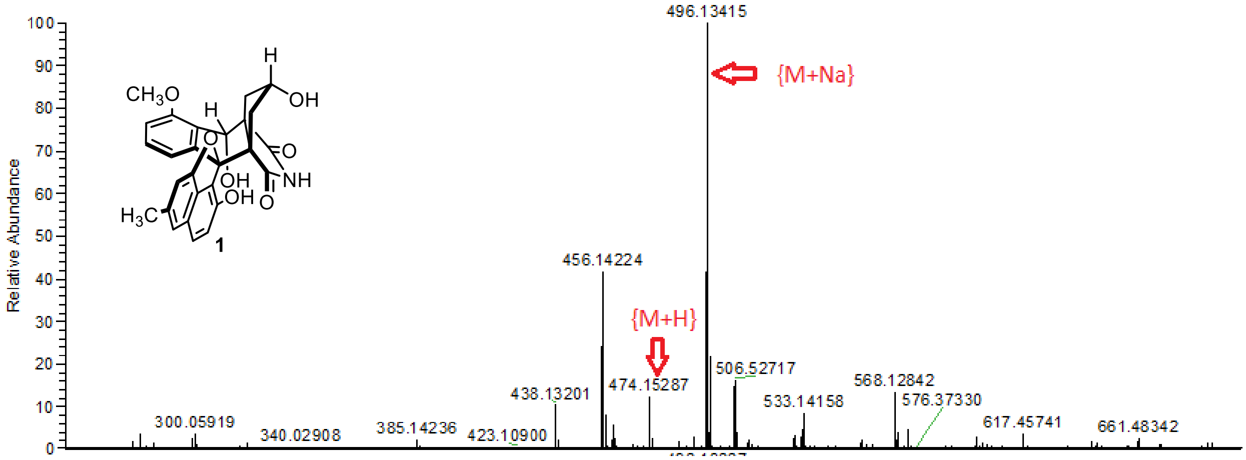

Figure S19: ESI HRMS spectrum of lugdunomycin **1**, (+)-mode.

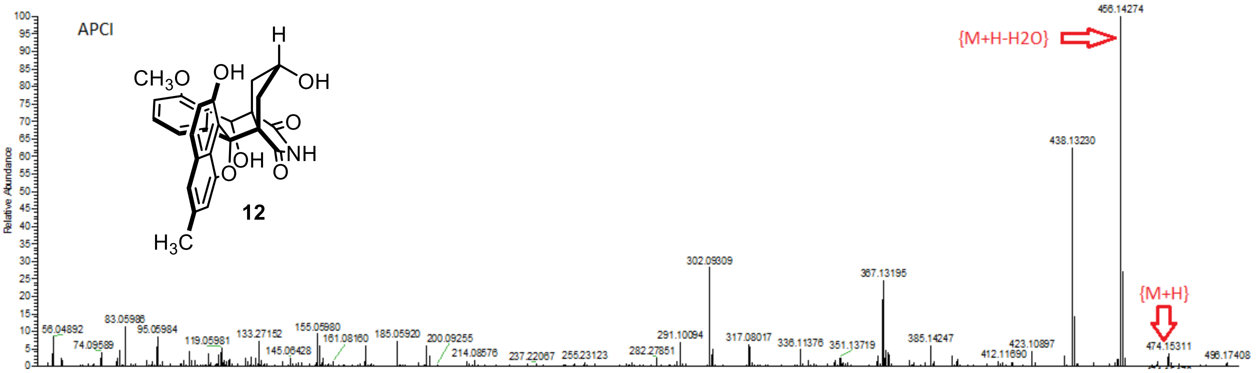

Figure S20: APCI HRMS spectrum of *epi*-lugdunomycin **12**, (+)-mode.

Section 7: Density Functional Theory (DFT) calculations

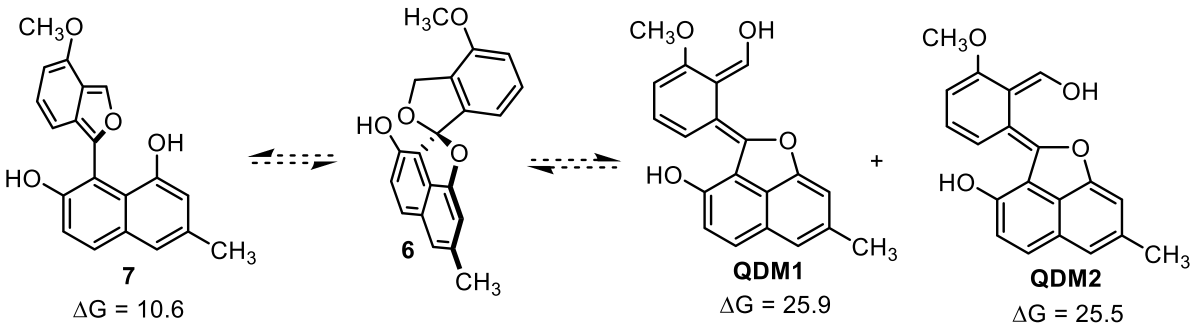

Figure S21: Gas phase thermodynamic values for the formation of **7** and quinodimethane **QDM1**/**2** species, values in kcal/mol, calculated at the TZ2P/M06-2x//TZ2P/B3LYP level of theory, in the gas phase at standard temperature and pressure, using the AMS modelling suite[15, 16]. ΔG is relative to the calculated Gibbs energy of elmonin. The results suggest **7** is a likely diene candidate formed from elmonin.

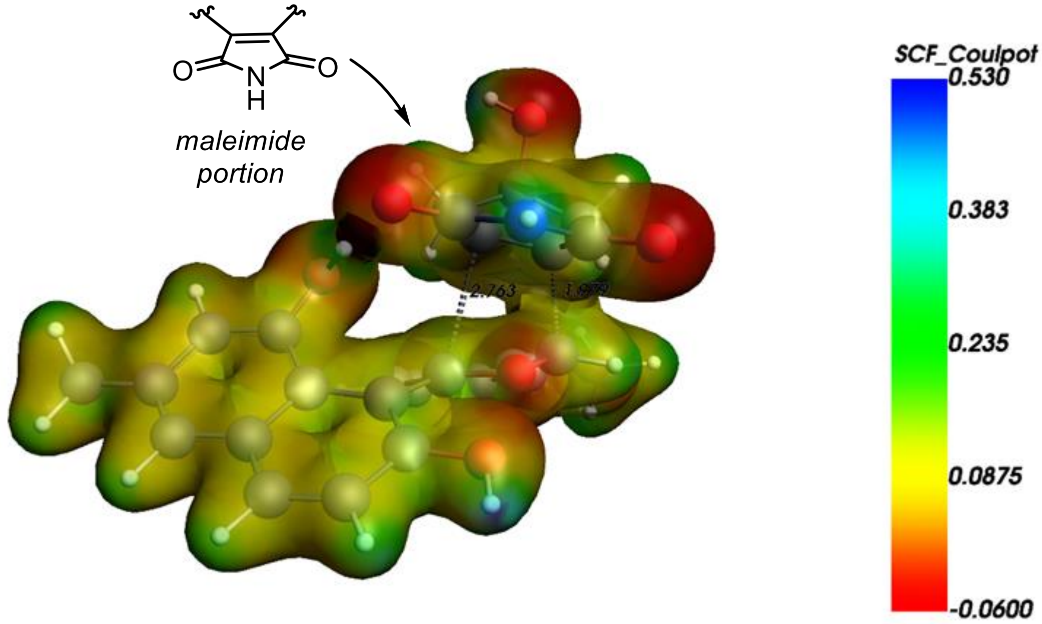

Figure S22: Optimised structure of **TS1** with the electrostatic potential plotted on a charge density isosurface, computed at TZ2P/M06-2x//B3LYP-D3(BJ) level of theory in the gas phase. A relatively low electrostatic potential (red) implies a higher electron density. A relatively high electrostatic potential (blue) implies a low electron density. No significant charge build-up is observed in the transition state, suggesting a concerted [4+2] reaction.

Table S6: Kinetic and thermodynamic values of the stationary points in the eight diastereomeric pathways of the reaction between **7** and **2** Values in kcal/mol, calculated at the TZ2P/M06-2x//TZ2P/B3LYP-D3(BJ) level of theory, in the gas phase at standard temperature and pressure, using the AMS modelling suite.[5]

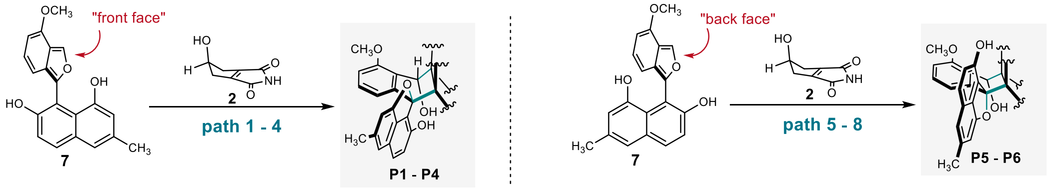

path 1 (*exo*)	path 2 (*exo*)	path 3 (*endo*)	path 4 (*endo*)
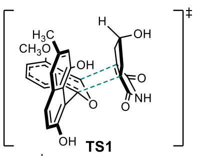 TS1 $\Delta G^{\ddagger}$ = 20.9 kcal/mol	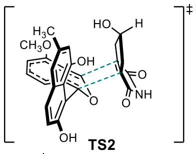 TS2 $\Delta G^{\ddagger}$ = 21.1 kcal/mol	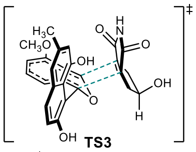 TS3 $\Delta G^{\ddagger}$ = 26.1 kcal/mol	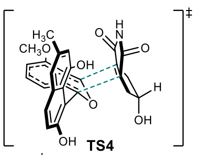 TS4 $\Delta G^{\ddagger}$ = 23.3 kcal/mol
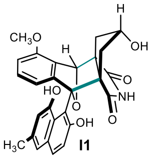 I1 ΔG = -9.3 kcal/mol	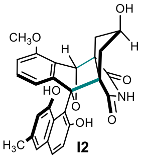 I2 ΔG = -6.9 kcal/mol	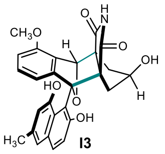 I3 ΔG = -2.7 kcal/mol	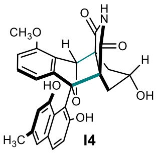 I4 ΔG = -0.3 kcal/mol
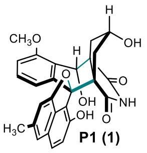 P1 (1) ΔG = -27.5 kcal/mol	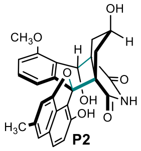 P2 ΔG = -26.3 kcal/mol	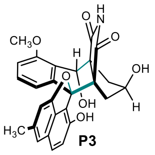 P3 ΔG = -21.0 kcal/mol	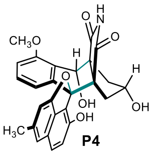 P4 ΔG = -23.6 kcal/mol

path 5 (*exo*)	path 6 (*exo*)	path 7 (*endo*)	path 8 (*endo*)
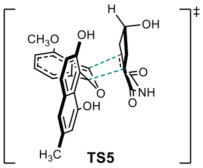 TS5 $\Delta G^{\ddagger}$ = 21.9 kcal/mol	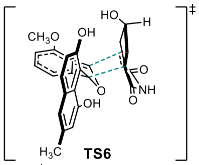 TS6 $\Delta G^{\ddagger}$ = 22.8 kcal/mol	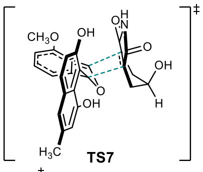 TS7 $\Delta G^{\ddagger}$ = 23.3 kcal/mol	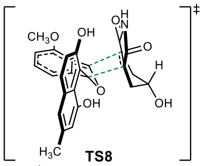 TS8 $\Delta G^{\ddagger}$ = 21.5 kcal/mol
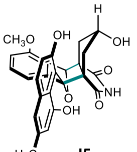 I5 ΔG = -12.3 kcal/mol	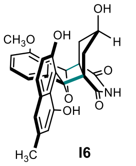 I6 ΔG = -9.8 kcal/mol	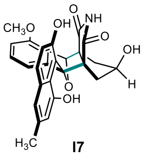 I7 ΔG = -13.5 kcal/mol	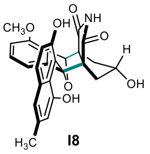 I8 ΔG = -11.3 kcal/mol
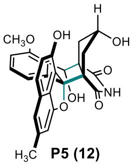 P5 (12) ΔG = -27.5 kcal/mol	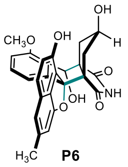 P6 ΔG = -25.9 kcal/mol	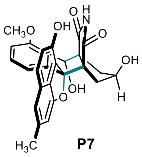 P7 ΔG = -26.7 kcal/mol	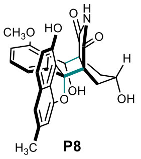 P8 ΔG = -24.3 kcal/mol

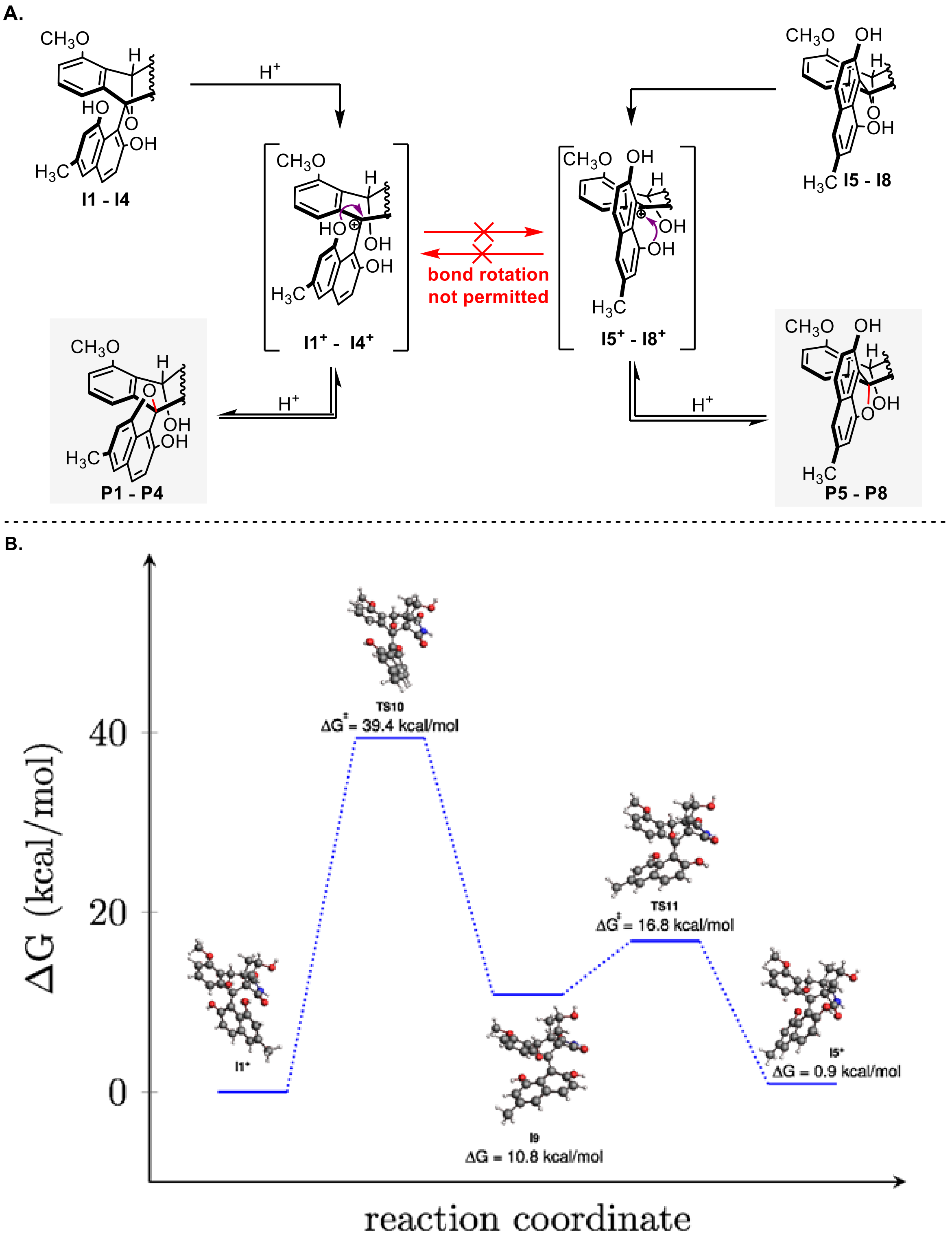

Figure S23: A. Acid catalysed ring-opening in Diels-Alder cycloadducts **I1** – **I4**/ **I5** – **I8** or ring opening of **P1** – **P4**/ **P5** – **P6** leads to formation of carbocations **I1**$^+$ – **I4**$^+$/ **I5**$^+$ – **I8**$^+$. Interconversion by means of bond rotation between **I1**$^+$ –

I4⁺ and **I5⁺** – **I6⁺** is considered unlikely based on computations and therefore not a possible isomerisation pathway. **B.** The calculation path of interconversion between **I1⁺** and **I5⁺** by means of bond rotation suggests a maximum rotational barrier of 39.4 kcal/mol. Gas phase energies computed at TZ2P/M06-2x//TZ2P/B3LYP-D3(BJ) using the AMS modelling suite[16].

Computational method

Input geometries were generated using SMILES extracted from the CambridgeSoft ChemBioDraw package and pre-optimised in AMS using the UFF forcefield and then optimised as a normal stationary point or as a transition state at DFT (B3LYP or B3LYP-D3(BJ)) using the TZ2P basis set in AMS. This was followed by a frequency analysis using the same functional/basis set combination as has been used for the optimizations in AMS[16, 38]. The electronic energies were re-calculated with a single point calculation using the M06-2x functional and TZ2P basis set in AMS, then the Gibbs free energies were re-calculated.

Optimized geometries

Atomic cartesian coordinates given in angstrom.

Table S7: elmonin **6**: TZ2P/B3LYP

1 C	-4.06229	0.378224	1.480032
2 C	-4.18495	1.784249	1.326424
3 C	-3.13783	2.457844	0.743753
4 C	-2.02632	1.723944	0.344049
5 C	-1.85287	0.343964	0.473629
6 C	-2.94558	-0.32597	1.074786
7 C	-1.06822	2.575471	-0.22436
8 C	0.109501	2.063855	-0.68797
9 C	0.32367	0.652002	-0.57361
10 C	-0.62309	-0.1808	-0.01284
11 O	-5.29803	2.458702	1.737881
12 H	-2.91432	-1.39846	1.222643
13 C	1.626854	0.095028	-1.08806
14 H	1.681309	-0.98413	-0.94903
15 H	-0.42789	-1.24392	0.056366
16 H	-5.92887	1.841439	2.123323
17 H	0.868449	2.695363	-1.13079
18 H	2.477915	0.54591	-0.57149
19 H	1.75124	0.305598	-2.15332
20 H	-4.88529	-0.15944	1.939519
21 C	-2.86034	3.913401	0.430539
22 O	-2.77021	4.691966	1.591246
23 C	-3.29855	6.014806	1.379634
24 C	-4.02341	5.914302	0.074928
25 C	-3.79695	4.671419	-0.47795
26 C	-4.82565	6.845891	-0.58102
27 C	-5.4001	6.48336	-1.79902
28 C	-5.16262	5.21671	-2.33956

29 C	-4.3559	4.292982	-1.6911
30 O	-4.98685	8.049927	0.037835
31 C	-5.7993	9.030089	-0.59218
32 O	-1.51738	3.861338	-0.22979
33 H	-2.48234	6.742817	1.352315
34 H	-3.95091	6.255144	2.220488
35 H	-6.03495	7.173111	-2.33519
36 H	-5.62185	4.960283	-3.28557
37 H	-4.17109	3.315884	-2.11607
38 H	-5.78657	9.896831	0.063224
39 H	-6.82807	8.678925	-0.70851
40 H	-5.39897	9.309366	-1.57031

Table S8: isobenzofuran **7:** TZ2P/B3LYP

1 C	-3.9727	0.058568	1.010968
2 C	-4.27379	1.400898	0.70928
3 C	-3.27357	2.330048	0.462112
4 C	-1.90825	1.886871	0.474077
5 C	-1.60611	0.533277	0.821788
6 C	-2.67401	-0.35464	1.085758
7 C	-0.79061	2.705331	0.119226
8 C	0.494672	2.224447	0.193803
9 C	0.784501	0.901473	0.590767
10 C	-0.26719	0.074887	0.884591
11 O	-5.57427	1.820624	0.689912
12 H	-2.44608	-1.38148	1.342234
13 H	-6.23856	6.976947	-2.92904
14 C	-5.72248	9.001978	-1.46141
15 H	-0.08936	-0.9555	1.167622
16 H	-6.15092	1.086916	0.926959
17 H	1.312056	2.884833	-0.08014
18 H	-6.1784	4.613494	-3.57017
19 C	2.214657	0.437961	0.661376
20 H	-4.78239	-0.63511	1.209894
21 C	-3.66603	3.728242	0.257108
22 O	-3.26932	4.636235	1.195911
23 C	-3.71514	5.863539	0.84272
24 C	-4.42086	5.771057	-0.32573
25 C	-4.39795	4.378212	-0.70901
26 C	-5.10067	6.724448	-1.14339
27 C	-5.71441	6.28756	-2.28416
28 C	-5.67707	4.902414	-2.65503
29 C	-5.04414	3.958847	-1.9059

30 O	-5.06421	8.00772	-0.69173
31 H	-5.03233	2.919792	-2.20408
32 O	-1.03387	3.970639	-0.32977
33 H	-0.19807	4.391337	-0.55846
34 H	-3.47563	6.674437	1.50476
35 H	-5.29192	9.075935	-2.46367
36 H	-5.57487	9.940185	-0.93317
37 H	-6.79266	8.794093	-1.54366
38 H	2.783716	1.025673	1.38595
39 H	2.71394	0.547082	-0.30452
40 H	2.276506	-0.60909	0.95571

Table S9: isobenzofuran 7: TZ2P/B3LYP-D3(BJ)

1	C	1.704381	2.660518	2.754571
2	C	-1.64541	1.351383	3.334812
3	C	-1.17974	0.265282	2.661701
4	C	-0.741	0.454333	1.32125
5	C	-0.81167	1.765719	0.713719
6	C	-1.29764	2.877128	1.46913
7	C	-0.38084	1.630672	-0.57387
8	O	-0.03481	0.333136	-0.79674
9	C	-0.23688	-0.40155	0.358621
10	C	-0.07413	-1.84914	0.297448
11	C	-0.70159	-2.538	-0.7378
12	C	-0.80689	-3.94039	-0.72089
13	C	-0.2914	-4.65097	0.321218
14	C	0.41954	-4.01975	1.369304
15	C	0.576782	-2.59914	1.340152
16	C	1.439481	-2.04268	2.335872
17	C	1.976894	-2.83309	3.325779
18	C	1.754057	-4.21997	3.38555
19	C	0.994934	-4.79605	2.399291
20	C	2.374847	-5.02994	4.489808
21	O	-1.31222	-1.92263	-1.78586
22	O	1.838545	-0.74083	2.346893
23	O	-1.30909	4.05987	0.807961
24	C	-1.78427	5.201887	1.508564
25	H	-2.08141	3.471821	3.357872
26	H	-1.98762	1.23905	4.354947
27	H	-1.13319	-0.71004	3.122709
28	H	-0.26041	2.310714	-1.39558
29	H	-1.33008	-4.42046	-1.53608
30	H	-0.3981	-5.72774	0.347403
31	H	2.617184	-2.35097	4.053395

32	H	0.836181	-5.86702	2.385349
33	H	2.080418	-6.07669	4.425527
34	H	3.465358	-4.98124	4.445466
35	H	2.075476	-4.65178	5.469553
36	H	-1.06746	-0.98902	-1.80634
37	H	1.335235	-0.22671	1.703056
38	H	-1.71035	6.033471	0.814136
39	H	-2.82493	5.068381	1.81346
40	H	-1.17057	5.402802	2.389803

Table S10: *iso*-maleimycin **2:** TZ2P/B3LYP-D3(BJ)

1	H	2.313774	1.006914	1.466377
2	H	3.957729	1.072954	0.844306
3	H	4.757459	3.374446	1.026851
4	H	2.515136	3.384278	1.590305
5	C	2.870399	2.985139	0.637951
6	C	1.912101	3.361006	-0.52906
7	C	1.857017	2.092721	-1.31075
8	C	2.407104	1.060369	-0.67456
9	C	2.938709	1.421668	0.670758
10	C	1.331394	1.642325	-2.63327
11	N	1.639499	0.274841	-2.67601
12	C	2.299322	-0.16452	-1.51961
13	H	1.427567	-0.32187	-3.45935
14	O	0.7676	2.267843	-3.49509
15	O	2.675254	-1.28912	-1.30324
16	O	4.135789	3.556614	0.314319
17	H	0.922172	3.647425	-0.1663
18	H	2.314048	4.205342	-1.08813

Table S11: QDM1: TZ2P/B3LYP

1 C	1.175657	0.477429	-0.37434
2 C	2.613801	0.038123	-0.26942
3 C	3.597381	1.024057	0.041768
4 C	4.895304	0.605051	0.125569
5 O	6.02417	1.336535	0.414741
6 C	5.259777	-0.71961	-0.07908
7 C	4.317415	-1.71165	-0.3646
8 C	4.853941	-3.01293	-0.49702
9 C	6.20802	-3.20922	-0.31333
10 C	7.123088	-2.17714	-0.00458

11 O	8.437127	-2.48369	0.222183
12 C	6.656171	-0.86546	0.08467
13 C	7.161963	0.474101	0.356282
14 C	8.401121	1.067679	0.446078
15 C	9.571317	0.336847	0.030565
16 C	10.75057	0.960805	-0.19476
17 C	10.9119	2.371702	-0.03007
18 C	9.885311	3.097915	0.477679
19 O	9.929573	4.441284	0.691132
20 C	11.13921	5.130452	0.410496
21 C	8.624915	2.464375	0.884461
22 C	7.875579	3.174749	1.780649
23 O	6.747984	2.827347	2.398238
24 C	2.965417	-1.28441	-0.4632
25 H	1.049069	1.225412	-1.16051
26 H	0.835958	0.933371	0.55902
27 H	0.518453	-0.3618	-0.5973
28 H	3.31476	2.055508	0.203081
29 H	4.21455	-3.8562	-0.72329
30 H	6.603588	-4.21664	-0.39606
31 H	8.561801	-3.43572	0.143307
32 H	9.478124	-0.71491	-0.17384
33 H	11.59248	0.384613	-0.55723
34 H	11.84642	2.833239	-0.30733
35 H	11.96472	4.740809	1.011176
36 H	10.95773	6.170213	0.669078
37 H	11.39655	5.058326	-0.64901
38 H	8.23746	4.137008	2.110474
39 H	6.283663	2.146501	1.87379
40 H	2.19517	-2.01152	-0.68898

Table S12: QDM2: TZ2P/B3LYP

1 O	1.582974	1.035817	0.567251
2 C	2.728215	0.424784	0.14479
3 C	2.909518	-0.86169	-0.28576
4 C	4.223935	-1.23697	-0.69063
5 C	4.446994	-2.6517	-1.16216
6 C	5.269105	-0.33244	-0.65704
7 C	5.071716	1.004062	-0.21542
8 C	6.000889	2.066456	-0.14493
9 C	5.575807	3.310181	0.277249
10 C	4.249155	3.604974	0.665053
11 O	3.92387	4.890552	1.009337
12 C	3.299085	2.581857	0.661602

13	C	1.881958	2.36135	0.954799
14	H	-0.4781	1.347543	-0.03763
15	C	0.879223	3.090894	1.556026
16	C	-0.53054	2.629652	1.662741
17	C	-1.35116	3.268047	2.692857
18	O	-2.564	2.650852	2.918541
19	C	-3.45003	3.213963	3.880054
20	C	-0.95605	4.387781	3.338944
21	C	0.323764	4.955087	3.03099
22	C	1.191488	4.344719	2.193921
23	C	-1.0651	1.740778	0.773818
24	O	-2.32002	1.270959	0.745493
25	C	3.776273	1.337068	0.190952
26	H	2.165284	4.772956	2.03907
27	H	2.096217	-1.57394	-0.31952
28	H	3.814043	-2.88429	-2.0219
29	H	4.199685	-3.37097	-0.37763
30	H	5.483915	-2.8146	-1.45346
31	H	6.255022	-0.64542	-0.97816
32	H	7.033785	1.921389	-0.43393
33	H	6.294484	4.123287	0.306388
34	H	4.704435	5.449024	0.926272
35	H	-2.7944	1.627719	1.518112
36	H	-3.73982	4.228242	3.598105
37	H	-2.98834	3.225733	4.869034
38	H	-4.32651	2.571894	3.893925
39	H	-1.58158	4.864415	4.077182
40	H	0.610967	5.876827	3.520025

Table S13: TS1: TZ2P/B3LYP-D3(BJ)

1	C	-1.88703	2.302858	2.111697
2	C	-1.77784	0.948377	2.485777
3	C	-0.97996	0.055778	1.808463
4	C	-0.25947	0.532967	0.700699
5	C	-0.35104	1.897753	0.324785
6	C	-1.16805	2.79027	1.035532
7	C	0.559683	2.094554	-0.75182
8	O	0.859732	0.826633	-1.22622
9	C	0.587152	-0.10066	-0.25452
10	C	0.79086	-1.49258	-0.59569
11	C	0.794603	-1.86962	-1.95112
12	C	0.561731	-3.20737	-2.33457
13	C	0.301765	-4.16027	-1.40157
14	C	0.387834	-3.87307	-0.01714

15	C	0.744952	-2.55526	0.393761
16	C	1.101369	-2.41089	1.765522
17	C	0.861168	-3.42744	2.665865
18	C	0.358043	-4.67856	2.27251
19	C	0.174748	-4.89716	0.928947
20	C	0.110663	-5.74998	3.29771
21	O	0.916947	-1.0225	-2.99449
22	O	1.707134	-1.2909	2.237626
23	O	-1.19464	4.071317	0.582425
24	C	-2.00647	5.009455	1.277321
25	H	-2.54389	2.948564	2.673462
26	H	-2.35376	0.604472	3.334455
27	H	-0.92122	-0.97544	2.113094
28	H	0.510896	2.850602	-1.51838
29	H	0.538785	-3.42163	-3.39365
30	H	0.058569	-5.16955	-1.7076
31	H	1.139806	-3.24902	3.696553
32	H	-0.12128	-5.87463	0.56908
33	H	-0.17154	-6.69085	2.826953
34	H	1.001132	-5.92601	3.904466
35	H	-0.69172	-5.45937	3.979777
36	H	1.046302	-0.11722	-2.67691
37	H	2.51874	-1.10583	1.702288
38	H	-1.87356	5.957201	0.764398
39	H	-3.0588	4.719304	1.243884
40	H	-1.68828	5.108153	2.317879
41	C	2.337389	3.264848	2.416407
42	C	2.161831	3.739253	0.949115
43	C	2.330447	2.474789	0.130477
44	C	2.56037	1.384331	0.976394
45	C	2.245091	1.712373	2.395478
46	C	3.20624	2.274509	-1.08542
47	N	3.776104	1.018632	-0.90636
48	C	3.426829	0.439378	0.329843
49	H	4.322281	0.544939	-1.60827
50	O	3.368984	3.00091	-2.03635
51	O	3.826017	-0.66123	0.704869
52	O	3.630259	3.712982	2.830303
53	H	1.19923	4.219036	0.794008
54	H	2.936643	4.467637	0.713818
55	H	1.241442	1.380493	2.675337
56	H	2.934136	1.256406	3.10685
57	H	3.811134	3.370852	3.712096
58	H	1.573413	3.696408	3.06951

Table S14: TS2: TZ2P/B3LYP-D3(BJ)

1	C	-1.91982	2.286164	2.072552
2	C	-1.81871	0.931518	2.443331
3	C	-1.01982	0.038094	1.768786
4	C	-0.29213	0.516556	0.666885
5	C	-0.38271	1.881049	0.288289
6	C	-1.197	2.774293	1.000782
7	C	0.535034	2.078727	-0.78234
8	O	0.85084	0.809182	-1.24646
9	C	0.564582	-0.11619	-0.27846
10	C	0.790495	-1.50646	-0.60621
11	C	0.818016	-1.89201	-1.95934
12	C	0.607109	-3.23478	-2.33712
13	C	0.347092	-4.18466	-1.40079
14	C	0.411008	-3.88786	-0.01715
15	C	0.744683	-2.56285	0.390215
16	C	1.079591	-2.40404	1.765967
17	C	0.841176	-3.41901	2.668969
18	C	0.362336	-4.67973	2.27671
19	C	0.199806	-4.90927	0.932201
20	C	0.117997	-5.74841	3.30549
21	O	0.943824	-1.04891	-3.00534
22	O	1.659594	-1.27264	2.240782
23	O	-1.21374	4.058326	0.557802
24	C	-1.90363	5.021351	1.345413
25	H	-2.56359	2.936644	2.643244
26	H	-2.39546	0.589122	3.29195
27	H	-0.96169	-0.99268	2.07438
28	H	0.485188	2.828033	-1.55543
29	H	0.600564	-3.45645	-3.39489
30	H	0.120919	-5.199	-1.70325
31	H	1.101102	-3.22924	3.702493
32	H	-0.07765	-5.89302	0.574638
33	H	-0.15923	-6.69233	2.8379
34	H	1.008498	-5.91807	3.914095
35	H	-0.68635	-5.45855	3.985466
36	H	1.057556	-0.14051	-2.69016
37	H	2.480253	-1.08019	1.722322
38	H	-1.73652	5.978719	0.860493
39	H	-2.97521	4.812282	1.375136
40	H	-1.5033	5.043622	2.36096
41	C	2.286269	3.312121	2.376937
42	C	2.117247	3.768026	0.890844
43	C	2.302303	2.487517	0.103886
44	C	2.532475	1.417597	0.978378

45	C	2.181893	1.765756	2.384414
46	C	3.187334	2.264946	-1.10046
47	N	3.756172	1.01483	-0.89265
48	C	3.39863	0.459618	0.355843
49	H	4.294953	0.520292	-1.58564
50	O	3.360284	2.977414	-2.06147
51	O	3.799844	-0.63416	0.750375
52	H	3.283733	3.597382	2.722311
53	H	1.135095	4.21327	0.754203
54	H	2.859847	4.513109	0.604126
55	H	1.159444	1.474541	2.638909
56	H	2.834269	1.306532	3.124732
57	O	1.307612	3.865126	3.255869
58	H	1.578792	4.753856	3.503943

Table S15: TS3: TZ2P/B3LYP-D3(BJ)

1	C	-2.25914	2.909133	1.643714
2	C	-2.5286	1.562616	1.978147
3	C	-1.78845	0.514797	1.490724
4	C	-0.72438	0.816155	0.61895
5	C	-0.45728	2.162108	0.265199
6	C	-1.22153	3.220316	0.787948
7	C	0.679685	2.151944	-0.58848
8	O	0.813059	0.843972	-1.02412
9	C	0.193181	0.012983	-0.11643
10	C	0.291558	-1.40809	-0.47584
11	C	0.203686	-1.71759	-1.84279
12	C	0.345055	-3.03662	-2.31436
13	C	0.652475	-4.04599	-1.45851
14	C	0.788177	-3.8102	-0.07217
15	C	0.542077	-2.49977	0.443059
16	C	0.618188	-2.37268	1.859578
17	C	1.032794	-3.41847	2.653806
18	C	1.347276	-4.68275	2.126009
19	C	1.187701	-4.86631	0.776653
20	C	1.805854	-5.78746	3.036213
21	O	-0.06015	-0.82148	-2.82894
22	O	0.205124	-1.21476	2.417337
23	O	-0.87001	4.460089	0.375254
24	C	-1.51191	5.575164	0.977283
25	H	-2.88632	3.686077	2.053268
26	H	-3.35992	1.359449	2.640186
27	H	-2.00542	-0.50232	1.767276
28	H	0.926839	2.89989	-1.32413
29	H	0.233141	-3.20019	-3.37686

30	H	0.806791	-5.04991	-1.83231
31	H	1.076288	-3.25207	3.723296
32	H	1.37982	-5.83406	0.330474
33	H	1.968283	-6.71252	2.484714
34	H	2.741148	-5.52003	3.532966
35	H	1.069722	-5.98274	3.818921
36	H	0.068088	0.076881	-2.49934
37	H	0.838504	-0.83476	3.064026
38	H	-1.02726	6.455505	0.566761
39	H	-2.57767	5.594874	0.736991
40	H	-1.3817	5.559396	2.061625
41	N	1.624856	2.616534	2.659608
42	C	2.0429	3.243445	1.488612
43	C	2.33419	2.108815	0.544967
44	C	2.283016	0.913467	1.288075
45	C	1.851158	1.231861	2.637929
46	C	3.48858	2.033348	-0.43078
47	C	3.793374	0.51085	-0.5158
48	C	3.27819	-0.10093	0.807858
49	H	3.240566	0.077296	-1.35042
50	O	5.179751	0.218115	-0.65563
51	H	2.887913	-1.10678	0.663105
52	H	4.115683	-0.17888	1.508041
53	H	4.355672	2.54253	-0.0039
54	H	3.293469	2.478979	-1.40611
55	O	1.723251	0.522812	3.629843
56	O	2.134593	4.433215	1.314609
57	H	1.312914	3.109656	3.481173
58	H	5.441413	0.353398	-1.57149

Table S16: TS4: TZ2P/B3LYP-D3(BJ)

1	C	-2.04489	2.283443	1.982514
2	C	-1.98151	0.920832	2.343438
3	C	-1.13572	0.03059	1.726871
4	C	-0.30887	0.518474	0.698087
5	C	-0.3697	1.88048	0.325415
6	C	-1.23727	2.774069	0.97373
7	C	0.623325	2.080614	-0.67993
8	O	0.934277	0.813636	-1.15295
9	C	0.604531	-0.10396	-0.20989
10	C	0.847831	-1.49512	-0.5176
11	C	1.005002	-1.89057	-1.85949
12	C	0.7194	-3.21832	-2.25158
13	C	0.357189	-4.15924	-1.33788

14	C	0.38242	-3.87319	0.050626
15	C	0.702062	-2.55311	0.464145
16	C	0.984819	-2.37824	1.845908
17	C	0.771165	-3.39112	2.755096
18	C	0.330462	-4.66439	2.346563
19	C	0.169048	-4.89556	1.002186
20	C	0.098292	-5.73908	3.37209
21	O	1.34134	-1.07174	-2.86979
22	O	1.527831	-1.19522	2.175469
23	O	-1.20814	4.051523	0.528917
24	C	-1.94982	5.029081	1.242726
25	H	-2.73913	2.930767	2.495885
26	H	-2.63683	0.569905	3.129645
27	H	-1.12496	-1.00757	2.014316
28	H	0.593309	2.839754	-1.44476
29	H	0.775939	-3.44225	-3.30778
30	H	0.103873	-5.16131	-1.65902
31	H	0.995566	-3.20891	3.799915
32	H	-0.09908	-5.88252	0.64716
33	H	-0.16485	-6.68477	2.900433
34	H	0.989326	-5.89935	3.982796
35	H	-0.71213	-5.46263	4.050473
36	H	1.792212	-0.2752	-2.53583
37	H	1.738969	-1.06371	3.108133
38	H	-1.71984	5.979878	0.771897
39	H	-3.02358	4.836016	1.179147
40	H	-1.6456	5.058818	2.291437
41	N	1.788936	2.899116	2.367759
42	C	1.965383	3.519565	1.141922
43	C	2.347701	2.398128	0.211152
44	C	2.643006	1.276223	1.010166
45	C	2.309015	1.577867	2.389077
46	C	3.371906	2.497742	-0.90238
47	C	4.082683	1.122422	-0.91156
48	C	3.727368	0.4204	0.43278
49	O	3.620861	0.389682	-2.06204
50	H	5.161971	1.251232	-1.00987
51	H	3.435084	-0.61782	0.256188
52	H	4.595196	0.390011	1.097766
53	H	4.080762	3.29292	-0.66683
54	H	2.962294	2.715458	-1.88769
55	O	2.454887	0.952005	3.427208
56	O	1.834662	4.697643	0.905932
57	H	1.527736	3.389806	3.207965
58	H	4.135066	-0.42123	-2.13593

Table S17: TS5: TZ2P/B3LYP-D3(BJ)

1	C	-1.17746	2.707883	2.608555
2	C	-0.86181	1.467267	3.20445
3	C	-0.42984	0.386672	2.4721
4	C	-0.31975	0.554391	1.081697
5	C	-0.60344	1.802917	0.473485
6	C	-1.03598	2.892753	1.244501
7	C	-0.25932	1.66747	-0.90067
8	O	-0.19347	0.300789	-1.15155
9	C	0.014445	-0.34101	0.039202
10	C	0.150112	-1.79475	0.025175
11	C	1.186378	-2.33894	0.777347
12	C	1.481157	-3.71204	0.732563
13	C	0.744555	-4.54339	-0.0539
14	C	-0.36347	-4.06694	-0.79263
15	C	-0.70079	-2.67521	-0.74608
16	C	-1.91623	-2.31282	-1.4112
17	C	-2.6567	-3.24899	-2.09558
18	C	-2.2884	-4.6021	-2.17138
19	C	-1.14981	-4.99071	-1.5159
20	C	-3.14085	-5.57152	-2.94094
21	O	1.976753	-1.53945	1.559394
22	O	-2.47678	-1.0686	-1.35096
23	O	-1.28027	4.045418	0.572037
24	C	-1.71881	5.175294	1.316979
25	H	-1.53064	3.510808	3.236799
26	H	-0.97895	1.372275	4.275709
27	H	-0.19091	-0.55789	2.93541
28	H	-0.61793	2.252426	-1.73175
29	H	2.316888	-4.07011	1.317233
30	H	0.982529	-5.59784	-0.10815
31	H	-3.56364	-2.90206	-2.57354
32	H	-0.84187	-6.02867	-1.52166
33	H	-2.7344	-6.58086	-2.89274
34	H	-4.15919	-5.59483	-2.5471
35	H	-3.2087	-5.28237	-3.99195
36	H	2.789468	-1.36293	1.037788
37	H	-1.79349	-0.38995	-1.4012
38	H	-1.84555	5.977186	0.596095
39	H	-2.67119	4.973312	1.811769
40	H	-0.97408	5.466289	2.061332
41	C	2.383988	3.081166	1.130328
42	C	1.9724	3.37468	-0.33565
43	C	1.734208	2.006702	-0.93342

44	C	2.311777	1.026861	-0.11622
45	C	2.733058	1.56127	1.211182
46	C	1.986597	1.589517	-2.36424
47	N	2.613345	0.350137	-2.26871
48	C	2.880359	-0.01877	-0.94065
49	H	2.814546	-0.23759	-3.06248
50	O	1.704614	2.165011	-3.38654
51	O	3.507356	-1.0205	-0.6193
52	O	3.481112	3.934643	1.453287
53	H	1.122067	4.048599	-0.40116
54	H	2.815128	3.853112	-0.83802
55	H	2.229149	1.075467	2.049219
56	H	3.808785	1.437293	1.359844
57	H	3.662059	3.867981	2.396437
58	H	1.553317	3.296461	1.802755

Table S18: TS6: TZ2P/B3LYP-D3(BJ)

1	C	-1.15175	2.62672380	73296853
2	C	-0.87127	1.36403	3.294989
3	C	-0.49835	0.284717	2.529944
4	C	-0.41213	0.478576	1.141364
5	C	-0.6709	1.747887	0.563687
6	C	-1.042	2.834749	1.37083
7	C	-0.35679	1.6336	-0.8198
8	O	-0.3054	0.269979	-1.09688
9	C	-0.10809	-0.39915	0.078526
10	C	0.0591	-1.84548	0.029394
11	C	1.094449	-2.38291	0.789995
12	C	1.436869	-3.74283	0.699923
13	C	0.747225	-4.56761	-0.13459
14	C	-0.35798	-4.10055	-0.88388
15	C	-0.74102	-2.72333	-0.79644
16	C	-1.94895	-2.37312	-1.4805
17	C	-2.64129	-3.30554	-2.21868
18	C	-2.22913	-4.6433	-2.33174
19	C	-1.09604	-5.02099	-1.66007
20	C	-3.03111	-5.61028	-3.15684
21	O	1.836098	-1.58703	1.620168
22	O	-2.54878	-1.14961	-1.38956
23	O	-1.27312	4.009438	0.730152
24	C	-1.54282	5.158116	1.524822
25	H	-1.44411	3.432344	3.387788
26	H	-0.95708	1.252581	4.367446
27	H	-0.27327	-0.67432	2.968784

28	H	-0.72775	2.234114	-1.6342
29	H	2.269891	-4.09586	1.2915
30	H	1.021219	-5.6112	-0.22039
31	H	-3.54576	-2.96916	-2.70875
32	H	-0.75592	-6.0483	-1.69451
33	H	-2.59085	-6.60636	-3.13828
34	H	-4.05597	-5.68371	-2.78661
35	H	-3.08736	-5.28281	-4.19721
36	H	2.671802	-1.39734	1.142756
37	H	-1.88622	-0.44868	-1.39389
38	H	-1.64028	5.986188	0.828967
39	H	-2.47405	5.040804	2.083132
40	H	-0.72033	5.34962	2.217302
41	C	2.73458	3.104855	0.946731
42	C	1.875225	3.362105	-0.33348
43	C	1.641716	1.972627	-0.89234
44	C	2.228566	1.016056	-0.0525
45	C	2.607995	1.593879	1.267371
46	C	1.885593	1.516123	-2.31213
47	N	2.512437	0.281415	-2.18641
48	C	2.792519	-0.04876	-0.84693
49	H	2.704766	-0.33136	-2.96277
50	O	1.602142	2.06921	-3.34741
51	O	3.426702	-1.04105	-0.50756
52	H	3.781066	3.326263	0.719115
53	H	0.95557	3.87255	-0.05989
54	H	2.395878	3.990593	-1.05644
55	H	1.827294	1.449629	2.02009
56	H	3.527914	1.178236	1.675874
57	O	2.323826	3.873969	2.074481
58	H	2.699459	4.75664	2.004914

Table S19: TS7: TZ2P/B3LYP-D3(BJ)

1	C	-1.18234	2.95639	2.788341
2	C	-0.91339	1.740062	3.446738
3	C	-0.57225	0.593906	2.771308
4	C	-0.48614	0.660006	1.366279
5	C	-0.75229	1.884938	0.690713
6	C	-1.10286	3.047376	1.411475
7	C	-0.49814	1.644869	-0.68432
8	O	-0.4047	0.299811	-0.85492
9	C	-0.21273	-0.29145	0.353675
10	C	0.015366	-1.72688	0.383445
11	C	0.925836	-2.27915	1.291032

12	C	1.314012	-3.63522	1.182174
13	C	0.880823	-4.40391	0.145641
14	C	-0.08347	-3.91167	-0.76895
15	C	-0.5947	-2.59288	-0.59986
16	C	-1.75039	-2.27236	-1.37225
17	C	-2.19643	-3.11598	-2.3708
18	C	-1.58057	-4.34957	-2.62423
19	C	-0.55983	-4.75314	-1.79732
20	C	-2.0863	-5.2195	-3.74146
21	O	1.515704	-1.62297	2.30191
22	O	-2.53451	-1.19047	-1.14266
23	O	-1.3525	4.149658	0.674807
24	C	-1.46611	5.416921	1.298022
25	H	-1.45936	3.818002	3.382004
26	H	-0.9906	1.719287	4.528142
27	H	-0.37939	-0.3313	3.297386
28	H	-0.85718	2.207407	-1.53357
29	H	2.023742	-4.00865	1.910138
30	H	1.248157	-5.41646	0.020935
31	H	-3.06976	-2.81479	-2.93749
32	H	-0.11476	-5.73593	-1.90666
33	H	-1.5388	-6.16085	-3.78174
34	H	-3.14706	-5.43605	-3.60338
35	H	-1.97064	-4.70641	-4.69792
36	H	1.706706	-0.66267	2.216919
37	H	-2.19147	-0.6223	-0.44261
38	H	-1.53571	6.145775	0.489531
39	H	-2.36563	5.477151	1.921653
40	H	-0.58265	5.632896	1.909145
41	N	1.991592	2.817105	1.174126
42	C	1.64899	3.217268	-0.1113
43	C	1.585602	1.936728	-0.87984
44	C	2.111204	0.932807	-0.07471
45	C	2.274664	1.448355	1.270263
46	C	1.842687	1.636341	-2.33237
47	C	2.237521	0.129683	-2.31618
48	C	2.674009	-0.20468	-0.86794
49	O	1.459034	4.35066	-0.45609
50	O	2.525991	0.895139	2.32278
51	H	2.05379	3.446453	1.956755
52	H	2.689038	2.234186	-2.6855
53	H	0.993685	1.827346	-2.98984
54	H	2.3226	-1.19149	-0.55819
55	H	3.767625	-0.20747	-0.80929
56	O	3.313859	-0.14471	-3.18142

57	H	1.359972	-0.4809	-2.59094
58	H	3.048585	-0.04666	-4.10274

Table S20: TS8: TZ2P/B3LYP-D3(BJ)

1	C	-1.45733	2.81563	2.482787
2	C	-1.12638	1.64198	3.192282
3	C	-0.60786	0.526278	2.575225
4	C	-0.41396	0.596923	1.18642
5	C	-0.72543	1.770941	0.463226
6	C	-1.2574	2.896479	1.114143
7	C	-0.27816	1.547305	-0.87561
8	O	-0.13638	0.183684	-1.01411
9	C	0.097281	-0.33773	0.236316
10	C	0.263637	-1.78761	0.29535
11	C	1.280303	-2.38046	1.043758
12	C	1.586118	-3.7534	0.853302
13	C	0.941107	-4.49349	-0.08326
14	C	-0.15254	-3.96706	-0.81613
15	C	-0.56013	-2.62708	-0.55514
16	C	-1.82536	-2.2497	-1.09624
17	C	-2.47738	-3.05925	-1.9993
18	C	-1.98378	-4.32178	-2.36498
19	C	-0.85085	-4.77613	-1.73481
20	C	-2.72416	-5.15464	-3.37382
21	O	2.091451	-1.78633	1.930651
22	O	-2.5059	-1.12066	-0.73247
23	O	-1.53364	3.959471	0.330316
24	C	-1.91251	5.179408	0.955858
25	H	-1.87908	3.649005	3.023298
26	H	-1.30043	1.625343	4.259763
27	H	-0.358	-0.36599	3.129696
28	H	-0.6132	2.061888	-1.76065
29	H	2.400546	-4.15793	1.437391
30	H	1.238436	-5.51839	-0.26454
31	H	-3.42722	-2.71148	-2.38474
32	H	-0.48393	-5.77738	-1.92173
33	H	-2.29065	-6.15022	-3.45894
34	H	-3.77594	-5.26215	-3.1011
35	H	-2.69256	-4.68754	-4.36088
36	H	2.118114	-0.80607	2.000374
37	H	-2.12881	-0.72602	0.059635
38	H	-2.00907	5.906086	0.155455
39	H	-2.86766	5.075586	1.475562
40	H	-1.14309	5.508529	1.657058

41	N	2.099189	2.72856	1.298795
42	C	1.767372	3.167662	0.012158
43	C	1.722465	1.917647	-0.81592
44	C	2.221679	0.864748	-0.01679
45	C	2.397137	1.368508	1.343995
46	C	2.245953	1.774605	-2.22936
47	C	2.769105	0.309791	-2.29982
48	C	3.035831	-0.1096	-0.82216
49	O	1.56326	4.313192	-0.30134
50	O	2.696843	0.771761	2.368126
51	H	2.044041	3.309276	2.121085
52	H	3.064303	2.483143	-2.37688
53	H	1.51621	1.95954	-3.01591
54	H	2.782843	-1.15979	-0.68116
55	H	4.09524	-0.00402	-0.57236
56	H	3.684014	0.257837	-2.88612
57	O	1.871429	-0.56375	-2.96507
58	H	1.040408	-0.58577	-2.47402

Table S21: I1: TZ2P/B3LYP-D3(BJ)

1	C	-1.49864	2.380417	2.914881
2	C	-1.01202	1.175478	3.413624
3	C	-0.37306	0.242088	2.598334
4	C	-0.25975	0.548268	1.255871
5	C	-0.7529	1.744851	0.739037
6	C	-1.36145	2.684019	1.555133
7	C	-0.45203	1.734037	-0.7386
8	O	-0.47724	0.325005	-0.99805
9	C	0.402383	-0.1523	0.070873
10	C	0.369902	-1.68621	0.045424
11	C	-0.36364	-2.35528	-0.93674
12	C	-0.82109	-3.68319	-0.73461
13	C	-0.61344	-4.32899	0.43887
14	C	0.223016	-3.75815	1.426912
15	C	0.821399	-2.4906	1.161636
16	C	1.914762	-2.15984	2.006599
17	C	2.167305	-2.84724	3.169994
18	C	1.423868	-3.98685	3.528267
19	C	0.504118	-4.45489	2.622615
20	C	1.709509	-4.70045	4.820269
21	O	-0.74754	-1.85825	-2.13786
22	O	2.784707	-1.17225	1.656557
23	O	-1.77903	3.83639	0.963608
24	C	-2.43918	4.804056	1.769485
25	H	-1.98838	3.069792	3.585435

26	H	-1.13735	0.961594	4.466761
27	H	0.006752	-0.68171	3.004691
28	H	-1.13261	2.262773	-1.39609
29	H	-1.4103	-4.11805	-1.52965
30	H	-1.04943	-5.30339	0.617648
31	H	3.011692	-2.53063	3.768861
32	H	-0.01081	-5.39063	2.80165
33	H	1.153158	-5.63464	4.887042
34	H	2.772756	-4.92943	4.917957
35	H	1.433478	-4.08209	5.6779
36	H	-0.6563	-0.89173	-2.11856
37	H	2.974636	-1.21943	0.698206
38	H	-2.68752	5.626479	1.105185
39	H	-3.35466	4.396615	2.2042
40	H	-1.78622	5.164741	2.567813
41	C	2.899594	2.820324	0.334709
42	C	1.63319	3.28548	-0.37346
43	C	1.047717	2.00143	-0.9736
44	C	1.704715	0.786218	-0.25119
45	C	2.560474	1.4246	0.862809
46	C	1.408046	1.796506	-2.4443
47	N	2.233133	0.675574	-2.52232
48	C	2.525964	0.097037	-1.31191
49	H	2.641607	0.365167	-3.39254
50	O	1.054648	2.457929	-3.38418
51	O	3.282394	-0.83573	-1.12928
52	O	3.923781	2.755947	-0.6673
53	H	0.935836	3.705398	0.349667
54	H	1.842559	4.042215	-1.12595
55	H	1.981756	1.499695	1.78112
56	H	3.458141	0.864982	1.097145
57	H	4.704925	2.336874	-0.2905
58	H	3.203595	3.498726	1.136583

Table S22: I2: TZ2P/B3LYP-D3(BJ)

1	C	-1.50763	2.383149	2.917877
2	C	-1.00795	1.185663	3.420846
3	C	-0.3677	0.251503	2.60774
4	C	-0.26653	0.548517	1.262396
5	C	-0.77262	1.73809	0.742143
6	C	-1.38173	2.678801	1.555799
7	C	-0.47355	1.726741	-0.73441
8	O	-0.48434	0.318115	-0.99343
9	C	0.395965	-0.15226	0.078457
10	C	0.373281	-1.68689	0.049379

11	C	-0.34271	-2.35944	-0.94344
12	C	-0.79132	-3.69151	-0.75134
13	C	-0.58851	-4.34172	0.420751
14	C	0.230597	-3.76688	1.420608
15	C	0.817017	-2.49124	1.168536
16	C	1.895164	-2.15051	2.027913
17	C	2.146906	-2.8421	3.188448
18	C	1.41398	-3.99314	3.532459
19	C	0.508252	-4.46656	2.615599
20	C	1.696278	-4.71063	4.822999
21	O	-0.71912	-1.86029	-2.14714
22	O	2.754592	-1.14934	1.688718
23	O	-1.80945	3.824855	0.958961
24	C	-2.44511	4.806063	1.768183
25	H	-1.99364	3.07516	3.588307
26	H	-1.12106	0.979352	4.476807
27	H	0.023782	-0.66478	3.019353
28	H	-1.15808	2.248757	-1.39313
29	H	-1.36957	-4.12757	-1.55371
30	H	-1.01717	-5.32101	0.590286
31	H	2.980858	-2.5176	3.797477
32	H	0.002955	-5.40942	2.784606
33	H	1.149454	-5.65105	4.880304
34	H	2.761114	-4.92855	4.928417
35	H	1.406599	-4.09953	5.681276
36	H	-0.64947	-0.89232	-2.11643
37	H	2.95766	-1.20274	0.73563
38	H	-2.69722	5.625406	1.101392
39	H	-3.35698	4.412159	2.222617
40	H	-1.7737	5.166013	2.551196
41	C	2.887767	2.830323	0.319906
42	C	1.601323	3.292775	-0.37432
43	C	1.024442	2.007357	-0.97116
44	C	1.695088	0.793904	-0.24858
45	C	2.561245	1.433341	0.851595
46	C	1.385921	1.808566	-2.44178
47	N	2.246546	0.713456	-2.5189
48	C	2.513051	0.104877	-1.3113
49	H	2.626102	0.383673	-3.39582
50	O	1.023817	2.463857	-3.38347
51	O	3.261155	-0.83395	-1.13937
52	O	3.299992	3.656396	1.398505
53	H	0.923921	3.687273	0.382098
54	H	1.759623	4.064865	-1.12581
55	H	1.990872	1.532649	1.772783

56	H	3.461239	0.879619	1.081578
57	H	3.687412	4.465723	1.050926
58	H	3.697055	2.756298	-0.41727

Table S23: **I3**: TZ2P/B3LYP-D3(BJ)

1	C	-1.6148	2.422719	2.665797
2	C	-1.12971	1.233899	3.20687
3	C	-0.45619	0.291772	2.431787
4	C	-0.29865	0.581146	1.089687
5	C	-0.77566	1.760848	0.535072
6	C	-1.43248	2.706731	1.307075
7	C	-0.31496	1.764727	-0.89772
8	O	-0.29031	0.364334	-1.20039
9	C	0.437911	-0.14741	-0.0373
10	C	0.229827	-1.67555	0.041863
11	C	-0.86806	-2.23852	-0.6207
12	C	-1.45755	-3.45357	-0.18306
13	C	-1.04622	-4.06942	0.950084
14	C	0.101267	-3.60321	1.632162
15	C	0.811185	-2.48482	1.097665
16	C	2.113063	-2.2997	1.631644
17	C	2.533792	-2.95524	2.766694
18	C	1.736807	-3.92256	3.403498
19	C	0.558579	-4.26918	2.789814
20	C	2.218252	-4.59896	4.656638
21	O	-1.53974	-1.71777	-1.67648
22	O	2.997014	-1.5554	0.915526
23	O	-1.84509	3.836892	0.679106
24	C	-2.43244	4.8664	1.463098
25	H	-2.13546	3.118616	3.305712
26	H	-1.28736	1.041252	4.25976
27	H	-0.08011	-0.62479	2.860781
28	H	-0.9099	2.31113	-1.62029
29	H	-2.3064	-3.80798	-0.75081
30	H	-1.56586	-4.94102	1.326686
31	H	3.539774	-2.76121	3.118178
32	H	-0.039	-5.08546	3.177023
33	H	1.541844	-5.39735	4.959723
34	H	3.211842	-5.02985	4.515562
35	H	2.290961	-3.88678	5.482037
36	H	-1.23326	-0.80467	-1.80835
37	H	3.275106	-0.76944	1.43345
38	H	-2.6351	5.684072	0.777929
39	H	-3.36721	4.53221	1.919454
40	H	-1.74578	5.20575	2.24188

41	N	2.253017	2.686769	1.007493
42	C	1.638147	3.20643	-0.11518
43	C	1.204305	2.028186	-0.957
44	C	1.795419	0.735274	-0.26431
45	C	2.467186	1.330237	0.992749
46	C	1.773713	2.060658	-2.37875
47	C	3.093687	1.302005	-2.25719
48	C	2.776003	0.154506	-1.30521
49	O	3.124233	0.793508	1.86429
50	H	3.674614	-0.25955	-0.86615
51	O	1.485897	4.379033	-0.34608
52	H	2.657239	3.2693	1.727026
53	H	2.271664	-0.64921	-1.84016
54	H	1.891464	3.079881	-2.74665
55	H	1.105546	1.524417	-3.05299
56	O	4.088487	2.104972	-1.60599
57	H	3.468607	0.946454	-3.22025
58	H	4.245258	2.899127	-2.12762

Table S24: I4: TZ2P/B3LYP-D3(BJ)

1	C	-1.5779	2.411147	2.712003
2	C	-1.08872	1.220613	3.245352
3	C	-0.42364	0.279219	2.462069
4	C	-0.27882	0.570641	1.118682
5	C	-0.76061	1.752925	0.571429
6	C	-1.40955	2.697807	1.351928
7	C	-0.32439	1.757341	-0.86953
8	O	-0.30124	0.358774	-1.1685
9	C	0.444935	-0.15321	-0.01891
10	C	0.253203	-1.68327	0.041018
11	C	-0.80117	-2.26458	-0.67327
12	C	-1.39125	-3.48862	-0.25998
13	C	-1.02142	-4.09784	0.891151
14	C	0.092283	-3.61795	1.619088
15	C	0.805802	-2.48991	1.113477
16	C	2.087315	-2.2901	1.688001
17	C	2.479445	-2.94299	2.834678
18	C	1.671675	-3.92007	3.443409
19	C	0.517718	-4.27917	2.791398
20	C	2.119408	-4.59384	4.710407
21	O	-1.42396	-1.76045	-1.76672
22	O	2.982351	-1.54064	0.991844
23	O	-1.82993	3.82736	0.729653
24	C	-2.40885	4.857543	1.519665

25	H	-2.09291	3.105578	3.358047
26	H	-1.23754	1.025093	4.298985
27	H	-0.04576	-0.63868	2.886051
28	H	-0.93202	2.302715	-1.58209
29	H	-2.21044	-3.8544	-0.86293
30	H	-1.54699	-4.97439	1.247462
31	H	3.472499	-2.74254	3.217977
32	H	-0.0816	-5.10374	3.157651
33	H	1.446806	-5.40544	4.985533
34	H	3.124916	-5.00644	4.60381
35	H	2.149348	-3.88464	5.541071
36	H	-1.14651	-0.83595	-1.87517
37	H	3.255893	-0.75729	1.513375
38	H	-2.61679	5.675404	0.836409
39	H	-3.33951	4.524028	1.984673
40	H	-1.7144	5.195731	2.291924
41	N	2.298322	2.711821	0.972721
42	C	1.620293	3.217047	-0.12292
43	C	1.193117	2.028232	-0.95297
44	C	1.797438	0.738934	-0.25154
45	C	2.472896	1.348463	0.998518
46	C	1.753646	2.059068	-2.3745
47	C	3.083009	1.303011	-2.2605
48	C	2.782041	0.160115	-1.2811
49	O	3.119423	0.826104	1.88671
50	H	3.674693	-0.26174	-0.83637
51	O	1.429607	4.385304	-0.34067
52	H	2.672476	3.299801	1.704992
53	H	2.276965	-0.65041	-1.81187
54	H	1.862624	3.072499	-2.75477
55	H	1.086852	1.506433	-3.03914
56	O	3.639837	0.887407	-3.49217
57	H	3.056703	0.231976	-3.89283
58	H	3.837851	1.965443	-1.82944

Table S25: I5: TZ2P/B3LYP-D3(BJ)

1	C	-2.09052	2.201949	2.254028
2	C	-1.68119	0.988556	2.802814
3	C	-0.81229	0.133827	2.127208
4	C	-0.38642	0.53653	0.878437
5	C	-0.78514	1.738382	0.313584
6	C	-1.63328	2.599485	0.991244
7	C	-0.09249	1.818256	-1.01997
8	O	-0.00433	0.415547	-1.3905

9	C	0.541939	-0.11852	-0.133
10	C	0.535439	-1.64145	-0.12223
11	C	1.571595	-2.2846	0.533624
12	C	1.696666	-3.68541	0.568408
13	C	0.766418	-4.4637	-0.03615
14	C	-0.37603	-3.89132	-0.63944
15	C	-0.54468	-2.46102	-0.66609
16	C	-1.82289	-2.0327	-1.179
17	C	-2.75065	-2.94091	-1.64431
18	C	-2.53883	-4.32459	-1.65334
19	C	-1.35105	-4.77807	-1.14779
20	C	-3.59133	-5.2506	-2.19472
21	O	2.534835	-1.59015	1.223874
22	O	-2.25565	-0.74831	-1.19578
23	O	-1.95092	3.767758	0.373841
24	C	-2.86773	4.642308	1.019877
25	H	-2.77291	2.826075	2.810306
26	H	-2.06042	0.705686	3.775702
27	H	-0.50193	-0.81096	2.549709
28	H	-0.56494	2.393143	-1.80795
29	H	2.552631	-4.10001	1.08187
30	H	0.861724	-5.54177	-0.03426
31	H	-3.68185	-2.52801	-2.01022
32	H	-1.14177	-5.83942	-1.10415
33	H	-3.28978	-6.29288	-2.09682
34	H	-4.53856	-5.11825	-1.66755
35	H	-3.78069	-5.05017	-3.25174
36	H	3.23346	-1.34665	0.578977
37	H	-1.48535	-0.16565	-1.34142
38	H	-2.99095	5.490523	0.353092
39	H	-3.83298	4.155206	1.174148
40	H	-2.47516	4.987008	1.979429
41	C	2.894351	2.692213	1.009379
42	C	1.789547	3.265871	0.1273
43	C	1.403544	2.102116	-0.79439
44	C	1.895499	0.77633	-0.11055
45	C	2.491914	1.232109	1.235058
46	C	2.112725	2.147195	-2.14084
47	N	2.93572	1.032669	-2.21237
48	C	2.966314	0.266262	-1.0796
49	H	3.570573	0.876736	-2.98252
50	O	1.996047	2.978991	-3.00114
51	O	3.759285	-0.63915	-0.90194
52	O	4.106363	2.780584	0.247857
53	H	0.937346	3.548749	0.742805

54	H	2.123071	4.142259	-0.42347
55	H	1.731826	1.170225	2.011431
56	H	3.331707	0.627323	1.561328
57	H	4.810177	2.325583	0.722497
58	H	3.00213	3.239845	1.949447

Table S26: I6: TZ2P/B3LYP-D3(BJ)

1	C	-2.08377	2.248297	2.265019
2	C	-1.6703	1.043244	2.828273
3	C	-0.81285	0.174839	2.155795
4	C	-0.40203	0.556071	0.895675
5	C	-0.80408	1.750281	0.316805
6	C	-1.64059	2.624966	0.991244
7	C	-0.11843	1.81363	-1.02045
8	O	-0.02748	0.407795	-1.3743
9	C	0.520021	-0.11181	-0.11023
10	C	0.517559	-1.63425	-0.09392
11	C	1.55072	-2.26938	0.572377
12	C	1.697736	-3.66794	0.59283
13	C	0.788811	-4.45229	-0.03667
14	C	-0.35506	-3.88946	-0.64647
15	C	-0.54524	-2.46179	-0.65683
16	C	-1.82553	-2.04474	-1.1725
17	C	-2.73634	-2.95967	-1.65717
18	C	-2.50312	-4.33998	-1.68388
19	C	-1.31234	-4.78309	-1.1761
20	C	-3.538	-5.27427	-2.24468
21	O	2.489965	-1.56092	1.281937
22	O	-2.27692	-0.76618	-1.16823
23	O	-1.95978	3.785948	0.360551
24	C	-2.84972	4.682373	1.014004
25	H	-2.75427	2.884807	2.821543
26	H	-2.03465	0.779279	3.812006
27	H	-0.49497	-0.76095	2.592235
28	H	-0.59399	2.378122	-1.81408
29	H	2.551638	-4.07675	1.114421
30	H	0.901259	-5.52863	-0.04815
31	H	-3.67217	-2.55625	-2.02183
32	H	-1.08847	-5.84188	-1.14435
33	H	-3.22283	-6.3133	-2.15563
34	H	-4.4914	-5.16085	-1.72419
35	H	-3.72113	-5.06471	-3.301
36	H	3.21611	-1.34808	0.662077
37	H	-1.51495	-0.17264	-1.30871

38	H	-2.9747	5.52373	0.338802
39	H	-3.81881	4.212143	1.19475
40	H	-2.43159	5.032909	1.960454
41	C	2.892331	2.711474	0.973709
42	C	1.768483	3.277066	0.093851
43	C	1.378546	2.101949	-0.80549
44	C	1.875728	0.782234	-0.10152
45	C	2.489385	1.256748	1.225574
46	C	2.086549	2.129378	-2.15213
47	N	2.936832	1.033844	-2.1954
48	C	2.940295	0.252662	-1.06565
49	H	3.544912	0.845608	-2.98056
50	O	1.967096	2.946002	-3.02775
51	O	3.720527	-0.66125	-0.89043
52	H	3.839425	2.736309	0.420361
53	H	0.933188	3.547015	0.738633
54	H	2.058861	4.15706	-0.47797
55	H	1.73634	1.23702	2.011162
56	H	3.326687	0.656581	1.559043
57	O	3.049804	3.377093	2.217536
58	H	3.495144	4.218311	2.076684

Table S27: I7: TZ2P/B3LYP-D3(BJ)

1	C	-1.99135	2.35414	2.240679
2	C	-1.57747	1.15379	2.813759
3	C	-0.77217	0.24905	2.124558
4	C	-0.40904	0.59109	0.839209
5	C	-0.809	1.783891	0.252931
6	C	-1.6017	2.690569	0.937793
7	C	-0.16491	1.814353	-1.10983
8	O	-0.10637	0.404386	-1.43934
9	C	0.483909	-0.10057	-0.18526
10	C	0.538257	-1.61824	-0.19306
11	C	1.65288	-2.27758	0.320681
12	C	1.842404	-3.66801	0.137239
13	C	0.90052	-4.41952	-0.47379
14	C	-0.33032	-3.85182	-0.8776
15	C	-0.55136	-2.44073	-0.72687
16	C	-1.88358	-2.02223	-1.07644
17	C	-2.82913	-2.92008	-1.52584
18	C	-2.57735	-4.28758	-1.68669
19	C	-1.32239	-4.72962	-1.36522
20	C	-3.65521	-5.20847	-2.18559
21	O	2.690407	-1.72495	0.986614

22	O	-2.34887	-0.75114	-0.94025
23	O	-1.9394	3.83219	0.287399
24	C	-2.71997	4.796937	0.980363
25	H	-2.62443	3.016975	2.810304
26	H	-1.90275	0.920676	3.818808
27	H	-0.45907	-0.68383	2.570698
28	H	-0.66567	2.372527	-1.89235
29	H	2.764254	-4.08928	0.512392
30	H	1.051908	-5.48148	-0.61808
31	H	-3.80556	-2.51249	-1.75422
32	H	-1.07035	-5.77904	-1.45365
33	H	-3.29328	-6.23277	-2.26739
34	H	-4.5158	-5.20503	-1.51274
35	H	-4.01434	-4.89578	-3.16864
36	H	2.529544	-0.882	1.4642
37	H	-1.63059	-0.14611	-1.19684
38	H	-2.8466	5.627452	0.29232
39	H	-3.69854	4.393048	1.250029
40	H	-2.20664	5.144625	1.879735
41	N	2.151652	2.661058	1.155575
42	C	1.684067	3.23062	-0.02188
43	C	1.347392	2.08703	-0.96164
44	C	1.830034	0.78605	-0.23612
45	C	2.274506	1.298301	1.131675
46	C	2.108398	2.156071	-2.28958
47	C	3.406735	1.403569	-1.99948
48	C	2.965279	0.233249	-1.12792
49	H	3.913365	1.069544	-2.90838
50	O	1.57177	4.41168	-0.22117
51	O	2.653542	0.638339	2.07976
52	H	2.416965	3.210245	1.960843
53	H	2.265711	3.182903	-2.61865
54	H	1.544497	1.626594	-3.05793
55	H	2.555452	-0.55827	-1.75359
56	H	3.790204	-0.18104	-0.55922
57	O	4.29587	2.195696	-1.20102
58	H	4.532185	2.993196	-1.68649

Table S28: I8: TZ2P/B3LYP-D3(BJ)

1	C	-2.00029	2.345416	2.267509
2	C	-1.58077	1.146937	2.840414
3	C	-0.77928	0.24143	2.147807
4	C	-0.42611	0.580677	0.858647
5	C	-0.8318	1.77181	0.272236

6	C	-1.62078	2.679534	0.960922
7	C	-0.19948	1.798685	-1.09649
8	O	-0.14017	0.388824	-1.42148
9	C	0.457164	-0.11443	-0.1722
10	C	0.507531	-1.63303	-0.17869
11	C	1.62372	-2.29671	0.32429
12	C	1.807013	-3.6874	0.142406
13	C	0.857531	-4.43612	-0.46061
14	C	-0.37347	-3.8638	-0.85658
15	C	-0.58857	-2.45173	-0.70451
16	C	-1.92176	-2.02899	-1.04445
17	C	-2.87258	-2.92296	-1.48994
18	C	-2.62599	-4.29089	-1.65499
19	C	-1.3712	-4.73765	-1.33962
20	C	-3.70686	-5.20611	-2.15771
21	O	2.669978	-1.74544	0.982285
22	O	-2.38225	-0.75679	-0.90142
23	O	-1.96568	3.817605	0.309941
24	C	-2.74707	4.781629	1.003387
25	H	-2.63126	3.00827	2.839433
26	H	-1.8996	0.915269	3.847878
27	H	-0.46393	-0.69103	2.593254
28	H	-0.70478	2.353684	-1.87807
29	H	2.729912	-4.11197	0.511183
30	H	1.003862	-5.49866	-0.60543
31	H	-3.84896	-2.51192	-1.71212
32	H	-1.12334	-5.78788	-1.42999
33	H	-3.36537	-6.24011	-2.19116
34	H	-4.58995	-5.15952	-1.51682
35	H	-4.02389	-4.92164	-3.1637
36	H	2.504451	-0.91014	1.467159
37	H	-1.66576	-0.15424	-1.16809
38	H	-2.88287	5.607647	0.311812
39	H	-3.72132	4.373374	1.281743
40	H	-2.22942	5.136824	1.897332
41	N	2.176655	2.648045	1.145198
42	C	1.635316	3.219128	-0.00456
43	C	1.311257	2.079007	-0.95281
44	C	1.802889	0.771064	-0.23205
45	C	2.263856	1.279972	1.131126
46	C	2.075002	2.17128	-2.27415
47	C	3.37194	1.418778	-1.99609
48	C	2.929192	0.228133	-1.13334
49	O	3.983252	1.051534	-3.22168
50	O	1.479594	4.398457	-0.17507

51	O	2.645743	0.623429	2.080254
52	H	2.409813	3.191258	1.965403
53	H	2.226934	3.199888	-2.59362
54	H	1.530365	1.635484	-3.05197
55	H	2.510055	-0.53415	-1.79002
56	H	3.738716	-0.22428	-0.56927
57	H	4.051564	2.060578	-1.42066
58	H	4.838584	0.646987	-3.04449

Table S29: P1 (1): TZ2P/B3LYP-D3(BJ)

1	O	0.039031	1.76209	0.902039
2	N	-0.35251	1.094381	-1.26122
3	O	0.50569	-1.83746	-0.53282
4	C	0.420956	1.302527	-0.15433
5	C	1.856455	0.893494	-0.48216
6	C	1.797525	0.430102	-1.97307
7	C	2.253272	-1.0521	-1.95094
8	C	1.907983	-1.55468	-0.5596
9	C	2.264847	-0.36732	0.323768
10	H	6.177483	3.590407	2.457253
11	H	6.672023	4.374766	0.939235
12	C	0.306801	0.496565	-2.33104
13	C	2.638795	1.264191	-2.99555
14	C	1.876351	2.347062	-3.79473
15	C	1.882133	1.910246	-5.12868
16	C	2.561039	0.699509	-5.25121
17	O	3.039372	0.303183	-4.03579
18	C	2.690158	0.072321	-6.4552
19	C	2.099066	0.696127	-7.59582
20	C	2.225197	0.005275	-8.92732
21	C	1.424772	1.893524	-7.48188
22	C	1.2942	2.542643	-6.22587
23	C	0.635397	3.757965	-5.92058
24	C	0.618491	4.219825	-4.63007
25	C	1.231733	3.542491	-3.53333
26	O	1.082752	4.167796	-2.34832
27	C	3.926353	1.783367	-2.3683
28	C	5.036643	1.960265	-3.19697
29	C	6.21791	2.447942	-2.67417
30	C	6.326977	2.764437	-1.32359
31	C	5.224101	2.596559	-0.49687
32	O	5.226979	2.882753	0.833427
33	C	6.406453	3.424824	1.408508
34	C	4.010756	2.10547	-1.01705
35	C	2.821296	2.036688	-0.10598
36	O	2.202094	3.335875	-0.12346
37	H	1.794642	-0.38654	1.307097
38	O	-0.22498	0.111686	-3.33474
39	H	1.595316	3.782985	-1.60584
40	H	4.967515	1.707858	-4.24306
41	H	7.245417	2.729727	1.324131
42	H	7.259974	3.141618	-0.93527
43	H	7.074738	2.585235	-3.32036

44	H	0.254807	-2.11084	0.355507
45	H	3.212405	-0.86968	-6.55264
46	H	3.273609	-0.14649	-9.19485
47	H	1.754326	0.584695	-9.72022
48	H	1.75352	-0.98007	-8.90269
49	H	0.983218	2.345034	-8.3613
50	H	0.14539	4.325336	-6.70154
51	H	0.120569	5.149474	-4.38864
52	H	-1.351	1.245776	-1.26402
53	H	2.47753	-2.45042	-0.2983
54	H	3.346762	-0.34751	0.471654
55	H	3.33102	-1.09936	-2.09274
56	H	1.781822	-1.62969	-2.74014
57	H	1.499708	3.335721	0.5438
58	H	3.159599	1.860305	0.912227

Table S30: P2: TZ2P/B3LYP-D3(BJ)

1	O	-5.5E-05	1.944739	0.777188
2	N	-0.356	1.048512	-1.30782
3	H	1.531041	3.38553	0.523028
4	C	0.407327	1.390615	-0.22054
5	C	1.842728	0.94334	-0.48459
6	C	1.827236	0.43516	-1.96745
7	C	2.323969	-1.02279	-1.89674
8	C	1.862897	-1.50614	-0.52455
9	C	2.165439	-0.31294	0.366984
10	H	6.226558	3.518208	2.465235
11	H	6.739657	4.313806	0.959295
12	C	0.339782	0.435775	-2.34496
13	C	2.649706	1.27318	-2.99504
14	C	1.864366	2.341565	-3.7876
15	C	1.870013	1.907223	-5.12251
16	C	2.564464	0.705403	-5.24967
17	O	3.049872	0.3116	-4.03659
18	C	2.696475	0.083056	-6.45596
19	C	2.091073	0.701004	-7.59242
20	C	2.223722	0.016368	-8.92651
21	C	1.39807	1.887136	-7.47266
22	C	1.263703	2.531411	-6.21448
23	C	0.582818	3.732573	-5.90166
24	C	0.562094	4.189754	-4.60916
25	C	1.195547	3.522049	-3.5183
26	O	1.034694	4.133232	-2.32743
27	C	3.937497	1.797293	-2.37309

28	C	5.046451	1.970719	-3.20392
29	C	6.233478	2.443916	-2.6806
30	C	6.349354	2.747235	-1.32777
31	C	5.24684	2.585261	-0.49938
32	O	5.255873	2.861713	0.832046
33	C	6.449956	3.363654	1.413653
34	C	4.026823	2.111462	-1.02055
35	C	2.836989	2.063433	-0.10819
36	O	2.249961	3.377874	-0.12734
37	H	1.632978	-0.31297	1.316061
38	O	-0.16965	-0.03031	-3.32766
39	H	1.598971	3.790098	-1.60055
40	H	4.973789	1.723252	-4.2509
41	H	7.26976	2.647835	1.316609
42	H	7.287627	3.109919	-0.93843
43	H	7.090179	2.577264	-3.32777
44	H	3.172001	1.878529	0.909194
45	H	3.231748	-0.85101	-6.55868
46	H	3.273585	-0.10786	-9.20246
47	H	1.732177	0.585407	-9.71435
48	H	1.777744	-0.98077	-8.90058
49	H	0.944631	2.332999	-8.34885
50	H	0.078025	4.292821	-6.67836
51	H	0.046566	5.108407	-4.36279
52	H	-1.35094	1.220146	-1.35099
53	O	2.557686	-2.64427	-0.03558
54	H	3.236073	-0.32821	0.580014
55	H	3.413278	-1.04172	-1.92429
56	H	1.949011	-1.61142	-2.72931
57	H	0.784747	-1.70758	-0.53735
58	H	2.327598	-3.40919	-0.57293

Table S31: P3: TZ2P/B3LYP-D3(BJ)

1	H	2.716937	-2.24562	-0.56806
2	C	-0.63791	0.441921	-0.94938
3	H	2.992153	1.90947	0.913377
4	C	0.28268	1.146666	0.04718
5	C	1.721509	0.921262	-0.47865
6	C	1.552273	0.542466	-1.99187
7	C	1.878613	-0.97597	-2.01207
8	N	2.472723	-1.29254	-0.79596
9	C	2.326264	-0.33513	0.171394
10	H	6.054899	3.494192	2.504876
11	H	6.648243	4.255407	1.010268

12	C	0.046195	0.688269	-2.28834
13	C	2.503273	1.297361	-2.99705
14	C	1.819715	2.388915	-3.84874
15	C	1.867318	1.927297	-5.17247
16	C	2.503144	0.686549	-5.23923
17	O	2.920532	0.308517	-4.00103
18	C	2.654183	0.03003	-6.42566
19	C	2.140404	0.656764	-7.60167
20	C	2.297149	-0.06486	-8.91353
21	C	1.513131	1.884109	-7.54237
22	C	1.354136	2.563692	-6.30559
23	C	0.734771	3.810554	-6.04927
24	C	0.672591	4.293676	-4.76619
25	C	1.201488	3.606802	-3.63452
26	O	0.988687	4.228508	-2.45347
27	C	3.798936	1.783618	-2.35235
28	C	4.923244	1.928674	-3.17023
29	C	6.112015	2.382684	-2.6357
30	C	6.216018	2.705443	-1.28539
31	C	5.09853	2.57861	-0.47298
32	O	5.082974	2.893495	0.85065
33	C	6.291262	3.325083	1.458338
34	C	3.881657	2.110181	-1.00518
35	C	2.680026	2.068453	-0.11489
36	O	2.07331	3.376349	-0.19441
37	O	1.627855	-1.7741	-2.86854
38	O	2.611566	-0.46005	1.339081
39	H	1.462425	3.837354	-1.69247
40	H	4.86194	1.675094	-4.21626
41	H	7.067989	2.560908	1.379515
42	H	7.155198	3.058415	-0.88927
43	H	6.980094	2.489323	-3.2726
44	H	1.643321	3.594142	0.638048
45	H	3.137086	-0.93558	-6.48148
46	H	3.350526	-0.25004	-9.13647
47	H	1.872978	0.509984	-9.73555
48	H	1.799091	-1.03688	-8.88645
49	H	1.132056	2.335082	-8.44986
50	H	0.307254	4.385548	-6.86077
51	H	0.197306	5.244292	-4.56366
52	H	-0.17582	1.70021	-2.61721
53	H	-0.28152	-0.00523	-3.05919
54	H	-1.6489	0.856707	-0.93032
55	O	-0.68375	-0.97	-0.7273
56	H	0.161705	0.793432	1.071227

57	H	0.054277	2.211207	0.023641
58	H	-1.05958	-1.1393	0.142859

Table S32: P4: TZ2P/B3LYP-D3(BJ)

1	H	-2.65984	2.32600830	0.240796
2	C	-0.64074	0.575151	-0.87974
3	H	2.914317	1.90782	0.934041
4	C	0.436063	0.6887	0.211367
5	C	1.811534	0.782118	-0.51053
6	C	1.467053	0.680692	-2.02465
7	C	1.543848	-0.84853	-2.2529
8	N	2.380197	-1.35588	-1.26722
9	C	2.619998	-0.48513	-0.22077
10	H	5.901616	3.566449	2.594123
11	H	6.492391	4.369116	1.120726
12	C	0.014804	1.135097	-2.14626
13	C	2.505607	1.365714	-2.998
14	C	1.891999	2.43249	-3.9129
15	C	1.90816	1.893603	-5.20455
16	C	2.50763	0.632573	-5.20651
17	O	2.934353	0.316477	-3.94647
18	C	2.640668	-0.083	-6.35916
19	C	2.149495	0.504986	-7.56611
20	C	2.28005	-0.28661	-8.84023
21	C	1.575183	1.759522	-7.57097
22	C	1.442961	2.506621	-6.37001
23	C	0.920211	3.809473	-6.1784
24	C	0.911103	4.373279	-4.92535
25	C	1.397898	3.71202	-3.76072
26	O	1.310053	4.419327	-2.61327
27	C	3.779296	1.856292	-2.32102
28	C	4.910382	2.042621	-3.12006
29	C	6.075139	2.526367	-2.55917
30	C	6.144124	2.840223	-1.20434
31	C	5.019087	2.667952	-0.40953
32	O	4.974012	2.960801	0.916684
33	C	6.158902	3.420927	1.54904
34	C	3.829304	2.166758	-0.96848
35	C	2.615641	2.012666	-0.1062
36	O	1.853931	3.229356	-0.25833
37	O	0.955405	-1.50488	-3.06801
38	O	3.285881	-0.72472	0.754686
39	H	1.602634	3.924197	-1.82245
40	H	4.870568	1.803184	-4.1711

41	H	6.960071	2.682435	1.470366
42	H	7.065043	3.218152	-0.78884
43	H	6.950896	2.666929	-3.17866
44	H	1.134305	3.259453	0.381282
45	H	3.100175	-1.06154	-6.37245
46	H	3.324031	-0.53769	-9.04174
47	H	1.894632	0.269291	-9.69373
48	H	1.730384	-1.22843	-8.77272
49	H	1.223642	2.182804	-8.50338
50	H	0.533799	4.370496	-7.01976
51	H	0.520711	5.371856	-4.78026
52	H	-0.05137	2.220324	-2.12335
53	H	-0.44618	0.774681	-3.06391
54	O	-1.79111	1.293622	-0.44807
55	H	-0.90123	-0.47552	-1.03604
56	H	0.400122	-0.13888	0.916722
57	H	0.231292	1.589626	0.789208
58	H	-2.50654	1.149355	-1.07612

Table S33: P5 (12): TZ2P/B3LYP-D3(BJ)

1	O	2.865091	0.226531	-0.60924
2	C	2.174953	-0.96218	-0.24095
3	H	1.130874	-0.66701	-0.19927
4	C	2.555303	-1.5083	1.158311
5	C	1.394768	-2.35482	1.741399
6	C	2.056286	-3.4298	2.593069
7	H	1.409541	-4.30188	2.727513
8	O	2.356812	-2.8478	3.866007
9	C	3.315362	-3.7779	1.800945
10	C	3.821811	-2.43668	1.214278
11	C	4.761261	-1.72494	2.207117
12	N	4.179707	-0.52653	2.557512
13	C	2.880584	-0.34821	2.106771
14	O	2.14968	0.544753	2.444003
15	O	5.812154	-2.138	2.633289
16	C	4.533666	-2.62029	-0.1614
17	C	5.741388	-3.56489	-0.1747
18	C	6.868303	-2.78806	-0.46343
19	C	6.516153	-1.44965	-0.64141
20	O	5.170681	-1.28995	-0.46608
21	C	7.451713	-0.50488	-0.94441
22	C	8.811113	-0.9277	-1.06939
23	C	9.850129	0.114496	-1.39324
24	C	9.165256	-2.24952	-0.89778

25	C	8.188068	-3.23382	-0.58994
26	C	8.362185	-4.62367	-0.39884
27	C	7.273075	-5.42057	-0.13026
28	C	5.949418	-4.9189	-0.02407
29	O	4.90938	-5.78113	0.205282
30	C	3.501499	-2.85848	-1.26121
31	C	3.706707	-3.80838	-2.26015
32	C	2.767737	-3.96421	-3.26625
33	C	1.620294	-3.18263	-3.29601
34	C	1.414022	-2.2253	-2.30441
35	O	0.319929	-1.41759	-2.25754
36	C	-0.64363	-1.50138	-3.29626
37	C	2.356883	-2.05357	-1.27714
38	H	3.791815	-0.00115	-0.7641
39	H	0.850557	-2.83143	0.924159
40	H	0.694445	-1.74337	2.307457
41	H	2.882406	-3.46922	4.381011
42	H	3.04524	-4.45244	0.990568
43	H	4.079011	-4.2765	2.3963
44	H	4.58697	0.076913	3.257645
45	H	7.186811	0.534603	-1.08355
46	H	9.619861	0.620182	-2.33384
47	H	10.84192	-0.32673	-1.48056
48	H	9.888858	0.882577	-0.61723
49	H	10.20222	-2.54366	-1.00052
50	H	9.346591	-5.06851	-0.4682
51	H	7.42065	-6.48684	0.006098
52	H	5.245808	-6.68281	0.244033
53	H	4.59438	-4.42133	-2.2527
54	H	2.926729	-4.70253	-4.04129
55	H	0.901656	-3.31989	-4.08951
56	H	-1.1113	-2.48885	-3.33026
57	H	-0.19974	-1.27551	-4.26884
58	H	-1.39732	-0.75461	-3.06272

Table S34: P6: TZ2P/B3LYP-D3(BJ)

1	O	2.88572	0.2239	-0.59198
2	C	2.18172	-0.96229	-0.24439
3	H	1.140209	-0.66107	-0.19527
4	C	2.564832	-1.52721	1.139626
5	C	1.433847	-2.40607	1.70445
6	C	2.147815	-3.41143	2.605977
7	O	1.411642	-4.59791	2.871756
8	H	2.429479	-2.93199	3.551642

9	C	3.393444	-3.76043	1.802666
10	C	3.851762	-2.427	1.179989
11	C	4.802587	-1.6869	2.13191
12	N	4.166254	-0.53444	2.549861
13	C	2.861272	-0.38316	2.108905
14	O	2.101818	0.472084	2.482533
15	O	5.895255	-2.04235	2.494449
16	C	4.532608	-2.6122	-0.19896
17	C	5.730634	-3.55758	-0.21183
18	C	6.862557	-2.78218	-0.47653
19	C	6.517926	-1.4412	-0.65503
20	O	5.168198	-1.28126	-0.50907
21	C	7.463099	-0.49912	-0.92888
22	C	8.823064	-0.92901	-1.02404
23	C	9.871977	0.109913	-1.31718
24	C	9.169275	-2.25245	-0.85335
25	C	8.181678	-3.23453	-0.57493
26	C	8.343799	-4.62627	-0.38656
27	C	7.245534	-5.42072	-0.14835
28	C	5.922619	-4.9126	-0.06639
29	O	4.868006	-5.76131	0.126079
30	C	3.493541	-2.85254	-1.27852
31	C	3.685285	-3.81171	-2.26814
32	C	2.729837	-3.97623	-3.25687
33	C	1.582623	-3.19391	-3.27507
34	C	1.392982	-2.2268	-2.28986
35	O	0.301208	-1.41759	-2.2311
36	C	-0.68405	-1.52947	-3.24583
37	C	2.351998	-2.04697	-1.28308
38	H	3.806755	-0.01818	-0.76124
39	H	0.957165	-2.95532	0.890244
40	H	0.678657	-1.80521	2.209229
41	H	-1.43947	-0.78545	-3.01016
42	H	3.097844	-4.45188	1.016662
43	H	4.178816	-4.23263	2.386599
44	H	4.586721	0.098152	3.215757
45	H	7.207277	0.542336	-1.06757
46	H	9.674082	0.608574	-2.26879
47	H	10.86559	-0.33281	-1.36649
48	H	9.882245	0.882429	-0.54501
49	H	10.20731	-2.54872	-0.93317
50	H	9.327555	-5.07445	-0.4366
51	H	7.38476	-6.48856	-0.01872
52	H	5.191228	-6.6644	0.210279
53	H	4.572593	-4.42446	-2.26334

54	H	2.874038	-4.72321	-4.02603
55	H	0.850312	-3.34025	-4.05381
56	H	-1.14043	-2.52266	-3.24993
57	H	-0.26201	-1.31927	-4.23153
58	H	0.67502	-4.38654	3.453988

Table S35: P7: TZ2P/B3LYP-D3(BJ)

1	O	2.966808	0.316177	-0.63293
2	C	2.250711	-0.8543	-0.25521
3	H	1.211918	-0.54702	-0.19011
4	C	2.6479	-1.40324	1.131756
5	C	1.56798	-2.40599	1.547104
6	N	2.194069	-3.59069	1.894135
7	H	-1.15953	-2.43274	-3.06793
8	H	-0.33826	-1.31513	-4.19123
9	C	3.568962	-3.51701	1.919681
10	C	3.980286	-2.22105	1.190321
11	C	4.915928	-1.4306	2.123679
12	C	3.960514	-0.72339	3.086086
13	C	2.806164	-0.28664	2.193217
14	O	4.297708	-4.30446	2.468778
15	H	1.694107	-4.33834	2.352914
16	C	4.57815	-2.53114	-0.22413
17	C	5.736732	-3.51659	-0.18943
18	C	6.90509	-2.78496	-0.40688
19	C	6.606148	-1.44968	-0.69187
20	O	5.25223	-1.25947	-0.66877
21	C	7.596061	-0.5511	-0.95824
22	C	8.947273	-1.02236	-0.93317
23	C	10.04304	-0.03135	-1.2214
24	C	9.243823	-2.34165	-0.66257
25	C	8.211412	-3.28212	-0.39777
26	C	8.31009	-4.67069	-0.14371
27	C	7.170085	-5.42556	0.027243
28	C	5.862512	-4.87431	-0.01072
29	O	4.758379	-5.6706	0.06276
30	C	3.49995	-2.79676	-1.25587
31	C	3.648234	-3.79499	-2.21492
32	C	2.67055	-3.96631	-3.17972
33	C	1.548158	-3.14832	-3.21173
34	C	1.406072	-2.13751	-2.26368
35	O	0.354669	-1.27915	-2.22887
36	C	-0.69542	-1.44839	-3.16693

37	C	2.384569	-1.95793	-1.27514
38	H	3.873765	0.052869	-0.84192
39	H	2.778664	-4.74609	-3.92176
40	H	0.799683	-3.3	-3.974
41	H	-1.42669	-0.6791	-2.93655
42	H	4.515848	-4.4344	-2.20509
43	H	4.980071	-6.50142	0.496121
44	O	0.378585	-2.21871	1.586044
45	H	7.382855	0.483361	-1.19044
46	H	9.920105	0.409933	-2.21309
47	H	11.02424	-0.50149	-1.1792
48	H	10.02854	0.789394	-0.50063
49	H	10.27602	-2.66769	-0.6607
50	H	9.279116	-5.15119	-0.10577
51	H	7.26266	-6.494	0.185601
52	H	1.892161	-0.1214	2.759324
53	H	3.066671	0.633851	1.67439
54	H	4.438237	0.124242	3.584494
55	O	3.421311	-1.62159	4.063787
56	H	5.464121	-0.68088	1.55974
57	H	5.633473	-2.08012	2.622934
58	H	4.14261	-2.10461	4.481338

Table S36: P8: TZ2P/B3LYP-D3(BJ)

1	O	2.993119	0.329086	-0.62283
2	C	2.271847	-0.83817	-0.24811
3	H	1.233734	-0.52823	-0.18345
4	C	2.668456	-1.38786	1.137388
5	C	1.598859	-2.39804	1.554055
6	N	2.243579	-3.54995	1.971046
7	H	-1.15766	-2.4316	-3.0248
8	H	-0.34342	-1.32553	-4.16465
9	C	3.621854	-3.48187	1.948279
10	C	4.01537	-2.19451	1.194688
11	C	4.949347	-1.39313	2.113905
12	C	3.987165	-0.71347	3.094878
13	C	2.812027	-0.28454	2.208285
14	O	4.358187	-4.25788	2.500543
15	H	1.750204	-4.31511	2.408367
16	C	4.599705	-2.5187	-0.22246
17	C	5.75602	-3.50584	-0.18685
18	C	6.924002	-2.7786	-0.42052
19	C	6.625845	-1.44575	-0.71521

20	O	5.27163	-1.25289	-0.684
21	C	7.615339	-0.55168	-0.99705
22	C	8.965692	-1.02506	-0.97574
23	C	10.06166	-0.03839	-1.27788
24	C	9.261436	-2.34241	-0.69462
25	C	8.229208	-3.27821	-0.41438
26	C	8.326825	-4.66432	-0.14625
27	C	7.186781	-5.41492	0.041696
28	C	5.880414	-4.86095	0.007867
29	O	4.774083	-5.6545	0.10143
30	C	3.51193	-2.79156	-1.24189
31	C	3.649246	-3.80054	-2.19124
32	C	2.663769	-3.97824	-3.14706
33	C	1.54512	-3.15536	-3.18067
34	C	1.414554	-2.13267	-2.24369
35	O	0.364861	-1.27238	-2.20816
36	C	-0.69299	-1.44891	-3.13652
37	C	2.399872	-1.9474	-1.26303
38	H	3.896932	0.061074	-0.8398
39	H	2.763363	-4.76686	-3.88087
40	H	0.790421	-3.31222	-3.93569
41	H	-1.42126	-0.67639	-2.90753
42	H	4.514091	-4.44367	-2.18064
43	H	4.994416	-6.47097	0.562134
44	O	0.40491	-2.23585	1.566931
45	H	7.402311	0.480932	-1.23757
46	H	9.933126	0.396644	-2.27163
47	H	11.04204	-0.51045	-1.23881
48	H	10.0536	0.786812	-0.56211
49	H	10.29317	-2.66974	-0.6946
50	H	9.295179	-5.14616	-0.10921
51	H	7.27824	-6.48162	0.211365
52	H	1.890312	-0.11696	2.761206
53	H	3.073546	0.642315	1.695308
54	O	4.563169	0.337915	3.849948
55	H	5.687794	-2.02429	2.600871
56	H	5.46417	-0.62325	1.542269
57	H	3.654776	-1.43438	3.844572
58	H	4.832087	1.039328	3.245327

Table S37: I1⁺: TZ2P/B3LYP-D3(BJ)

1	C	-1.74627	2.56627	2.923235
2	C	-1.09757	1.540328	3.605042
3	C	-0.25119	0.673909	2.941898
4	C	-0.02099	0.868224	1.571187
5	C	-0.66407	1.897173	0.874942
6	C	-1.54429	2.7446	1.550513
7	C	-0.45763	1.941706	-0.60727
8	O	-1.18173	0.846197	-1.17976
9	C	0.933209	0.025752	0.882982
10	C	0.839452	-1.37806	1.01089
11	C	-0.47271	-1.93901	0.851095
12	C	-0.61381	-3.24636	0.345474
13	C	0.474275	-4.04723	0.159046
14	C	1.774807	-3.63795	0.54828
15	C	1.94694	-2.3154	1.024563
16	C	3.174525	-2.01949	1.641867
17	C	4.196722	-2.94981	1.669782
18	C	4.062906	-4.21812	1.092787
19	C	2.837755	-4.55624	0.5517
20	C	5.22035	-5.17399	1.096301
21	O	-1.55858	-1.19923	1.050583
22	O	3.303418	-0.8054	2.238376
23	O	-2.15461	3.694224	0.81012
24	C	-3.08786	4.571422	1.442868
25	H	-2.41762	3.215413	3.463867
26	H	-1.26659	1.424023	4.666648
27	H	0.265376	-0.11745	3.464559
28	H	-0.82576	2.880883	-1.01406
29	H	-1.60825	-3.607	0.114229
30	H	0.340645	-5.04706	-0.23337
31	H	5.132864	-2.682	2.147188
32	H	2.671677	-5.55039	0.158999
33	H	5.620249	-5.30279	2.103161
34	H	4.929498	-6.15113	0.716088
35	H	6.030325	-4.7946	0.470194
36	H	-2.33463	-1.6013	0.638296
37	H	4.153716	-0.74884	2.688966
38	H	-3.45155	5.227719	0.659094
39	H	-3.92209	4.010929	1.868021
40	H	-2.60256	5.163923	2.220423
41	C	3.130327	2.880229	-0.15336
42	C	1.724968	3.210443	-0.63785
43	C	1.048795	1.846244	-0.89342

44	C	1.868469	0.784774	-0.03515
45	C	2.930445	1.64917	0.71843
46	C	1.236396	1.382492	-2.33576
47	N	2.039661	0.267954	-2.33889
48	C	2.565903	-0.07879	-1.11008
49	H	2.388435	-0.14096	-3.19535
50	O	0.73989	1.896739	-3.30677
51	O	3.430268	-0.89528	-0.94576
52	O	3.980247	2.485828	-1.23
53	H	1.180793	3.735233	0.149891
54	H	1.709109	3.837367	-1.52829
55	H	2.535309	1.937341	1.690282
56	H	3.851634	1.097977	0.859617
57	H	4.166277	3.245384	-1.79248
58	H	3.580584	3.699998	0.411192
59	H	-1.29475	1.030273	-2.12093

Table S38: TS10: TZ2P/B3LYP-D3(BJ)

1	C	-1.69847	3.421413	2.356836
2	C	-0.99901	2.610341	3.240587
3	C	-0.24854	1.545755	2.790732
4	C	-0.14962	1.259485	1.416905
5	C	-0.725	2.187423	0.503442
6	C	-1.54935	3.218267	0.988133
7	C	-0.46612	2.152951	-0.97586
8	O	-1.46489	1.392948	-1.64458
9	C	0.440407	0.068302	0.804064
10	C	0.261722	-1.35855	1.078842
11	C	-0.26327	-2.03504	-0.05392
12	C	-0.21228	-3.41674	-0.24065
13	C	0.528449	-4.16009	0.625401
14	C	1.045881	-3.59492	1.811822
15	C	0.744924	-2.23374	2.155633
16	C	1.023217	-1.91373	3.50078
17	C	1.795654	-2.72179	4.311329
18	C	2.255958	-3.97382	3.890625
19	C	1.811441	-4.4137	2.663364
20	C	3.116108	-4.81737	4.785086
21	O	-0.85719	-1.25015	-0.96485
22	O	0.483288	-0.81521	4.104251
23	O	-2.12832	3.994854	0.049578
24	C	-2.96663	5.076005	0.462627
25	H	-2.30695	4.228498	2.733666
26	H	-1.03289	2.824365	4.299987

27	H	0.35157	0.998636	3.48775
28	H	-0.50593	3.18137	-1.33175
29	H	-0.67908	-3.8566	-1.11207
30	H	0.700785	-5.21087	0.435702
31	H	1.970564	-2.38272	5.324137
32	H	2.042373	-5.41639	2.328692
33	H	2.68145	-4.90244	5.781907
34	H	3.246434	-5.81933	4.380777
35	H	4.10453	-4.36786	4.89987
36	H	-1.10964	-1.74371	-1.75574
37	H	-0.42087	-0.6962	3.791315
38	H	-3.31513	5.541562	-0.45333
39	H	-3.82016	4.708558	1.03421
40	H	-2.40598	5.802833	1.053011
41	C	3.251326	1.986729	-0.44634
42	C	2.004687	2.742986	-0.8987
43	C	0.961533	1.647949	-1.211
44	C	1.356498	0.41852	-0.3476
45	C	2.696658	0.843971	0.401649
46	C	1.095263	1.162831	-2.653
47	N	1.497059	-0.1562	-2.62809
48	C	1.760668	-0.6613	-1.37338
49	H	1.778364	-0.6478	-3.46544
50	O	0.878365	1.814997	-3.64056
51	O	2.26766	-1.72788	-1.14732
52	O	3.937204	1.389984	-1.54161
53	H	1.639259	3.367336	-0.08088
54	H	2.181974	3.38662	-1.75895
55	H	2.477573	1.209306	1.402153
56	H	3.373332	-0.00277	0.476403
57	H	4.359337	2.073157	-2.07409
58	H	3.928849	2.61139	0.140368
59	H	-1.60264	1.782269	-2.51561

Table S39: I9: TZ2P/B3LYP-D3(BJ)

1	C	-0.12213	2.640881	3.098108
2	C	0.847939	1.692268	3.405758
3	C	1.226362	0.733306	2.483148
4	C	0.625926	0.733126	1.223543
5	C	-0.30588	1.712773	0.875685
6	C	-0.70158	2.65929	1.827966
7	C	-0.82323	1.711014	-0.53689
8	O	-1.67396	0.579537	-0.711
9	C	0.969796	-0.23095	0.15519

10	C	0.935689	-1.58349	0.405132
11	C	1.394474	-2.56424	-0.55604
12	C	2.278045	-3.5743	-0.1552
13	C	2.42953	-3.81765	1.18452
14	C	1.596111	-3.1993	2.160119
15	C	0.720075	-2.16277	1.750644
16	C	-0.32254	-1.82642	2.623933
17	C	-0.3704	-2.38312	3.899568
18	C	0.573456	-3.30648	4.354048
19	C	1.545189	-3.72512	3.461677
20	C	0.488069	-3.85661	5.749456
21	O	0.911267	-2.4827	-1.77001
22	O	-1.28884	-0.99732	2.176872
23	O	-1.62605	3.561472	1.427396
24	C	-2.06025	4.560956	2.348644
25	H	-0.4036	3.371062	3.840706
26	H	1.315111	1.713657	4.380955
27	H	1.986894	0.007804	2.728106
28	H	-1.36816	2.630392	-0.73903
29	H	2.771906	-4.18623	-0.89856
30	H	3.10633	-4.59783	1.508756
31	H	-1.19022	-2.10266	4.551998
32	H	2.247611	-4.49791	3.743692
33	H	-0.47789	-4.3336	5.922249
34	H	1.267892	-4.59281	5.933274
35	H	0.593968	-3.0593	6.487032
36	H	1.423987	-3.00606	-2.40767
37	H	-1.9614	-0.85673	2.85264
38	H	-2.79114	5.157846	1.812717
39	H	-2.52932	4.108256	3.224056
40	H	-1.22792	5.194369	2.661042
41	C	2.649789	2.648694	-1.51221
42	C	1.161978	2.997414	-1.46686
43	C	0.40169	1.646663	-1.46639
44	C	1.464463	0.558542	-1.05577
45	C	2.749549	1.371727	-0.68275
46	C	-0.01265	1.241138	-2.87573
47	N	0.826997	0.225336	-3.29722
48	C	1.782382	-0.15141	-2.38304
49	H	0.851752	-0.08178	-4.25993
50	O	-0.9074	1.720717	-3.52304
51	O	2.726831	-0.86765	-2.60632
52	O	3.080634	2.331219	-2.83395
53	H	0.940369	3.523928	-0.53738
54	H	0.851447	3.638382	-2.29061

55	H	2.739014	1.627606	0.372405
56	H	3.6489	0.802962	-0.90458
57	H	3.091668	3.130684	-3.37095
58	H	3.269349	3.443004	-1.08986
59	H	-2.20549	0.723094	-1.50371

Table S40: TS11: TZ2P/B3LYP-D3(BJ)

1	C	-1.84521	1.804388	2.621103
2	C	-1.25988	0.597133	2.988461
3	C	-0.44298	-0.08983	2.113114
4	C	-0.17667	0.446687	0.842862
5	C	-0.7108	1.685853	0.489074
6	C	-1.56954	2.355996	1.369348
7	C	-0.32391	2.272294	-0.83046
8	O	-1.06285	1.650412	-1.87611
9	C	0.731769	-0.26534	-0.08248
10	C	0.535634	-1.65182	-0.16076
11	C	1.610435	-2.5875	-0.37359
12	C	1.647913	-3.80752	0.323594
13	C	0.556084	-4.23182	1.024272
14	C	-0.67658	-3.53234	0.968807
15	C	-0.72005	-2.28781	0.2927
16	C	-2.00013	-1.78207	-0.00051
17	C	-3.13944	-2.40836	0.489131
18	C	-3.08257	-3.56952	1.264442
19	C	-1.83952	-4.13466	1.479491
20	C	-4.34069	-4.18793	1.803094
21	O	2.576543	-2.24791	-1.18188
22	O	-2.06943	-0.69303	-0.78598
23	O	-2.07349	3.531314	0.932291
24	C	-2.94133	4.272565	1.789252
25	H	-2.49388	2.314033	3.316465
26	H	-1.44792	0.196045	3.974835
27	H	0.014469	-1.01752	2.417531
28	H	-0.52846	3.34128	-0.8253
29	H	2.543773	-4.41263	0.265431
30	H	0.596159	-5.1713	1.560438
31	H	-4.10572	-1.98156	0.243856
32	H	-1.75206	-5.07541	2.006299
33	H	-5.08224	-4.3188	1.014108
34	H	-4.14437	-5.15955	2.252003
35	H	-4.78742	-3.54777	2.566516
36	H	3.35283	-2.82481	-1.13977
37	H	-2.97841	-0.43119	-0.96926

38	H	-3.21155	5.164614	1.233607
39	H	-3.84126	3.700908	2.02311
40	H	-2.4322	4.556088	2.712295
41	C	3.203731	2.476644	0.393345
42	C	1.922586	3.141775	-0.09917
43	C	1.196532	2.097651	-0.98259
44	C	1.829614	0.688813	-0.57672
45	C	2.793631	1.021816	0.610665
46	C	1.529047	2.264248	-2.4598
47	N	2.303829	1.204064	-2.85603
48	C	2.752495	0.410105	-1.81354
49	H	2.803317	1.218658	-3.73559
50	O	1.164421	3.175332	-3.16174
51	O	3.762387	-0.23087	-1.86265
52	O	4.222771	2.493998	-0.60162
53	H	1.285705	3.378179	0.755184
54	H	2.105224	4.068614	-0.64094
55	H	2.264026	0.937165	1.556459
56	H	3.647244	0.348342	0.613023
57	H	4.531974	3.397291	-0.72914
58	H	3.567667	2.923529	1.321654
59	H	-0.98972	2.215341	-2.65588

Table S41: I5⁺: TZ2P/B3LYP-D3(BJ)

1	C	-2.04655	1.967506	2.716087
2	C	-1.60291	0.702573	3.088302
3	C	-0.71185	0.011812	2.297761
4	C	-0.22686	0.596203	1.106943
5	C	-0.66996	1.878198	0.725853
6	C	-1.58722	2.557794	1.534318
7	C	-0.2626	2.473952	-0.58865
8	O	-1.21249	2.079238	-1.58187
9	C	0.754622	-0.11827	0.34012
10	C	0.75719	-1.56116	0.397684
11	C	1.893375	-2.28496	0.808656
12	C	1.823789	-3.662	1.084969
13	C	0.651381	-4.34219	0.946001
14	C	-0.51403	-3.70643	0.456667
15	C	-0.45587	-2.31993	0.152592
16	C	-1.55865	-1.79287	-0.56252
17	C	-2.67188	-2.56194	-0.83515
18	C	-2.76096	-3.90576	-0.44211
19	C	-1.66873	-4.46546	0.184537
20	C	-4.00099	-4.6978	-0.74101

21	O	3.017802	-1.6193	1.109465
22	O	-1.44586	-0.51551	-0.98612
23	O	-1.98855	3.768396	1.09806
24	C	-2.94027	4.506782	1.866332
25	H	-2.7525	2.486652	3.345882
26	H	-1.95922	0.266186	4.010944
27	H	-0.34606	-0.9556	2.602883
28	H	-0.29634	3.558252	-0.49585
29	H	2.711172	-4.16461	1.449548
30	H	0.608886	-5.3969	1.183519
31	H	-3.49324	-2.11088	-1.3804
32	H	-1.6678	-5.51697	0.438862
33	H	-4.22946	-4.6771	-1.80787
34	H	-3.89054	-5.7366	-0.43642
35	H	-4.86265	-4.28034	-0.21656
36	H	3.709116	-2.23259	1.387496
37	H	-2.1488	-0.23902	-1.58526
38	H	-3.10569	5.428788	1.319023
39	H	-3.87999	3.958767	1.95193
40	H	-2.54927	4.735761	2.859157
41	C	3.472493	2.409732	-0.16176
42	C	2.145272	3.135596	-0.30075
43	C	1.193993	2.095851	-0.92373
44	C	1.727361	0.685111	-0.46881
45	C	3.069983	1.019355	0.298527
46	C	1.348722	2.0677	-2.44615
47	N	1.773266	0.815549	-2.82553
48	C	2.05793	-0.04747	-1.79303
49	H	1.97957	0.583113	-3.78759
50	O	1.103568	2.98246	-3.1911
51	O	2.479256	-1.16538	-1.91042
52	O	4.124681	2.252964	-1.42059
53	H	1.773567	3.415796	0.687575
54	H	2.199209	4.037601	-0.90883
55	H	2.877012	1.022084	1.368807
56	H	3.831738	0.281377	0.085592
57	H	4.42026	3.110992	-1.74427
58	H	4.1395	2.884862	0.561546
59	H	-1.13003	2.696606	-2.31971

REFERENCES

(1) Kieser, T.; Bibb, M. J.; Buttner, M. J.; Chater, K. F.; Hopwood, D. A. *Practical streptomyces genetics*; John Innes Foundation Norwich, 2000.
(2) Zhu, H.; Swierstra, J.; Wu, C.; Girard, G.; Choi, Y. H.; Van Wamel, W.; Sandiford, S. K.; van Wezel, G. P. Eliciting antibiotics active against the ESKAPE pathogens in a collection of actinomycetes isolated from mountain soils. *Microbiology* **2014**, *160* (8), 1714-1725.
(3) Wu, C.; van der Heul, H. U.; Melnik, A. V.; Lübben, J.; Dorrestein, P. C.; Minnaard, A. J.; Choi, Y. H.; van Wezel, G. P. Lugdunomycin, an Angucycline-Derived Molecule with Unprecedented Chemical Architecture. *Angew Chem Int Ed Engl* **2019**, *58* (9), 2809-2814. DOI: https://doi.org/10.1002/anie.201814581.
(4) Elsayed, S. S.; van der Heul, H. U.; Xiao, X.; Nuutila, A.; Baars, L. R.; Wu, C.; Metsä-Ketelä, M.; van Wezel, G. P. Unravelling key enzymatic steps in C-ring cleavage during angucycline biosynthesis. *Comms Chem* **2023**, *6* (1), 281.
(5) Nuñez Santiago, I.; Machushynets, N. V.; Mladic, M.; van Bergeijk, D. A.; Elsayed, S. S.; Hankemeier, T.; van Wezel, G. P. nanoRAPIDS as an analytical pipeline for the discovery of novel bioactive metabolites in complex culture extracts at the nanoscale. *Comms Chem* **2024**, *7* (1), 71.
(6) Uiterweerd, M. T.; Santiago, I. N.; van der Heul, H. U.; van Wezel, G. P.; Minnaard, A. J. Iso-maleimycin, a Constitutional Isomer of Maleimycin, from *Streptomyces* sp. QL37. *Eur J Org Chem* **2020**, *2020* (32), 5145-5152. DOI: https://doi.org/10.1002/ejoc.202000767.
(7) Uiterweerd, M. T.; Minnaard, A. J. Racemic Total Synthesis of Elmonin and Pratenone A, from Streptomyces, Using a Common Intermediate Prepared by peri-Directed C–H Functionalization. *Organic letters* **2022**, *24* (51), 9361-9365.
(8) Uiterweerd, M. T.; Santiago, I. N.; van der Heul, H.; van Wezel, G. P.; Minnaard, A. J. Iso-maleimycin, a constitutional isomer of maleimycin, from Streptomyces sp. QL37. *Eur J Org Chem* **2020**, *2020* (32), 5145-5152.
(9) Pluskal, T.; Castillo, S.; Villar-Briones, A.; Oresic, M. MZmine 2: modular framework for processing, visualizing, and analyzing mass spectrometry-based molecular profile data. *BMC Bioinformatics* **2010**, *11*, 395. DOI: 10.1186/1471-2105-11-395.
(10) Xia, J.; Mandal, R.; Sinelnikov, I. V.; Broadhurst, D.; Wishart, D. S. MetaboAnalyst 2.0--a comprehensive server for metabolomic data analysis. *Nucleic Acids Res* **2012**, *40* (Web Server issue), W127-133. DOI: 10.1093/nar/gks374.
(11) Machushynets, N. V.; Elsayed, S. S.; Du, C.; Siegler, M. A.; de la Cruz, M.; Genilloud, O.; Hankemeier, T.; van Wezel, G. P. Discovery of actinomycin L, a new member of the actinomycin family of antibiotics. *Sci Rep* **2022**, *12* (1), 2813. DOI: 10.1038/s41598-022-06736-0.
(12) Van Bergeijk, D. A.; Elsayed, S. S.; Du, C.; Nunez Santiagio, I.; Roseboom, A.; Zhang, L.; Carrion, V. J.; Spaink, H. P.; van Wezel, G. P. The ubiquitous catechol moiety elicits siderophore and angucycline production in *Streptomyces*. *Comms Chem* **2022**, *5*, 14.
(13) Gubbens, J.; Zhu, H.; Girard, G.; Song, L.; Florea, B. I.; Aston, P.; Ichinose, K.; Filippov, D. V.; Choi, Y. H.; Overkleeft, H. S.; et al. Natural product proteomining, a quantitative proteomics platform, allows rapid discovery of biosynthetic gene clusters for different classes of natural products. *Chem Biol* **2014**, *21* (6), 707-718, DOI: 10.1016/j.chembiol.2014.03.011.
(14) Wu, C.; van der Heul, H. U.; Melnik, A. V.; Lübben, J.; Dorrestein, P. C.; Minnaard, A. J.; Choi, Y. H.; van Wezel, G. P. Lugdunomycin, an Angucycline-Derived Molecule with Unprecedented Chemical Architecture. *Angew Chem Intl Ed* **2019**, *131* (9), 2835-2840.
(15) Te Velde, G. t.; Bickelhaupt, F. M.; Baerends, E. J.; Fonseca Guerra, C.; van Gisbergen, S. J.; Snijders,

J. G.; Ziegler, T. Chemistry with ADF. *J Comp Chem* **2001**, *22* (9), 931-967.
(16) Rüger, R.; Franchini, M.; Trnka, T.; Yakovlev, A.; van Lenthe, E.; Philipsen, P.; van Vuren, T.; Klumpers, B.; Soini, T. Amsterdam Modeling Suite (AMS version: 2021.107), Software for Chemistry & Materials SCM, Theoretical Chemistry, Vrije Universiteit, Amsterdam, The Netherlands.
(17) van der Heul, H. Analysis of the angucycline biosynthetic gene cluster in *Streptomyces* sp. QL37 and implications for lugdunomycin production. PhD thesis, Leiden University, 2022.
(18) Gregory, M. A.; Till, R.; Smith, M. C. Integration site for Streptomyces phage ϕBT1 and development of site-specific integrating vectors. *J Bacteriol* **2003**, *185* (17), 5320-5323.
(19) Swiatek, M. A.; Tenconi, E.; Rigali, S.; van Wezel, G. P. Functional analysis of the N-acetylglucosamine metabolic genes of *Streptomyces coelicolor* and role in the control of development and antibiotic production. *J Bacteriol* **2012**, *194* (5), 1136-1144. DOI: 10.1128/JB.06370-11.
(20) Vara, J.; Lewandowska-Skarbek, M.; Wang, Y.-G.; Donadio, S.; Hutchinson, C. Cloning of genes governing the deoxysugar portion of the erythromycin biosynthesis pathway in *Saccharopolyspora erythraea* (*Streptomyces erythreus*). *J Bacteriol* **1989**, *171* (11), 5872-5881.
(21) Zhou, H.; Wang, Y.; Yu, Y.; Bai, T.; Chen, L.; Liu, P.; Guo, H.; Zhu, C.; Tao, M.; Deng, Z. A non-restricting and non-methylating *Escherichia coli* strain for DNA cloning and high-throughput conjugation to *Streptomyces coelicolor*. *Curr Microbiol* **2012**, *64*, 185-190.
(22) Asnicar, F.; Thomas, A. M.; Beghini, F.; Mengoni, C.; Manara, S.; Manghi, P.; Zhu, Q.; Bolzan, M.; Cumbo, F.; May, U. Precise phylogenetic analysis of microbial isolates and genomes from metagenomes using PhyloPhlAn 3.0. *Nat Commun* **2020**, *11* (1), 2500.
(23) Ose, T.; Watanabe, K.; Mie, T.; Honma, M.; Watanabe, H.; Yao, M.; Oikawa, H.; Tanaka, I. Insight into a natural Diels-Alder reaction from the structure of macrophomate synthase. *Nature* **2003**, *422* (6928), 185-189. DOI: 10.1038/nature01454.
(24) Guimaraes, C. R.; Udier-Blagovic, M.; Jorgensen, W. L. Macrophomate synthase: QM/MM simulations address the Diels-Alder versus Michael-Aldol reaction mechanism. *J Am Chem Soc* **2005**, *127* (10), 3577-3588. DOI: 10.1021/ja043905b.
(25) Serafimov, J. M.; Gillingham, D.; Kuster, S.; Hilvert, D. The putative Diels-Alderase macrophomate synthase is an efficient aldolase. *J Am Chem Soc* **2008**, *130* (25), 7798-7799, DOI: 10.1021/ja8017994.
(26) Hubbard, B. K.; Koch, M.; Palmer, D. R.; Babbitt, P. C.; Gerlt, J. A. Evolution of enzymatic activities in the enolase superfamily: characterization of the (D)-glucarate/galactarate catabolic pathway in *Escherichia coli*. *Biochemistry* **1998**, *37* (41), 14369-14375, DOI: 10.1021/bi981124f.
(27) Jumper, J.; Evans, R.; Pritzel, A.; Green, T.; Figurnov, M.; Ronneberger, O.; Tunyasuvunakool, K.; Bates, R.; Zidek, A.; Potapenko, A.; et al. Highly accurate protein structure prediction with AlphaFold. *Nature* **2021**, *596* (7873), 583-589, DOI: 10.1038/s41586-021-03819-2.
(28) Eberhardt, J.; Santos-Martins, D.; Tillack, A. F.; Forli, S. AutoDock Vina 1.2.0: New Docking Methods, Expanded Force Field, and Python Bindings. *J Chem Inf Model* **2021**, *61* (8), 3891-3898. DOI: 10.1021/acs.jcim.1c00203.
(29) Van Der Spoel, D.; Lindahl, E.; Hess, B.; Groenhof, G.; Mark, A. E.; Berendsen, H. J. GROMACS: fast, flexible, and free. *J Comp Chem* **2005**, *26* (16), 1701-1718.
(30) Tian, C.; Kasavajhala, K.; Belfon, K. A. A.; Raguette, L.; Huang, H.; Migues, A. N.; Bickel, J.; Wang, Y.; Pincay, J.; Wu, Q.; Simmerling, C. ff19SB: Amino-Acid-Specific Protein Backbone Parameters Trained against Quantum Mechanics Energy Surfaces in Solution. *J Chem Theory Comput* **2020**, *16* (1), 528-552. DOI: 10.1021/acs.jctc.9b00591.
(31) Izadi, S.; Anandakrishnan, R.; Onufriev, A. V. Building Water Models: A Different Approach. *J Phys Chem Lett* **2014**, *5* (21), 3863-3871. DOI: 10.1021/jz501780a
(32) Bussi, G.; Donadio, D.; Parrinello, M. Canonical sampling through velocity rescaling. *The J Chem Phys* **2007**, *126* (1).
(33) Parrinello, M.; Rahman, A. Polymorphic transitions in single crystals: A new molecular dynamics

method. *J Appl Phys* **1981**, *52* (12), 7182-7190.
(34) Allen, M. P.; Tildesley, D. J. *Computer simulation of liquids*; Oxford university press, 2017.
(35) Darden, T.; York, D.; Pedersen, L. Particle mesh Ewald: An N· log (N) method for Ewald sums in large systems. *J Chem Phys* **1993**, *98* (12), 10089-10092.
(36) Laskowski, R. A.; Swindells, M. B. LigPlot+: multiple ligand-protein interaction diagrams for drug discovery. *J Chem Inf Model* **2011**, *51* (10), 2778-2786. DOI: 10.1021/ci200227u.
(37) Andersen, C. A.; Palmer, A. G.; Brunak, S.; Rost, B. Continuum secondary structure captures protein flexibility. *Structure* **2002**, *10* (2), 175-184. DOI: 10.1016/s0969-2126(02)00700-1.
(38) Tang, S.-Y.; Shi, J.; Guo, Q.-X. Accurate prediction of rate constants of Diels–Alder reactions and application to design of Diels–Alder ligation. *Org Biomol Chem* **2012**, *10* (13), 2673-2682.
